# Abstracts of 11th C1-inhibitor Deficiency & Angioedema Workshop

**DOI:** 10.1186/s13223-019-0355-0

**Published:** 2019-08-13

## I0

Dear Colleagues,

Between 23 and 26 May 2019, the international scientific conference on angioedemas—conditions belonging to the group of orphan diseases and resulting in a life-threatening condition—will be held on the eleventh occasion in Budapest (2019.haenetworkshop.hu). This year, the word ‘angioedema’ has been added to the title of this event (organised every two years in Hungary starting from 1999), which now reads “11th C1-INH Deficiency & Angioedema Workshop”. This designation better reflects the agenda of the 4-day long conference, because it has become a forum for discussing topics also on other bradykinin-mediated hereditary and acquired angioedemas, in addition to C1-inhibitor deficiency. Based on the 82 submitted abstracts, the programme includes 36 oral and 46 poster presentations. These will be supplemented by the lectures delivered by Patricia Pozo-Rosich (Barcelona, Spain) on the relationship between migraine and angioedema, as well as by Michael Kirschfink (Heidelberg, Germany) on the standardization of the laboratory methods applied for complement testing. Furthermore, Avner Reshef (Ashkelon, Israel) will summarize the prodromal symptoms of angioedema. By tradition, the agenda of the conference comprises consensus meetings and roundtable discussions intended to facilitate the adoption of international guidelines. This year, Anastasios E. Germenis will moderate the roundtable session *“International consensus on the use of genetics in the management of hereditary angioedema”*, whereas Teresa Caballero will lead the discussion on *“International consensus on the gynecologic and obstetric management of female patients with hereditary angioedema*—*updates”*. Further, the ‘HAE Global Registry’ Work Group (established to develop an international database on hereditary angioedema), led by Marco Cicardi, will hold its meeting here.

The geographical distribution and professional background of the participants are variegated. Approx. 330 delegates have registered from all over the world, from 39 countries in total. Researchers, medical professionals, drug development specialists, members of patients’ organisations, and nurses will all be represented in the audience, which has become an active and creative community over the past two decades. The delegates of the International Hereditary Angioedema Nursing Organization will hold a consultative meeting on improving the state-of-the-art care of angioedema patients in the ‘Nurse Meeting’ session of the Workshop, under the moderation by Iris Leibovich-Nassi.

Generous support by CSL Behring, Pharming, Biocryst, Kalvista and Sobi made it possible to organize a high level scientific conference. This educational activity is supported by an independent medical educational grant from Shire (Takeda). The support referred to above has again enabled us to present the “*For HAE Patients*” award, as well as to give the “*Grant for Young Investigators*” to four presenters under the age of 35 years. In 2019, the ‘*For HAE Patients*’ award goes to Teresa Caballero (Madrid, Spain), who will deliver a presentation in the festive session of the first conference day, and her accomplishments will be reviewed by Henriette Farkas (Budapest, Hungary) beforehand. The closing feature of the Workshop will be the presentation of the *‘Grant for Young Investigators’* to the four winners (elected by a 7-member jury) by Peter J. Späth, Chair of the Jury. Finally, Allen Kaplan will summarize for the audience all the topics and findings discussed at this Workshop.

The programme and the submitted abstracts including those by the invited speakers will be published again in the greatly esteemed *Allergy, Asthma, & Clinical Immunology*, in order to make them available to an even broader range of professionals interested in this subject.

**Table:** 

Henriette Farkas and Lilian Varga	
Chairs of 11th C1-inhibitor Deficiency & Angioedema Workshop	

## Invited lectures

### I1 Which are the clinical, pathophysiological and therapeutic similarities between migraine & hereditary angioedema with C1-inhibitor deficiency?

#### Patricia Pozo-Rosich^1,2,*^

##### ^1^Headache Unit, Neurology Department, Valld’Hebron University Hospital, Barcelona; ^2^Headache Research Group, Valld’Hebron Institute of Research (VHIR), Universitat Autònoma of Barcelona, Spain

###### **Correspondence:** Patricia Pozo-Rosich (ppozorosich@yahoo.com)

*Allergy, Asthma & Clinical Immunology* 2019, **15(Suppl 4):**I1

Migraine is the second most disabling chronic neurological disease.

Pathophysiologically, migraine is a genetically-driven, cycling functional disorder affecting several areas of the brain, ultimately leading to an excessive activation of trigeminovascular afferents in the meninges. Through multiple pathways local release of inflammatory peptides as calcitonin gene-related peptide (CGRP), substance P, VIP and bradykinin are presumable.

Neurological symptoms of C1-INH-HAE (hereditary angioedema with C1-inhibitor deficiency) as cephalalgia, hemiplegia, and migraine symptoms are similar, which might be due to the role that bradykinin plays. The unpredictable frequency of the attacks is ruled by the extremely variable efficiency of the fine-tuned interplay of the brain with dynamic stimuli originating from the internal or external environment. Unlike several other chronic neurological diseases, migraine can be treated and prevented. However, not all patients respond to treatment.

Both migraine and C1-INH-HAE are genetically-driven paroxysmal disabling diseases, which are characterized by recurrent unpredictable episodic attacks. In rare cases, C1-INH-HAE is manifested with neurological symptoms, including cephalalgia which does not respond to conventional treatment. The complement system has important function in the regulation of bradykinin release within the brain. Some of the inflammatory molecules in migraine can be measured indirectly (serum, CSF) using ELISAs. This is different from C1-INH-HAE, where conventional laboratory tests are of diagnostic value.

In respect of treatment options: in migraine, treatment is broad, and recently a new class of drugs has been developed, and proven to be effective and safe, targeting CGRP and preventing migraine attacks, however, there may be non-responders.

The pathophysiology shares the presence of inflammatory molecules which lead to pain in migraine and angioedema attacks in C1-INH-HAE. Therapeutic options of both diseases are to be personalized and target-driven.

### I2 Analysis of C1-inhibitor deficiency: need for standardization and quality control

#### Michael Kirschfink

##### Institute of Immunology, University of Heidelberg, Germany

###### **Correspondence:** Michael Kirschfink (kirschfink@uni-hd.de)

*Allergy, Asthma & Clinical Immunology* 2019, **15(Suppl 4):**I2

Deficiency of C1-INH results in episodic angioedema without urticarial that is inherited (hereditary angioedema, [C1-INH-HAE]) or acquired (C1-INH-AA). In addition to its role as an inhibitor of C1r and C1s of the classical pathway and MASP1 and MASP2 of the lectin pathway, C1-INH is the major inhibitor of factor XIIa and kallikrein. The lack of inhibition of these enzymes results in excessive bradykinin generation, which in turn mediates increases vascular permeability, leading to angioedema.

In the past decades complement analysis has undergone a tremendous development in part expanding beyond specialized laboratories. However, improving diagnostic strategies requires the correct choice of analytes and the use of well-characterized methods that will yield consistent results between laboratories.

In the diagnosis of the various types of angioedema, as in many fields of immunodiagnostic, there is considerable need for consensus and standardization of analytical methods, which will be a major challenge in the future. In recent years, laboratories specializing in complement analysis have joined with the International Complement Society and the IUIS to coordinate efforts to standardize and improve complement testing, ongoing efforts show first promising results (http://iuisonline.org/index.php?option=com_content&view=article&id=64&Itemid=69). Since that time eight rounds of external quality assessment, now covering 18 parameters, also including those to better characterize angioedema patients (C4, C1-inhibitor [protein, function], autoantibodies to C1-inhibitor) have been completed. It is recommended to extend this efforts to a more comprehensive analysis of parameters of the clotting and kallikrein-kinin systems for better defining the pathophysiological background and to distinguish angioedema with C1-inhibitor deficiency from primary angioedema.

### I3 Prodromes of HAE: scientific evidence or delusional perception?

#### Avner Reshef^1,*^, Iris Leibovich-Nassi^1,2^, Hava Golander^2^

##### ^1^Barzilai University Medical Center, Ashkelon, Israel; ^2^Department of Nursing, Sackler School of Medicine, Tel Aviv University, Israel

###### **Correspondence:** Avner Reshef (avnerre@netvision.net.il)

*Allergy, Asthma & Clinical Immunology* 2019, **15(Suppl 4):**I3

Signs and symptoms (Prodromes) which antedate swelling attacks have been noted since early descriptions of Hereditary Angioedema (HAE). Regrettably, no consensual definition of a prodrome exists, contributing to ambiguity and disagreement among medical disciplines and researchers. Heralding signals occurs in other diseases, mostly characterized by a chronic and undulating course. For instance, in Schizophrenia, bipolar disorders, and some neurodegenerative diseases, early signs of abnormal behavior, occurring few years before a psychotic crisis, are regarded as a prodrome. In Migraine, a prodromal stage with subtle symptoms precedes the attacks. Early emotional changes, physical symptoms and visual ‘Aura’, are regarded as premonitory and predict oncoming paroxysm of headaches. In Herpes Zoster, a prodrome occurs 48–72 h before the typical vesicular rash, and intense pain in the involved dermatome precedes the rash in more than 90% of cases. Prodromes have also been described in Familial Mediterranean Fever (FMF) and Capillary-leak Syndrome (Clarckson’s syndrome).

A renewed interest in prodromes preceding HAE took place a decade ago, followed by an extensive literature search and some preliminary explorations. Further studies and expert opinions affirmed the fact that prodromes are more frequent than realized. Our group has recently designed and analyzed a new instrument to evaluate HAE prodromes. In this study 84% of the patients reported ever having a prodrome, and 87% said that they could predict an oncoming attack by experiencing a prodrome. A significant correlation was found between the perception of prodrome and ability to predict an oncoming attack. This data corroborates other studies reporting similarly high rate of association.

Despite consistent patient reports, prodrome’s remains elusive, and their precise nature and mechanisms are unknown. It could be hypothesized that they represent an early surge of angioedema mediators, such as complement fragments or factor XII-dependent contact system kinins. Indeed, *bradykinin* was detected in the stromal and endothelial cells of prodromal Erythema Marginatum skin rash. Additionally, evidence of early activation of the kallikerin-kinin system, with high *kininogenase* activity, *proenzyme* consumption, *high*-*molecular weight kininogen* cleavage and *C1*-*INH* function alteration, occurring before visible angioedema, was recently demonstrated. In another study, *C4* depletion and higher than baseline *C4a* levels, were detected hours before the onset of an attack.

In summary, albeit frequently reported, prodromes have not been adequately investigated, and systematic tools for their evaluation are missing. Accurate prodrome evaluation is critical for early diagnosis of attacks and timing of medical interventions, particularly in an era when effective drugs are available for self-treatment.

## Oral lectures

### O1 Parallel comparison of three different assay methodologies for measuring functional C1-inhibitor in HAE plasma

#### Zhiwei Zhou, Archana Kapoor, Yi Wang, Peng Lu, Moshe Vardi, Priya Chockalingam^*^

##### Shire (now part of Takeda), Cambridge, MA, United States

###### **Correspondence:** Priya Chockalingam (priya.chockalingam@takeda.com)

*Allergy, Asthma & Clinical Immunology* 2019, **15(Suppl 4):**O1

**Objective:** We validated three methods for measuring Functional C1-Esterase Inhibitor (fC1-INH) in K3EDTA human plasma for Shire clinical studies: conventional chromogenic assay measuring residual C1-esterase activity, Complement C1s component (C1s) binding based Electrochemiluminescent (ECL) assay, and Factor XIIa (FXIIa) binding based Enzyme linked immunosorbent assay (ELISA). We performed a side-by-side comparison of all three methods with consented pharmacodynamic (PD) samples from the SAHARA Phase 3, randomized, double-blind, placebo-controlled, two-period, three-sequence, partial crossover study that evaluated the efficacy and safety of subcutaneous administration of 2000 IU of C1 Esterase Inhibitor [Human] Liquid for Injection for the Prevention of Angioedema Attacks in Adolescents and Adults with Hereditary Angioedema (HAE).

**Methods and materials:** The validation assessments for the assays successfully fulfilled requirements for critical parameters including accuracy, precision, range of quantitation, selectivity, dilution linearity, hook effect, ruggedness and robustness, and analyte stability. The crossover SAHARA study had two treatment periods (TP) 1 and 2 of 14 weeks each; the patients were randomized into three of the treatment groups A/B, B/A and A/A for the two periods with A being 200 IU C1-INH and B being Placebo. The seventeen PD time-points were 1a, 8a, 16a, 24a, 27/28a, 27/28a 48 h (in TP1) 1b, 8b, 16b, 24b, 28b, 28b 24 h, 28b 48 h, 28b 72 h, 28b 96r (in TP2), 1 week Post and 1 month Post. The PD samples collected from 15 consented patients (out of total 75) were used in this assessment.

**Results:** Both ELISAs offered a better dynamic range of detection compared to the choromogenic method. A significant correlation was observed between the results from three fC1-INH methods when 219 individual PD data points were correlated; the Pearson ‘R’ was 0.94, 0.92, and 0.89 for C1s binding *vs.* Chromogenic, FXIIa binding *vs.* Chromogenic, and C1s binding *vs.* FXIIa binding methods, respectively. When the fC1-INH PD profiles from three methods of these 15 patients who underwent different treatment regimen in the cross over study were superimposed, the profiles matched for individual patients with an average CV (Coefficient of Variation) of 25% across time-points.

**Conclusion:** In summary, the results generated in parallel from HAE plasma using three different fC1-INH methods are comparable in spite of various principles followed in each of the assays. Since the ELISA results showed a significant correlation to the results from the conventional method, the in-house built ELISAs could serve as good alternates for measuring fC1-INH in HAE plasma with a better dynamic range of detection.

### O2 Assessment of C1-INH function – different methods, different results

#### Peter J. Späth^1,*^, Brunello Wüthirch^2^

##### ^1^Institute of Pharmacology, University of Bern, Bern, Switzerland; ^2^Allergy Unit, University of Zurich, Zurich, Switzerland

###### **Correspondence:** Peter J. Späth (peter.spaeth@pki.unibe.ch)

*Allergy, Asthma & Clinical Immunology* 2019, **15(Suppl 4):**O2

**Background:** We first reported in C1-INH-HAE type I patients under various treatment regimens, including replacement therapy, a C1-INH concentration > 40% (100% = 0.196 g/L) being associated with normalization of C1-INH function, close to normal C4 concentration and oedematous attacks becoming rare. The COMPACT study reported at C1-INH function > 40% the drastic drop in frequency of oedematous attacks.

**Aim:** Solving the discordance between the two observations.

**Materials and methods:** One or several of four different methods of C1-INH function assays were applied to 766 samples from 110 patients with HAE-C1-INH type I: two with read-outs by complex formation of C1-INH with its target proteases C1r (A1) or C1s (A2) and two with read-outs using chromogenic substrates CH_3_CO-R (B1) or C_2_H_5_CO-R (B2, R = Lys(ε-Cbo)-Gly-Arg-pNA). Here we compare results of functional assays at C1-INH concentrations ≤ 60% (assessed by RID).

**Results:** The COMPACT study assessed functional C1-INH by B1 with normal ranges (NR) of 70–130%. Linear regression revealed a function of 33% and 51% at concentrations of 40% and 60%, respectively, values that are far below the NR and that hardly are explaining rise in C4 and the clinical improvement of patients. A1 is an in-house assay and its performance can be questioned. A2 is the only FDA registered functional assay for C1-INH. The results of this test and the measured C4 values supported the observations made with the in-house test: linear regression revealed at C1-INH concentration of 40% and 60% apparent C1-INH functions of 54 and 78% (NR: 68–140%) and C4 concentrations of 0.13 and 0.17 g/L (NR: 0.11–0.26 g/L), respectively.

**Conclusions:** The fundamentally different read-out systems of assay methods provide different result in C1-INH-HAE patients and this comes apparent when C1-INH functions are compared on basis of concentrations. The results of the complex formation read-out fit better to C4 concentrations and the clinical observations. The assay systems compared are those of daily routine diagnostic testing and are based on complement parameters. However, HAE is a pathophysiology of contact activation and the kinin system. Therefore, and because of the introduction of new therapy options, it is an urgent diagnostic need to develop new routine assay methods. Assays on basis of complex formation between C1-INH and target proteases should get particular consideration.

### O3 Identification and characterization of large deletions in the *SERPING1* gene

#### Carine El Sissy^1^, Erwan Turquier^2^, Jerome Laurent^2^, Alain Sobel^3^, Laurence Weiss^2^, Veronique Frémeaux-Bacchi^1,*^

##### ^1^Assistance Publique – Hôpitaux de Paris, Laboratoire d’Immunologie, Hôpital Européen Georges-Pompidou, Paris, France; ^2^Assistance Publique – Hôpitaux de Paris, Service d’Immunologie Clinique, Hôpital Européen Georges-Pompidou, Paris, France; ^3^Assistance Publique – Hôpitaux de Paris, Centre de diagnostic, Hôpital Hôtel Dieu, Paris, France

###### **Correspondence:** Veronique Frémeaux-Bacchi (veronique.fremeaux-bacchi@aphp.fr)

*Allergy, Asthma & Clinical Immunology* 2019, **15(Suppl 4):**O3

**Background:** Hereditary angioedema (HAE) is a rare genetic disorder characterized by relapsing, on pruritic swelling of skin and submucosal tissue. HAE is mainly caused by mutation in the *SERPING1* gene, resulting in a C1-inhibitor (C1-INH) deficit. Point mutations or small deletions are responsible for the majority of cases. Most of these mutations are private, underlining the high mutation rate for this particular gene. *SERPING1* intronic regions have 17 sequences of repeating elements (*Alu*) which may represent hotspots for non-homologous recombination events, making the gene prone to deletions, insertions and duplications. In approximately 10% of the cases, HAE with C1-INH deficiency is caused by large gene rearrangement not detected by Sanger sequencing. To date, there are few reports with a detailed characterization of the large deletion breakpoints in the *SERPING1* gene.The aim of this study was to describe the molecular mechanism leading of these genetic abnormalities.

**Materials and methods:** C1-INH deficiency was diagnosed in 228 patients from 200 families in the Complement Laboratory, Hôpital Georges Pompidou, Paris, between 1995 and 2018. Genetic screening was performed by amplification by polymerase chain reaction (PCR) of all exons and flanking splices sites of the *SERPING1* gene. When no variant was detected by Sanger sequencing, Multiplex Ligation-dependent Probe Amplification (MPLA) was performed.

**Results:** MLPA has permitted to reveal the molecular mechanisms of the deficiencies in 16 unrelated patients. Partial and total *SERPING1* deletions involving one to 6 exons were identified. 49 patients were identified within the 16 families with a mean age at onset of symptoms of 10.7 years, 32% of patients had experienced laryngeal oedema, 82% abdominal crisis and 75% were receiving a long-term prophylactic treatment. The most frequent large deletion observed in the cohort was the exon 4 deletion, found in 6 independent families. Breakpoints were characterized in 5 cases. There were a 2.2 kb and 3.2 kb deletions including the exon 4 which were detected in 2 unrelated alleles. About 8.6 kb and 14 kb of genomic DNA were missing in one case of the exons 4 to 6 deletions and exons 1 to 6 deletions respectively. Altogether, the 16 large deletions presented account for 8% of all *SERPING1* mutant alleles investigated in French patients.

**Conclusions:** In summary, our study highlighted the heterogeneity of the large deletion in the *SERPING1* gene associated with C1-inhibitor deficiency.

### O4 Diagnosis of bradykinin-mediated angioedema in the emergency department: usefulness of the early biological workup

#### Samuel Luyasu^1,2,*^, Marc Simon^1^, Arije Ghannam^3^

##### ^1^Adult Emergency Department, Centre Hospitalier de Luxembourg, Luxembourg; ^2^Immuno-Allergology Department, Centre Hospitalier de Luxembourg, Luxembourg; ^3^KininX SAS, Grenoble, France

###### **Correspondence:** Samuel Luyasu (sluyasu@yahoo.fr)

*Allergy, Asthma & Clinical Immunology* 2019, **15(Suppl 4):**O4

**Background:** Bradykinin-mediated angioedema (BK-AE) refers to acute and often recurrent swelling of subcutaneous and/or submucosal areas. This condition is typically caused by C1-inhibitor (C1Inh) deficiency or low bradykinin catabolism e.g. under angiotensin-converting enzyme (ACE) inhibitor treatment. Angioedema with normal C1Inh (AE-nC1Inh) is also related to high plasma bradykinin and is assumed to be very rare^1^. We previously showed that activation of the kallikrein-kinin system occurs at the very early phase of BK-AE (i.e. during prodromes)^2^. Therefore, we sought to verify if the biological workup performed at admission in the emergency department could help to improve the diagnosis.

**Materials and methods:** We retrospectively analyzed all patients admitted during 12 months in our adult emergency department for acute angioedema without evidence of anaphylaxis. Plasma samples were analyzed for C1Inh function, spontaneous kallikrein activity and kinin catabolism. All patients have been examined by an allergologist.

**Results:** Twenty-six patients were admitted among which 4 were excluded for other final diagnosis. The remaining patients were 11 males and 11 females, aged 24–95 years (mean 46.6 years). It should be noted that one patient suffered from AE-nC1Inh for more than 20 years before the diagnosis was made. All patients had normal C1Inh function. Increased kallikrein activity was observed in 9 (41%) patients (9.9–44.7 nmol/min/ml; reference < 10.6 nmol/min/ml females, < 9.2 nmol/min/ml males). Low activity of one or more kininases was seen in 18 (82%) patients. The most prevalent kininase deficit was ACE (16 patients), followed by aminopeptidase P (5 patients), carboxypeptidase N (3 patients) and dipeptidylpeptidase IV (2 patients). Multiple kininases deficits were observed in 6 patients (4 with two deficits, 2 with three deficits). ACE inhibitors were taken by 4 patients, one of them having no detectable biological abnormality. No patient took gliptin treatment. An association between high plasma kallikrein activity and one or more kininases deficits was seen in 7 (32%) patients. C1Inh concentrate or Icatibant was administered to 13 patients with rapid clinical improvement. Kallikrein activity was analyzed before and 2 h after C1Inh treatment in 5 patients. This showed high initial kininogenase activity in 4/5 patients with a 58%–86% reduction thereafter.

**Conclusions:** Our experience shows that C1Inh deficiency is not the main cause of BK-AE, high kininogenase activity and/or low kinin catabolism seeming to account for most cases. By performing a biological workup during the acute phase, the diagnosis performance could be enhanced. Knowing that angioedema may lead to life-threatening situations, this issue is essential to improve patient care.

**References**

1. Zuraw BL. Hereditary angioedema with normal C1-inhibitor: Four type and counting. J Allergy Clin Immunol. 2018;141(3):884-885.
2. Luyasu S, Charignon D, Ponard D, Drouet C, Ghannam A. Angioedema: Systemic activation process during prodromes. Ann Allergy Asthma Immunol. 2018;121(2):224-249

### O5 SGP 120 and contact system in hereditary angioedema. Diagnostic tool in HAE with normal C1-inhibitor?

#### Blas Larrauri^1,2^, C. Garren Hester^2^, Haixiang Jiang^2^, Vojislav D. Miletic^2^, Alejandro Malbran^1^, Konrad Bork^3^, Allen Kaplan^4,*^, Michael Frank^2^

##### ^1^Unidad de Alergia, Asma e Inmunología Clínica, Buenos Aires, Argentina; ^2^Department of Pediatrics, Duke University Medical Center, Durham, North Carolina, United States; ^3^Department of Dermatology, Johannes Gutenberg University, Mainz, Germany; ^4^Department of Medicine, Medical University of South Carolina, Charleston, United States

###### **Correspondence:** Allen Kaplan (kaplana@musc.edu)

*Allergy, Asthma & Clinical Immunology* 2019, **15(Suppl 4):**O5

Mutations of C1inh are present in some patients with hereditary angioedema with normal C1-inhibitor (HAE-nl-C1inh), but the underlying disease mechanism remains unclear. There is no accepted biomarker for this disease.

In 1989 while developing a new purification procedure for the human complement protein C2^1^ we isolated a then unidentified plasma protein with a molecular weight of 120 Kd. It appeared related to the contact system of coagulation because incubating plasma at 4 °C in glass tubes led to cleavage of the protein. Plasma deficient in high-molecular-weight kininogen (HMWK) and kallikrein when treated similarly did not show cleavage of the protein. If a mixture of HMWK and prekallikrein was added to deficient plasma, the glycoprotein was cleaved after contact activation^2^. In 1994, Xiao Ping Pu and colleagues isolated a similar protein from plasma^3^. Using a partial cDNA sequence, they suggested that their protein had closely related sequence homologies to the heavy chains of the inter-α-trypsin inhibitor (ITI) superfamily^4^. This group of 6 separate proteinase inhibitors is characterized by the presence of a common light chain, and is susceptible to proteolysis by the enzymes they inhibit. ITI heavy chain 4 (ITIH4) is different from the other members of the group of ITI proteins because it is the only member that has no light chain and circulates as a free isoform. ITIH4 is also the only kallikrein sensitive protein among this group and has a molecular weight of 120 KD. We isolated our protein and identified it as IATI heavy chain 4. We examined fragmentation of the protein in plasma from normal, patients with HAE types 1 and 2 and patients with HAE-nl-C1inh. ITIH4 is fragmented in almost all patients with HAE types 1 and 2 and is intact in almost all normals and patients with HAE-nl-C1inh. On incubation of patient plasma in plastic tubes at 4 °C the protein is intact in normal and cleaved in plasma of patients with HAE-nl-C1inh. We studied C1inh in the same samples. C1inh was at low levels or was cleaved in HAE type 1 or 2 disease. It was normal structurally and functionally in normal and patients with HAE-nl-C1inh. However on incubation in plastic at 4 °C the C1inh was cleaved and lost function in patients with HAE-nl-C1inh but not in normals. Antiserum to this protein is commercially available and we propose that study of its cleavage pattern provides a new means for the laboratory diagnosis of HAE-nl-C1inh.

**References**

1. Hammer, C.H., Jacobs, R.M. and Frank, M.M. Isolation and characterization of a novel plasma protein which binds to activated C4 of the classical complement pathway. J. Biol. Chem. 264(4):2283-2291, 1989.
2. Langlois PF, Pilatte Y, Basta M, Fries LF, Frank MM, and Hammer CH; A newly identified plasma protein, sgp 120, is cleaved after activation of the kinin-generatin pathway. Complement Inflamm 1989; 6: 359. (Abstr. 138).
3. Pu XP, Iwamoto A, Nishimura H, Nagasawa S. Purification and characterization of a novel substrate for plasma kallikrein (PK120) in human plasma. Biochim Biophys Acta. 1994 19;1208:338-43.
4. Hitoshi Nishimura, Ikuko Kakizaki, Tatsushi Muta, et al. cDNA and deduced amino acid sequence of human PK120, a plasma kallikrein-sensitive glycoprotein FEBS Letters 1995 357; 207-211.

### O6 Changes of complement parameters during erythema marginatum in patients with hereditary angioedema

#### Kinga Viktória Kőhalmi^1,*^, Anna-Lise Ferrara^2^, Blanka Mező^3^, Nóra Veszeli^3^, Ágnes Holdonner^1^, Milos Jesenak^4^, Lilian Varga^1^, Henriette Farkas^1^

##### ^1^Hungarian Angioedema Reference Center, 3^rd^ Department of Internal Medicine, Semmelweis University, Budapest, Hungary; ^2^University of Naples Federico II, Department of Translational Medical Sciences, Naples, Italy; ^3^MTA-SE Research Group of Immunology and Hematology, Hungarian Academy of Sciences and Semmelweis University, Budapest, Hungary; ^4^Department of Pediatrics, Martin University Hospital, Martin, Slovakia

###### **Correspondence:** Kinga Viktória Kőhalmi (kinga.viktoria.kohalmi@gmail.com)

*Allergy, Asthma & Clinical Immunology* 2019, **15(Suppl 4):**O6

**Background:** Hereditary angioedema caused by deficiency of the C1-inhibitor protein of the complement system (C1-INH-HAE) is characterized by recurrent episodes of subcutaneous/submucosal edema which may be preceded by erythema marginatum (EM). Our aim was to understand the pathomechanism of EM symptoms by analyzing the parameters of the complement system.

**Materials and methods:** Eight C1-INH-HAE patients (1 man, 7 women, median age: 45.3 years), followed-up in Angioedema Reference Centers were investigated. These patients experienced EM on several occasions during their lifetime and blood samples were obtained during EM in all cases. The clinical characteristics of EM’s were recorded by the *Erythema Marginatum Detailed Questionnaire*. In the sera taken from symptom-free patients, during EM and during HAE attack periods, the following complement parameters were measured: C1q, C3, C4, C1-INH, Factor I, Factor B, Factor H, anti-Factor H, anti-C1q, and anti-C1-INH (IgG, IgA, IgM) antibodies. Total activity of the classical-, lectin- and alternative complement pathways and the activity of the C1-INH were investigated as well. Measurements were completed of complement activation products (C3a, C4a, C4d, C5a and sC5b-9) in EDTA plasma. All subjects consented to the study.

**Results:** We observed the following differences between samples taken during HAE attack vs. during EM: C3 (p = 0.0047), C4 (p = 0.0313), C1-INH concentration (p = 0.003), Factor B (p = 0.0391), and the activation product sC5b-9 (p = 0.0234) levels were significantly lower during HAE attack. Differences in samples during EM vs. symptom-free period were as follows: C3 (p = 0.049), C4 (p = 0.015), Factor B (p = 0.0084) proteins and C4a (p = 0.0114) activation product levels were significantly lower during EM. Levels of sC5b-9 were lower during EM compared to the symptom-free period, however it wasn’t significant (p > 0.05) and further decrease were observed during HAE attack in sC5b-9 (p = 0.049) and C4d levels (p = 0.044).

**Conclusions:** According to our investigations, EM can be considered as the first phase of the HAE attack, as levels of C3, Factor B, C1-INH concentrations and C4 begin to decrease during the prodromal symptom, and this trend continued during HAE attacks. Nevertheless, more patients and further investigations of the kinin-kallikrein, coagulation and fibrinolytic systems are needed for better understanding of the pathomechanism of EM. A new, individualized therapy, administered during EM to prevent the development of HAE attacks seems to be thoroughly grounded.

This study was supported by OTKA K124557 and the Pharming Group NV.

### O7 Clinical and genetic characteristics of patients with hereditary angioedema at a large tertiary care hospital in Saudi Arabia

#### Farrukh Sheikh^1,*^, Huda Alajlan^2^, Saad Alshareef^1^, Maram AlBanyan^1^, Rand Arnaout^1,3^, Hamoud AlMousa^3^, Anas M. Alazami^2^

##### ^1^Section of Allergy & Immunology, Department of Medicine, King Faisal Specialist Hospital & Research Centre, Riyadh, Saudi Arabia; ^2^Department of Genetics, King Faisal Specialist Hospital & Research Centre, Riyadh, Saudi Arabia; ^3^Section of Allery & Immunology, Department of Pediatrics, King Faisal Specialist Hospital & Research Centre, Riyadh, Saudi Arabia

###### **Correspondence:** Farrukh Sheikh (fsheikh96@kfshrc.edu.sa)

*Allergy, Asthma & Clinical Immunology* 2019, **15(Suppl 4):**O7

**Background:** Hereditary Angioedema (HAE) is a rare autosomal dominant disorder characterized by potentially fatal swelling of the larynx. In addition, abdominal angioedema can lead to misdiagnosis and needless surgical intervention, along with narcotic dependence. The prevalence of HAE is estimated at roughly 1:50,000 individuals, however, the overall epidemiological data for this disorder remains scarce and inadequate. The main gene linked to HAE pathogenesis is *SERPING1*, the gene that encodes for C1-INH. Less commonly observed are mutations in *F12*, the gene that encodes the Coagulation Factor XII (Hageman Factor). Very recently two other genes have also been described: *PLG* and *ANGPT1*

**Objectives:** We sought to examine the clinical features as well as the molecular genetic defects observed in HAE in the Saudi population.

**Methods:** Thirty-six (36) patients with a diagnosis of HAE, who were being followed at the Immunology clinics at King Faisal Specialist Hospital (both Riyadh and Jeddah) were included in the study after informed consent. This study is RAC approved. (RAC # 2080 025)

**Results:** HAE diagnosis was based on the classic clinical presentation of angioedema without urticaria as well as characteristic lab features. 38.8% of the patients were males and the mean age was 31.08 years.

Initial molecular screening was performed for the *SERPING1* gene. For cases that were negative for this gene, primers were then designed against all coding regions of F12, PLG and ANGPT1 for PCR analysis.

Twenty-six (26) patients were found to have mutations in *SERPING1* gene, of which the most common was NM_000062:c.1397G>A:p.R466H (Table 1). In addition, one novel mutation (NM_000062:c.1202T>A:I401N) was uncovered in a single Saudi family. All negative cases have been screened for *F12*, *PLG* and *ANGPT1* gene mutations, but thus far, no such mutations have been found.

**Table 1:** Breakdown of mutations found in the HAE cohort

Number of patients	Observed mutation
14	SERPING1:EX8.1:NM_000062:c.1397G>A:p.R466H
3	NM_000062:c.1305delT:p.L436Ffs*14
3	NM_000062:c.1202T>A:p. I401 N
2	NM_000062:c.1029 + 1G>A
1	SERPING1:NM000062:EX3-1:c.265C>T:p.Q89*
1	NM_000062.1:c.1143delC:p.I382Sfs*15
1	SERPING1:NM_000062:EX7:c.1187_1188insT:p.T397Nfs*28
1	SERPING1:EX3.1:NM_000062: c.373G>T:p.G125*
10	Unsolved
36	Total Patients

**Conclusions:** Molecular analysis of this HAE cohort has revealed consistent autosomal dominant inheritance and the presence of *SERPING1* mutations in > 50% of patients. Although some mutations have been found across multiple families, R466H is the only mutation which appears to dominate the HAE genetic landscape in the region. The lack of observed mutations in *F12, PLG* and *ANGPT1* suggest that there are no prevalent founder mutations in these genes in the local population. Unsolved pedigrees are being prioritized for whole exome sequencing.

### O8 D-Dimer and C-Reactive protein in urticaria and angioedema at the Emergency Room

#### Riccardo Senter, Stela Dako, Alessandra Pizziol, Mauro Cancian

##### Department of Medicine, University of Padua, Padua, Italy

###### **Correspondence:** Riccardo Senter (mcancian@unipd.it)

*Allergy, Asthma & Clinical Immunology* 2019, **15(Suppl 4):**O8

**Background:** D-Dimer (D–D) and C-Reactive Protein (CRP) have been reported to be increased and correlate with severity of symptoms in urticaria, which is histamine-mediated and may occur with or without angioedema (AE), as well as in histaminergic or bradikynin-induced, primary AE [1–3]. These findings may elicit the suspicion of inflammatory pattern and deep venous thrombosis (DVT) at the Emergency Room (ER), thus prompting to unnecessary and harmful procedures.

**Objectives:** To assess the prevalence of high D-Dimer and CRP levels in patients with acute urticaria and/or AE and to investigate the possible presence of DVT in subjects with increased plasma D–D concentrations.

**Materials and methods:** Thirty-five patients (10 male, 25 female; age: mean = 45.51 ± 17.65 years SD, range 15–80 years) admitted to the ER of Padua University Hospital for urticaria/angioedema (urticaria without AE 19 patients, urticaria with AE 7 patients) or primary AE (9 patients) underwent blood sampling for D-dimer, CRP and other factors related to the coagulation pathway. In all the subjects with D–D values higher than normal (referral range of Padua Hospital Laboratory: 0–250 μg/L), a lower limbs venous ultrasonography was performed and these patients were re-evaluated after 72–96 h from discharge for clinical and biochemical assessment.

**Results:** The overall mean value of plasma D-dimer was 623.65 ± 945.88 µg/L SD (range: 50–4210 µg/L) with 15/35 cases (43%) showing increased levels ($\overline{\text{x}}$ = 1316.86 ± 1126.18 µg/L SD; range 319–4210 µg/L). A strong relationship (p = < 0.001, r = 0.70) between D–D end CRP fluctuations was also observed (not shown), with both parameters decreasing after 72–96 h of combined therapy (antihistamines plus steroids) and in parallel with symptom remission (Fig. 1). No patients presented DVT, regardless of D–D levels.

**Fig. 1:**
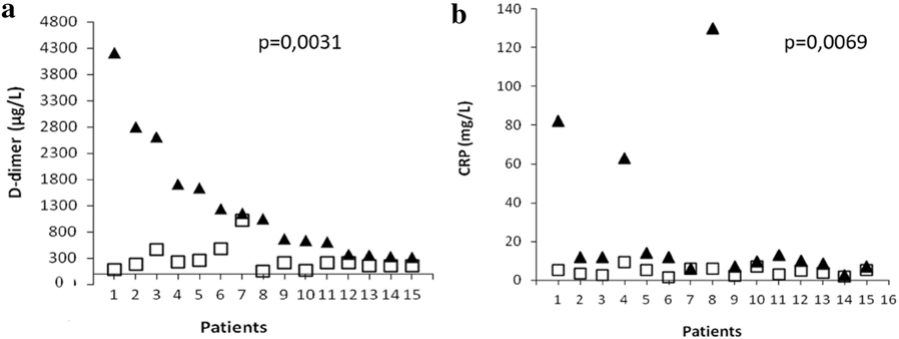
Comparison between D-dimer (**a**) and CRP values (**b**) on ER admission (▲) and after 72–96 h (□) of therapy in the 15 patients who underwent the follow up visit (Patient n°7 was the only one not showing a clear remission of symptoms at follow up)

**Conclusions:** Increased levels of CRP and D-Dimer, probably rising from extravascular activation of the coaugulation pathway rather than from the vascular district, may be frequently detected on acute attacks of urticaria and/or angioedema. Although both the biomarkers seem well correlate with disease activity, they are not predictive of systemic phlogosis and thromboembolism. As CRP and D–D do not affect therapeutic strategies in urticaria and AE, these tests should not be included among ER screening analyses but in cases of strong pretest probability for inflammation and thromboembolism.

The study was approved by the local Ethical Committee (protocol number 4314/AO/17)

**References**

1. Asero R, Tedeschi A et al. Severe chronic urticaria is associated with elevated plasma levels of D-dimer. Allergy 2008; 63:176-80
2. Cugno M, Zanichelli A et al. Plasma biomarkers of acute attacks in patients with angioedema due to C1inhibitory deficiency. Allergy 2009; 64:254-7
3. Kolkhir P, André F et al. Potential blood biomarkers in chronic spontaneous urticaria. Clin Exp Allergy 2017; 47:19-36

### O09 Validity and reliability of a new instrument for the evaluation of HAE prodromes

#### Iris Leibovich-Nassi^1,2,*^, Hava Golander^1^, Raz Somech^3^, Dov Har-Even^4^, Avner Reshef^2^

##### ^1^Department of Nursing, Sackler School of Medicine, Tel Aviv University, Tel Aviv, Israel; ^2^Barzilai University Medical Center, Ashkelon, Israel; ^3^Safra Pediatric Medical Center, Sheba Medical Center, Ramat Gan, Israel; ^4^Bar-Ilan University, Ramat Gan, Israel

###### **Correspondence:** Iris Leibovich-Nassi (irisl@bmc.gov.il)

*Allergy, Asthma & Clinical Immunology* 2019, **15(Suppl 4):**O9

**Background:** Many Hereditary Angioedema (HAE) patients describe premonitory signs and symptoms (Prodromes), which antedate the attacks. Although frequently reported, prodromes have not been adequately investigated, due to lack of systematic tools. HAE prodromes should be better defined and needs a dependable instrument as a Patient Reported Outcome (PRO) metric.

**Methods:** First phase of the study consisted of personal and health data acquired from HAE patients. Out of 233 patients, 197 (84.5%) responded to a preliminary questionnaire, inquiring if they ever had a prodrome, and if they can predict an oncoming attack by having a prodrome. Mean age was 36.7 ± 20.3 years (Y, range: 2–80, females 42.5%). Mean age of onset was 10.7 ± 10.3Y, age of diagnosis was 15.5 ± 15.1Y. Nearly 62% of this group were diagnosed by age 10 (M > F, P < 0.05). Preliminary questionnaire was constructed, based on the literature and investigator’s experience. Six new instruments were used to reach a robust evaluation scale. Data from the preliminary interviews was used to test the instrument’s construct and internal validity. In the 2^nd^ phase, patients were interviewed to obtain data on prodromes and attacks. After final refinement of the instrument, a retrospective and prospective data were obtained and statistical analysis was performed to study prodromes and attacks associations.

**Results:** In the 1st phase, 165/197 (84%) reported ever having a prodrome, 143/165 (87%) could predict an oncoming attack by experiencing a prodrome. There was a significant correlation between the perception of prodrome and ability to predict an oncoming attack (p < 0.01, r = .79). The Internal validity of the new questionnaire, constructed based on the preliminary study, was high, as well as its internal reliability (Cronbach’s α = .70 to .96). Prodromes and attacks, analyzed for each body system cluster, were highly correlated (r = .33, p < .01). In the 2^nd^ phase, 66 patients (33.5%) completed a questionnaire that covers ‘clusters’ of body systems, affected by both events. Differences between the clinical dimensions of prodromes (i.e. pain, severity, impairment, dysfunction, duration), were evaluated by one-way MANOVA with repeated measurements, and shown to be segregated from attacks on all dimensions [F (4, 56) = 45.7, P < .001, Eta^2^ = .77]

**Conclusions:** A questionnaire-based PRO instrument for the evaluation of HAE prodromes and attacks was designed and tested. The high internal validity and reliability makes it useful for studying clinical expressions of HAE. A prodrome/attack evaluation instrument can be useful in the diagnosis of oncoming attacks and timing of medical interventions.

### O10 Psychosocial burden of hereditary angioedema in a Canadian cohort

#### Julia Hews-Girard^1,2,*^, M. Dawn Goodyear^1,2^

##### ^1^Southern Alberta Rare Blood and Bleeding Disorders Comprehensive Care Program, Calgary, Alberta, Canada; ^2^Cumming School of Medicine, University of Calgary, Calgary, Alberta, Canada

###### **Correspondence:** Julia Hews-Girard (julia.hewsgirard@ahs.ca)

*Allergy, Asthma & Clinical Immunology* 2019, **15(Suppl 4):**O10

**Background:** Patients with chronic diseases have increased levels of anxiety, depression and stress – all of which may affect quality of life (QoL) [1, 2]. The unpredictability of swelling in hereditary angioedema (HAE) results in significant anxiety, depression and impaired QoL [3]. The availability of HAE-specific therapies and use of prophylaxis has been associated with an improvement in QoL; however, literature discussing specific aspects of the psychosocial burden of HAE is lacking.

**Aim**

1. To assess prevalence of health-related (HR) depression, anxiety and stress
2. To evaluate health-related QoL (HRQoL)

**Methods:** Seventeen patients, ≥ 18 years, with a confirmed diagnosis of HAE type 1 or 2 (Table 1), completed the following self-reported questionnaires: SF36v2 (generic HRQoL), AE-QoL (disease-specific HRQoL) and both the Depression, Anxiety, Stress Scale (DASS) and the Diagnostic and Statistical Manual of Mental Disorders (DSM5) cross cutting measures (identify/differentiate between HR depression, stress, anxiety and related symptoms) [4–7]. This study was approved by the University of Calgary Ethics Board (REB# 17-0542).

**Table 1:** Sample characteristics

Sample (n)	17
Gender (n, %)	
Male	4 (24%)
Female	13 (76%)
Age, years (mean, range)	43 (20–63)
Diagnosis (n, %)	
Type 1	11 (64.7%)
Type 2	6 (35.3%)
Treatment protocol (n, %)	
Prophylaxis	11 (64.7%)
On-Demand	6. (35.3%)
Treatment administration (n, %)	
Self-administered treatment	16 (94.1%)
Provider administered treatment	1 (5.9%)

**Results:** 100% (17/17) of participants reported increased levels of HR fatigue and fear, as well as decreased overall HRQoL. The majority of respondents reported decreased mood (88%) and increased anger (82%) concerning their disease. Disturbed sleep was reported by 76% (13/17) of participants. Most participants reported greater than average amounts of HR stress (76%, 13/17), anxiety (76%, 13/17), and depression (70.6%, 12/17). Somatic symptoms were reported by 64% (11/17) of participants, including feeling that their illness was not taken seriously by others (Fig. 1). Mean scores on all domains of the SF36v2 were significantly lower than Canadian normative data for the entire sample (p < 0.05 for all). Social and physical function, as well as emotional and physical role scores of the SF36v2 were the most impacted (Fig. 2). Female participants tended to have lower scores than male patients in most SF36v2 domains and had significantly higher HAE-related fears (AEQoL; t(5.6) = − 2.7, p = 0.035) and significantly more HAE-related stress (DASS; t(15) = − 2.2, p = 0.04) than male patients.

**Fig. 1:**
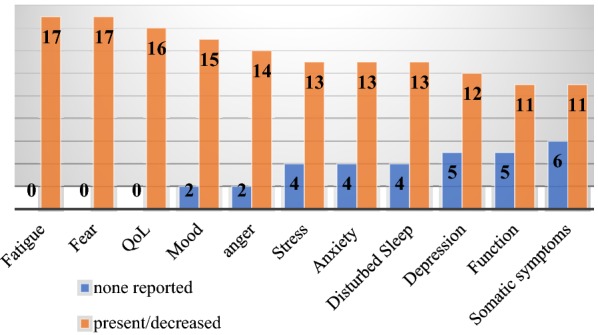
Trends in Psychosocial and HRQoL Scores

**Fig. 2:**
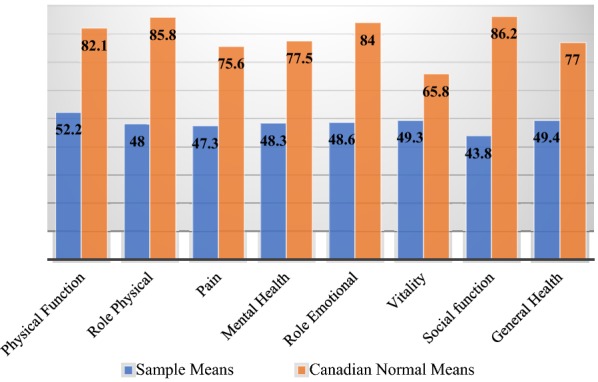
HAE cohort scores compared to Canadian normative scores (SF36v2)

**Conclusion:** This study of Canadian patients demonstrates that HAE negatively influences HRQoL as measured by the AE-QoL. More patients report depressed mood, increased stress, anxiety, anger, and sleep disturbances than not. Patients with HAE have significantly lower emotional and physical functioning than the general population; women with HAE appear to be particularly impacted. This study offers specific, valuable insight into the psychosocial burden of HAE. Future evaluation should include larger samples and further investigation of gender differences.

**References**

1. DeJean D, Giacomini M, Vanstone M, Brundisini F. Patient experiences of depression and anxiety with chronic disease. Ont Health Technol Assess Ser. 2013; 13(16):1-33
2. Jindal NL, Harniman E, Prior N, Perez-Fernandez E, Caballero T, Betschel S. Hereditary Angioedema: health-related quality of life in Canadian patients as measured by the SF36. Allergy, Asthma and Clinical Immunology. 2017; 13(4)
3. Caballero T, Aygoren-Pursun E, Bygum A, Beusterien K, et al. The humanistic burden of hereditary angioedema: results from the Burden of Illness study in Europe. Allergy, Asthma Proc. 2014; 35(1):47-53
4. Ware JE, Sherbourne CD. The MOS 36-item short-form health survey (SF36). I. Conceptual framework and item selection. Med Care 1992. 30(6):473-83
5. Weller K, Mageri M, Peveling-oberhag A et al. The Angioedema Quality of Life Questionnaire – assessment of sensitivity to change and minimal clinically important difference. Allergy. 2016; 71(8): 1203-9
6. Henry J, Crawford J. The short-form version of the depression Anxiety Stress Scales (DASS 21): Construct validity and normative data in a large non-clinical sample. Clinical Psychology. 2005; 44(2): 227-239
7. American Psychiatric Association. DSM5 Self-Rated Level 1 Cross-Cutting Symptom Measure—Adult. 2013

### O11 Angioedema and urticaria at the Emergency Room: epidemiology and clinical management in a tertiary care center in Italy during the decade 2009–2018

#### Mauro Cancian^1,*^, Giulia Mormando^1^, Alessandra Pizziol^1^, Ilaria Lazzarato^1^, Riccardo Senter^1^, Anna Chiara Frigo^2^

##### ^1^Department of Medicine, Thoracic and Vascular Sciences, University of Padua, Padua, Italy; ^2^Department of Cardiac, Thoracic and Vascular Sciences, University of Padua, Padua, Italy

###### **Correspondence:** Mauro Cancian (mcancian@unipd.it)

*Allergy, Asthma & Clinical Immunology* 2019, **15(Suppl 4):**O11

**Background:** Primary angioedema (PAE) and urticaria-angioedema syndrome (UA) are common complaints and a frequent cause of hospital admission. However, epidemiological data are still limited, with a few studies published on this topic.

**Objectives:** To assess the overall impact of PAE and UA on the Emergency Room (ER) of the General Hospital - University of Padua - Italy (GHUPD) and the fluctuations of the trend in a decade.

**Methods:** We selected 73 ICD9-CM nosologic codes potentially related to allergic symptoms. Thereafter, we read all the 12.897 discharging reports from the ER of the GHUPD associated to these codes in the decade 2009–2018, to confirm the diagnoses and collect detailed information on clinical management and outcome of each admission. All the data were then analyzed by SAS 9.2 program for Windows and statistical analyses performed by χ2 test and Fisher’s exact test. The study was approved by the local Ethical Committee (protocol number 83901/AO/16)

**Results:** 79.75% of discharging codes were consistent with the clinical features as assessed by reports’ analysis, whilst the remaining 20.25% showed to be wrong. Allergic symptoms accounted for 1.2% of the overall ER admissions (10286/859224, with an increasing trend in their prevalence (2009:1.08% → 2018:1.47%). UA was the main cause of admission (46.43%), followed by asthma (21.58%) and PAE (12.37%) (Fig. 1). Female gender was prevalent for allergy in general (58%), as well as for UA (60.86%) and PEA (53.87%), whereas women represented 49.35% of total admissions (p < 0.001 for both groups) which showed also an inverse age distribution, with a prevalence of patients older than 60 years. Foods and medicines were the most common causal factor, with use of ACE-inhibitors accounting for 18.84% of all AE cases (Fig. 2). However, no clear etiology was identified in 72.48% of UA and in 53.26% of PAE. Angioedema showed more severe clinical features than UA, with greater attribution of red/yellow codes (54.12% vs 26.79%, p < 0.001), higher prevalence of mouth involvement (42.9% vs 7.4%, p < 0.001) and more frequent epinephrine administration (3.6% vs 1.01%, p < 0.001). Moreover, AE accounted for all the 32 hospitalizations. Finally, 3 cases of death were reported in the decade, one of whom for AE involving upper airways.

**Fig. 1:**
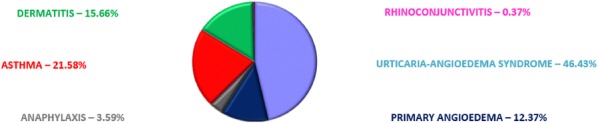
Prevalence of single diseases among all the admissions for allergic symptoms

**Fig. 2:**
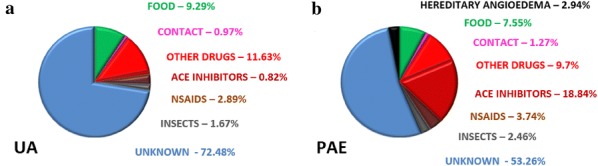
Putative etiology of urticaria-angioedema syndrome (**a**) and primary angioedema (**b**)

**Conclusions:** Our data demonstrate the significant impact of AE and UA on the ER, in terms of both patient numbers and resource use. Although a clear etiology is not detectable in most of cases, ACE-inhibitors are frequently associated with AE, which is clearly more severe than UA.

### O12 Hereditary angioedema with a specific mutation in the plasminogen gene in 18 families

#### Konrad Bork^1,*^, Karin Wulff^2^, Guenther Witzke^1^, Jochen Hardt^3^

##### ^1^Department of Dermatology, Johannes Gutenberg University, Mainz, Germany; ^2^University Medicine, Ernst Moritz Arndt University, Greifswald, Germany; ^3^Department of Medical Psychology and Medical Sociology, Johannes Gutenberg University, Mainz, Germany

###### **Correspondence:** Konrad Bork (konrad.bork@unimedizin-mainz.de)

*Allergy, Asthma & Clinical Immunology* 2019, **15(Suppl 4):**O12

Recently a new type of hereditary angioedema, hereditary angioedema with a specific plasminogen gene mutation (HAE-PLG) has been identified by whole exome sequencing and family studies. Aim of the present study was to provide more information about clinical features of HAE-PLG. A total of 18 German families with HAE-PLG were studied. In all families the missense mutation c.988A > G (p.Lys330Glu; K330E) leading to an amino acid exchange in the kringle 3 domain of plasminogen co-segregated with the clinical symptoms of HAE-PLG. None of the investigated family members had HAE symptoms but did not carry this mutation. The size of the families ranged from 2 to 15 affected family members. On average 5 members per family were affected with HAE-PLG or identified as symptom-free mutation carriers. Among 101 relatives in the 18 families there were 96 clinically affected patients and 5 symptom-free individuals with the specific mutation. Sixty-two of the 101 relatives were women (61.4%) and 39 men (38.6%). Two patients from one family had HAE-PLG combined with HAE-C1-INH and were excluded from the symptom evaluation. The remaining group comprised 94 patients who were studied for clinical symptoms of HAE-PLG. The mean age of onset of clinical signs of HAEPLG was 32.1 ± 17.7 years ranging from 4 to 74 years. The mean onset in 61/94 women was 29.2 ± 16.4 years and in 33/94 men 37.4 ± 19.0 years. Seventy-one of 94 patients (75.57%) had lip or facial swellings and 7/94 (7.4%) swellings of the extremities. Twenty-six of 94 patients (27.7%) had abdominal attacks. One patient reported on genital swellings. Sixty-nine of 94 patients (73.4%) had a total of 4.3681 tongue swellings (range 1 to 626). Thirteen of 94 patients (13.8%) had exclusively recurrent tongue swellings (range 1 to 150) and no other clinical signs. Three women died by asphyxiation due to a tongue swelling with upper airway obstruction, one woman at age 25, another one at age 36 and the third woman at age 47. The latter woman had 160 tongue swellings before the fatal upper airway obstruction. One man asphyxiated at age 75 due to a tongue swelling. Lip and facial swelling and potentially life-threatening tongue swellings are the most frequent and important symptoms of HAE-PLG.

### O13 Hereditary angioedema with C1-INH deficiency in 96 Brazilian children

#### Joanna Araújo-Simões^1^, Aline G.P. Boanova^1^, Rosemeire N. Constantino-Silva^1^, Nyla T.M.L. Fragnan^1^, Jorge A. Pinto^2^, Fernanda Minafra^2^, Rozana F. Goncalves^3^, Solange O.R. Valle^4^, Maria Luiza O. Alonso^4^, Sergio Dortas Jr.^4,19^, Ekaterini Goudouris^5^, Almerinda M, Rêgo-Silva^6^, M.M. Marques^7^, Faradiba S. Serpa^7^, Herberto Chong-Neto^8^, Nelson Rosário^8^, Eli Mansour^9^, Iramirton F. Moreira^10^, Adriana Moreno^11^, Luiza K. Arruda^11^, Persio Roxo Jr.^11^, M.P.L. Ferriani^11^, J. Jane da Silva^12^, Janaira F.S. Ferreira^13^, Pedro F. Giavina-Bianchi Jr.^14^, Priscila M. Takejima^14^, Luis Felipe C. Ensina^15^, Regis A. Campos^16^, Eliana Toledo^17^, Camila Veronez^18^, Joao B. Pesquero^18^, Sandra U.M. Palma^1^, Anete S. Grumach^1,*^

##### ^1^FMABC- Faculdade de Medicina, University Center Health ABC, Brazil; ^2^UFMG - Federal University of Minas Gerais, Brazil; ^3^Private clinic Belo Horizonte - MG, Brazil; ^4^HUCFF- Hospital Universitário Clementino Fraga Filho, Federal University of Rio de Janeiro UFRJ, Brazil; ^5^IPPMG - Instituto de Puericultura e Pediatria Martagão Gesteira, Federal University of Rio de Janeiro UFRJ, Brazil; ^6^UFPE - Federal University of Pernambuco, Brazil; ^7^EMESCAM - Santa Casa de Misericórdia de Vitória, Brazil; ^8^UFPR - Federal University of Paraná, Brazil; ^9^UNICAMP - Faculdade de Ciências Médicas, Brazil; ^10^UFAL - Federal University of Alagoas, Brazil; ^11^USP/RP - Ribeirão Preto Medical School, University of São Paulo, Brazil; ^12^HU-UFSC - Federal University of Santa Catarina, Brazil; ^13^UECE State University of Ceará/Hospital Infantil Albert Sabin, Brazil; ^14^USP/SP - University of São Paulo, Brazil; ^15^UNIFESP - Federal University of São Paulo, Brazil; ^16^UFBA - Federal University of Bahia, Hospital Universitário Prof. Edgard Santos, Brazil; ^17^FAMERP - Faculdade de Medicina de São José do Rio Preto, Brazil; ^18^Instituto de Biofisica, Federal University of Sao Paulo, Brazil; ^19^Universidade Iguaçu (UNIG), Brazil

###### **Correspondence:** Anete S. Grumach (asgrumach@gmail.com)

*Allergy, Asthma & Clinical Immunology* 2019, **15(Suppl 4):**O13

There are scarce data about Hereditary Angioedema (HAE) with C1-INH deficiency in pediatric patients. Although symptoms of HAE begin early in life, diagnosis of the disease is delayed in this period of life. In addition, several new therapies are still restricted to older ages. Considering the restricted access to diagnosis and therapy in developing countries, we evaluated clinical and laboratorial characteristics and therapy of pediatric patients from Brazilian reference centers.

**Methods:** Medical records of HAE patients aged less than 18 years old, whose diagnosis was confirmed by quantitative and/or functional C1-INH, were included. The following data were collected: age of diagnosis, age at onset of symptoms, prodromes, symptoms, triggering factors, diagnostic tests and treatment. Descriptive statistical analysis was performed. The study was approved by ethical committee.

**Results:** 96 participants (52 M:44F) from 17 reference centers on HAE were included: 50% from southeast, 23% from northeast, 16% from midwest, and 11% from south of Brazil. Family history was present in 72/96 patients. Twenty percent of the patients were asymptomatic. Age at onset of symptoms was < 1 year of age in 27%; 1–5 years in 45%; 6–10 years in 21% and 11 to  < 18 years in 7%. Median age at diagnosis was 7 years old. Prodromes were reported among 25% of the patients: local burning in 10%; serpiginous erythema in 7%; fatigue and nausea in 2% each and irritability in 5.2%. Angioedema attacks affected: face (5%); lips (13%); tongue (11%); eyelid (6%); ear (2%); neck (1%); hands (19%); arms (15%); legs (5%); genitals (7%); upper airways (3%) epigastric pain and/or abdominal pain (55%). One fourth of the patients had no trigger factor identified. Severity of attacks were mild in 32%; moderate in 40% and severe in 26%. Misdiagnosis included: allergy 16% and helminthiasis 5%. Previous ER visits were reported by 56.3%. Surgical intervention (appendectomy) prior to HAE diagnosis occured in 4%. Long term prophylaxis was introduced in 54% (53/96): tranexamic acid in 76% (40/53); danazol in 13% (7/53) and oxandrolone in 11% (6/53). Androgens were used in 4/13 under 12 years of age. Short-term prophylaxis was prescribed in 10/96 (tranexamic acid 6/10; danazol 3/10 and plasma derived C1-INH 1/10). 32/96 patients received specific treatment of acute attacks, including Icatibant to 4/32; fresh frozen plasma to 15/32; plasma derived C1-INH to 11/32 and tranexamic acid to 13/32 patients.

**Conclusions:** Although more than 80% of the patients had family history and several members affected, there was a delay in diagnosis. Abdominal pain and surgical interventions were less frequent than reported in adulthood. Attenuated androgens were prescribed for pediatric patients, probably due to restricted access to on demand therapy. Only recently Icatibant was licensed for use in children, nevertheless it had been previously used in our population. Educational programs should focus on Pediatricians, aiming at reducing delayed diagnosis and providing appropriate therapy.

### O14 Determinants of breakthrough attacks in hereditary angioedema patients undergoing dental procedures

#### Jonathan A. Bernstein^1,*^, Umesh Singh^1^, Bill Lumry^2^, Paula Busse^3^, James Wedner^4^, Aleena Banerji^5^, Tim Craig^6^, Raffi Tachdjian^7^, Henry Li^8^, Joshua Jacobs^9^, Bruce Zuraw^10^, Marc Riedl^10^, Mark Davis-Lorton^11^, Sandra Christiansen^10^, Bruce Richie^12^, the HAEA organization

##### ^1^Division of Immunology/Allergy, Department of Internal Medicine, University of Cincinnati College of Medicine, Cincinnati, OH, United States; ^2^Allergy and Asthma Research Associates Research Center, Dallas, TX, United States; ^3^Division of Clinical Immunology, Mount Sinai, New York, NY, United States; ^4^Allergy and Immunology, Washington University School of Medicine, United States; ^5^Division of Rheumatology, Department of Allergy & Immunology, Massachusetts General Hospital, Boston, MA, United States; ^6^Department of Medicine and Pediatrics, Penn State University, Hershey, PA, United States; ^7^Allergy and Immunology, UCLA Medical Center, Santa Monica, United States; ^8^Institute for Asthma and Allergy, PC, Chevy Chase, MD, United States; ^9^Allergy and Asthma Clinical Research, Inc., Walnut Creek, CA, United States; ^10^Division of Rheumatology, Allergy & Immunology, University of California, San Diego, La Jolla, CA, United States; ^11^Department of Medicine, Winthrop University Hospital, Mineola, NY, United States; ^12^Department of Medicine, Alberta Health Services, Edmonton, Canada

###### **Correspondence:** Jonathan A. Bernstein (bernstja@ucmail.uc.edu)

*Allergy, Asthma & Clinical Immunology* 2019, **15(Suppl 4):**O14

**Background:** Short-term prophylaxis (STP) in HAE is commonly administered to prevent breakthrough attacks after minor surgical trauma, such as dental procedures. This purpose of this study was to identify clinical determinants predictive of HAE breakthrough attacks after STP.

**Methods:** A cross-sectional questionnaire survey distributed to HAE subjects (n = 250) obtained information related to STP use outcomes after dental procedures. Patient demographics, comorbidities and their treatment, as well as the frequency, severity and location of their prior HAE attacks were compared between HAE groups with vs. without breakthrough attacks after STP. Analysis was performed using univariate Chi square test of association followed by multivariate generalized linear regression and decision trees after checking for multicollinearity.

**Results:** A higher proportion of HAE subjects with a history of moderate to severe dental procedure-related HAE attacks were on LTP (i.e., 78.8% vs 57.1%; p = 0.002) and received STP during dental procedures (68.2% vs. 40.8%; p = 0.0001) compared to subjects with none to mild attacks.Overall 20 out of 120 subjects being treated with STP reported breakthrough attack after dental procedures (18/20 were on LTP). C1Inh was the most commonly used STP agent being administered to 91 of 120 subjects.

Comorbid conditions including ‘hypertension’, ‘hypercholesterolemia’, ‘chronic rhinitis’ and use of ‘supplementary oxygen’, taking ‘phenytoin’ and ‘immunosuppressant’ medications as well as having ‘dentures’ were significantly associated with a ‘history of post-procedure HAE attacks’. After checking for multicollinearity in the univariate analysis, the significant factors used in the multivariate analysis were ‘history of procedural angioedema attacks’, ‘younger age (< 25.9)’, and comorbid ‘asthma’. The above comorbid conditions as well as severity of previous attacks, prodromes, frequency of routine dentist visits, avoidance of dental check-ups/cleanings or procedures because of potential HAE attacks and having dentures were not significantly associated with the occurrence of breakthrough attacks.

**Conclusion:** Young age (< 26), history of post-procedural HAE attacks and comorbid asthma are significant risk factors for breakthrough HAE attacks following dental procedures despite STP and LTP. The significant association of post-dental procedure HAE breakthrough attacks with asthma implies that HAE subjects with comorbid asthma may represent a distinct HAE phenotype which requires further investigation (Table 1).

**Table 1:** Results showing significant determinants of breakthrough attacks after short-term prophylaxis in HAE subjects after dental procedures

	Odds ratio	LCL	UCL	*p* value
Multivariate analysis				
Age	0.94	0.90	0.99	0.03
H/O procedural-angioedema (yes) vs (no)	12.62	3.42	46.53	0.0001
Asthma (yes) vs Asthma (no)	3.57	1.04	12.21	0.042
Univariate Analysis				
H/O procedural-angioedema (yes) vs (no)	8.50	2.34	30.91	0.0012
Asthma (yes) vs Asthma (no)	4.15	1.22	14.15	0.02
HTN (yes) vs Asthma (no)	9.81	3.02	31.87	0.0001
Chronic rhinitis (yes) vs Asthma (no)	8.95	2.84	28.25	0.0002
Hypercholesterolemia (yes) vs Asthma (no)	4.51	1.24	16.33	0.0219

**Note:** Use of corticosteroids, supplementary oxygen, phenytoin and immunosuppressants (i.e., medications for comorbidities) were significantly associated with breakthrough attacks but were determined to have multicollinearity with history of procedure-related angioedema and therefore, were not included in the multivariate analysis.

30% (20 of 120) subjects receiving STP had breakthrough attacks.

About 30% (17 of 57 who received STP) of those with previous history of post-procedural angioedema attacks vs. 3% of those without such history reported breakthrough attacks.

About 41% of those with history of asthma vs. 12.6% of those without such history reported breakthrough attacks (OR 4.1 [1.2, 14.1], p = 0.02) (Fig. 1).

**Fig. 1:**
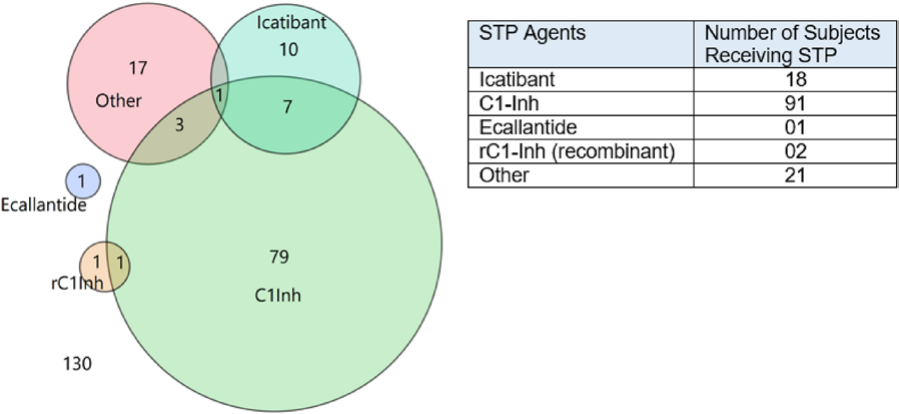
Diagram of counts of subjects reporting the specific STP agents (choices were “C1Inh”, “Icatibant”, “Ecallantide”, “rC1Inh”, “other”). Of the 120 subjects who received STP, a total of 91 subjects reported receiving C1Inh [human] as STP (which includes 79 subjects exclusively on C1Inh [human], 1 subject on rC1Inh + C1Inh, 7 on Icatibant + C1Inh, 3 on Other + C1Inh, and 1 on Other + Icatibant + C1Inh.). 10 received Icatibant only as STP, 1 received Ecallantide only, and 1 received rC1Inh only. 17 subjects reported receiving other STP agent (choices were “C1Inh. 130 subjects did not receive STP

### O15 A *SERPING1* variant that causes C1-inhibitor deficiency without hereditary angioedema

#### Silvia Berra^1^, Chiara Suffritti^1^, Andrea Zanichelli^1,2^, Debora Parolin^1^, Maddalena A. Wu^1,2^, Francesca Perego^3^, Marco Cicardi^1,2^, Sonia Caccia^1,*^

##### ^1^”L. Sacco” Department of Biomedical and Clinical Sciences, University of Milan, Milan, Italy; ^2^ASST Fatebenefratelli Sacco, Milan, Italy; ^3^Istituti Clinici Scientifici Maugeri IRCCS, Milan, Italy

###### **Correspondence:** Sonia Caccia (sonia.caccia@unimi.it)

*Allergy, Asthma & Clinical Immunology* 2019, **15(Suppl 4):**O15

More than 400 mutations (around 25% de novo) in *SERPING1* have been described to cause functional C1-inhibitor (C1-INH) deficiency and hereditary angioedema (HAE). Angioedema symptoms and C1-INH deficiency co-segregate within the families with autosomal dominant inheritance. Two promoter and two structural *SERPING1* variants escape this rule. These four mutations represent a recessive character: they cause the clinical phenotype of HAE only when present in homozygosity.

Here we describe variant g.22006G > A (p.Arg494Gln) in *SERPING1,* detected in 12 subjects from four families with C1-INH deficiency and no HAE. The variant is located in a region of the molecule that is critical for regulating SERPIN conformational state.

Index cases in each family were identified due to urticaria symptoms that prompted measurements of C1-INH and C4. In none of the four families there was evidence for inherited angioedema without wheals while inherited C1-INH and C4 deficiency were clearly present. Inhibitory function of C1-INH against C1s, kallikrein and FXIIa was below 50% of normal. Repeated measurements of complement parameters in C1-INH deficiency carriers, showed a degree of variability, which is never detected in typical C1-INH-HAE. When these subjects underwent danazol treatment, complement parameters rapidly normalized. All C1-INH deficient subjects in the four families, carried the mutation g.22006G > A (p.Arg494Gln) in *SERPING1*. When the mutant protein was expressed in a murine hepatoma cell line, analysis of the supernatant failed to detect the protein, which was instead abundant in the insoluble fraction of cell lysate. We hypothesize that intracellular accumulation is due to polymer formation.

Our findings indicate that p.Arg494Gln-C1-INH allows protein synthesis, but impaired cytoplasmic secretion. The same condition seems to apply to the other *SERPING1* structural variants that cause recessive forms of HAE. No homozygous p.Arg494Gln has ever been described and we cannot conclude that also this mutant leads to recessive HAE. However, variability of C1-INH plasma levels in this as in other variants that are clinically silent in heterozygous presentation, suggests a lower degree of impairment in the synthesis of functional C1-INH. Whether this is due to the mutant contributing to C1-INH function in plasma or to the mutant that has reduced trans-inhibition effect on the wild type allele remains to be elucidated.

The study was approved by Milano Area 1 Ethics Board, approval number 11846/2017. Informed consent to publish has been obtained from the patients.

### O16 Activation of complement MASP-3 in healthy donors and in patients with C1-inhibitor deficiency

#### Gábor Oroszlán^1^, Gábor Pál^2^, Péter Závodszky^1^, Henriette Farkas^3^, Péter Gál^1^, József Dobó^1,*^

##### ^1^Institute of Enzymology, Research Centre for Natural Sciences, Hungarian Academy of Sciences, Budapest, Hungary; ^2^Department of Biochemistry, Eötvös Loránd University, Budapest, Hungary; ^3^Hungarian Angioedema Reference Center, 3^rd^ Department of Internal Medicine, Semmelweis University, Budapest, Hungary

###### **Correspondence:** József Dobó (dobo.jozsef@ttk.mta.hu)

*Allergy, Asthma & Clinical Immunology* 2019, **15(Suppl 4):**O16

Activation of the lectin pathway of complement is initiated by mannose-binding lectin (MBL)-associated serine proteases 1 and 2 (MASP-1 and MASP-2). MASP-1 and MASP-2 circulate in the blood as zymogens in complex with pattern recognition molecules (PRMs), such as MBL, other collectins, and ficolins. The third serine protease of the lectin pathway (MASP-3), which is also complexed with PRMs, was shown to be the major physiological activator of pro-factor D (pro-FD) in the blood, linking the alternative and the lectin complement pathways. We have demonstrated earlier that only activated MASP-3 is capable of converting pro-FD to factor D (FD), and indeed the major form of MASP-3 in the blood is the active form. The activation mechanism of MASP-3, however, remains unclear. *In vitro* MASP-1 can activate MASP-3, and C1-inhibitor is the major physiological regulator of MASP-1. We hypothesized that if MASP-1 is the physiological activator of MASP-3 then individuals with low C1-inhibitor levels would exhibit altered MASP-3 activation.

The activation state of endogenous MASP-3 was detected by Western blot, whereas in other experiments fluorescently labeled recombinant MASP-3 variants were used. We found that a significant portion of full-length, labeled MASP-3 became “activated” (cleaved) in hirudin-plasma in the matter of hours even when the inactive S664A variant was used. The activation was less efficient, but still occurred, when the N-terminally truncated catalytic fragment was used. On the other hand, we found that the ratio of active MASP-3 in type I HAE patients was virtually identical with that in healthy individuals, namely 82 ± 3%, versus 81 ± 4%. This indicates that a protease other than MASP-1 is responsible for the activation of MASP-3. To confirm this assumption we monitored the cleavage of labeled MASP-3 in the presence or absence of a MASP-1-specific inhibitor. Again, no difference was observed.

In conclusion, our results imply that a protease is present in the blood that converts MASP-3 to the active form. The activation is not autoactivation because the inactive variant got cleaved as well. Activation is more pronounced with the full length protein implying that binding of MASP-3 to PRMs might be necessary for efficient activation. Activation of MASP-3 is probably carried out by a protease not inhibited by C1-inhibitor.

### O17 Simultaneous determination of human plasma serine proteases complexed with C1-inhibitor in vivo

#### Zsófia Jandrasics^1,*^, Erika Kajdácsi^1,*#^, Nóra Veszeli^2,4^, Vera Makó^4^, Anna Koncz^1^, Kinga Viktória Kőhalmi^1^, László Cervenak^1^, Péter Gál^3^, József Dobó^3^, Steven de Maat^5^, Coen Maas^5^, Henriette Farkas^1,2^, Lilian Varga^1,2^

##### ^1^Research Laboratory, 3^rd^ Department of Internal Medicine, Semmelweis University, Budapest, Hungary; ^2^Hungarian Angioedema Reference Center, 3^rd^ Department of Internal Medicine, Semmelweis University, Budapest, Hungary; ^3^Institute of Enzymology, Research Centre for Natural Sciences, Hungarian Academy of Sciences, Budapest, Hungary; ^4^MTA-SE Research Group of Immunology and Hematology, Hungarian Academy of Sciences and Semmelweis University, Budapest, Hungary; ^5^Department of Clinical Chemistry and Haematology, University Medical Center, Utrecht, The Netherlands

###### **Correspondence:** Erika Kajdácsi (erika.kajdacsi@gmail.com)

*Allergy, Asthma & Clinical Immunology* 2019, **15(Suppl 4):**O17

^#^The first authorship is equally distributed

C1-inhibitor (C1-INH) is an important regulator of complement, coagulation, fibrinolytic and contact systems. The quantity of enzyme/C1-INH complexes in the blood may well be proportional to the level of the in vivo activation of these four cascade-like plasma enzyme systems. Parallel determination of C1-INH-containing activation complexes would be important to understand the role of C1-INH in the regulation of plasma enzyme cascades in diseases like hereditary angioedema due to C1-INH deficiency (C1-INH-HAE).

We developed 10 in-house ELISAs for measuring the complexes of C1-INH and the following proteases: Factor XIa (FXIa), Factor XIIa (FXIIa), C1r, C1s, thrombin (TR), MASP1 (M1), MASP2 (M2), kallikrein (KK), and for measuring C1-INH concentration and activity. We measured the levels of the complexes in EDTA plasma from 6 healthy controls, from 5 and 5 C1-INH-HAE patients type I and type II in remission, and from 5 C1-INH-HAE patients during attack. We also measured the levels of these complexes in the blood samples taken from one C1-INH-HAE patient, during a subcutaneous attack from the start of the prodromal symptoms during the edematous attack till the spontaneous termination (attack follow up).

There was no significant difference in total level of the measured enzyme/C1-INH complexes with the exception of KK- and M1/C1-INH complexes between the controls and the patients, and of FXIa- and M2/C1-INH complexes between C1-INH-HAE type I and II patienst. When generated a ratio from the amount of the enzyme/C1-INH complex divided with C1-INH activity we found elevated FXIa-, C1r-, KK-, M1-, C1s- and TR/C1-INH complex amounts in patients compared to controls. We found no significant differences among the amount of the complexes compared in attack and remission. During the attack follow up the kinetics of the change of the complexes’ amount is very fast

From this results we can conclude that the change of the C1-INH concentration, activity or the amount of the measured complex levels can not totally explain when and why a patient will have an attack. The pathomechanism of the attack formation may has other important factors which are till unknown. Maybe the local C1-INH production (for example generated by endothelial cells) also take part in the attack formation. The fast changes in the amount of enzyme/C1-INH complexes during the follow up study may reveal that we need a very strict timing if we want to make a good comparison between the amounts of the complexes during attacks.

Supported by OTKA 112110

### O18 Bradykinin, LPS and MASP-1 synergistically regulate endothelial permeability

#### Zsuzsanna Németh^1,*^, Márta L. Debreczeni^1^, József Dobó^2^, Péter Gál^2^, László Cervenak^1^

##### ^1^3^rd^ Department of Internal Medicine, Semmelweis University, Budapest, Hungary; ^2^nstitute of Enzymology, Research Centre for Natural Sciences, Hungarian Academy of Sciences, Budapest, Hungary

###### **Correspondence:** Zsuzsanna Németh (nemethzsanna@gmail.com)

*Allergy, Asthma & Clinical Immunology* 2019, **15(Suppl 4):**O18

**Background:** Bradykinin is considered as the major mediator of edema in HAE, but we have only limited knowledge about the triggering factors and the initiation of the attacks. Bacterial infections are considered as one of the risk factors for the onset of attacks and during these the complement system becomes activated. We have previously demonstrated that the most abundant enzyme of the complement lectin pathway, mannan-binding lectin-associated serine protease 1 (MASP-1), which is naturally inhibited by C1-inhibitor, directly increases endothelial permeability and activates the expression of permeability related genes. Therefore, we wanted to investigate how bacterial LPS, bradykinin and MASP-1 can interact with one another to influence endothelial permeability.

**Results and methods:** We measured the mRNA level of BDKRB1 and 2 with qPCR and found that MASP-1 upregulated the level of BDKRB2, while LPS increased the expression of both bradykinin receptors. In concert with this, the MASP-1 or LPS pretreated cells showed significantly greater Ca^2+^-mobilization to bradykinin than those that were not pretreated (measured with fluorescence microscopy).

LPS also induced the mRNA level of PAR2, which is a receptor of MASP-1 on endothelial cells, and we demonstrated that MASP-1 elicited grater Ca^+^-mobilization after LPS pretreatment.

To measure the endothelial permeability, we used the modified X-per-T method. The LPS pretreatment could significantly increase endothelial permeability in response to MASP-1.

**Conclusion:** Our findings highlight that significant interaction can occur amongst endothelial cell activators (between MASP-1 and LPS, LPS and bradykinin and MASP-1 and bradykinin) in the regulation of endothelial cell permeability. These synergistic interactions may give us a more detailed picture on the pathogenesis of HAE and highlight the importance of MASP-1 as a potential additional factor triggering edematous attacks.

**Supported by:** Hungarian Scientific Research Fund (OTKA K115623)

### O19 Pharmacological profile of PHA-022121, a non-peptide bradykinin B2 receptor antagonist, established using the isolated human umbilical vein

#### François Marceau^1,*^, Anne S.J. Lesage^2^, Jochen Knolle^2^, Christoph Gibson^3^

##### ^1^Axe Microbiologie-Infectiologie et Immunologie, Research Center, CHU de Québec-Université Laval, Québec, QC, Canada; ^2^Pharvaris, Leiden, The Netherlands; ^3^AnalytiCon Discovery, Potsdam, Germany

###### **Correspondence:** François Marceau (francois.marceau@crchudequebec.ulaval.ca)

*Allergy, Asthma & Clinical Immunology* 2019, **15(Suppl 4):**O19

**Background:** Only one ligand of the bradykinin (BK) B2 receptor is in clinical use, the cationic peptide icatibant, subcutaneously injected to abort attacks of hereditary angioedema. While non-peptide B2 antagonists have been previously reported, none has been clinically developed. We report the in vitro pharmacological characterization of the novel orally bioavailable non-peptide, small molecule B2 receptor antagonist, PHA-022121 (molecular weight ≈ 500, uncharged at pH 7.4), based on the contractility of the isolated human umbilical vein. This bioassay is suitable to assess potency, surmountability, partial agonist/antagonist activity, specificity and reversibility over a time scale of hours.

**Materials and methods:** Rings of human umbilical cord, obtained with informed consent after elective caesarean sections, were mounted in organ baths containing Krebs buffer maintained at 37 °C. Contractility studies consisted of constructing cumulative concentration-effect curves for BK; antagonists were introduced in the bathing fluid 30 min before BK. The effect of each antagonist drug at each tested concentration was calculated as the rightward shift (dose ratio [DR]) of the averaged concentration-effect curve relative to the EC50 established in matched control tissues exposed only to the vehicle. When applicable, Schild plot parameters, including the pA2 value, were estimated using the Schild plot (abscissa: -log[antagonist], ordinate: log(DR-1), linear regression) using the computer program Pharm/PCS.

**Results:** The antagonism exerted by both PHA-022121 and icatibant is surmountable (competitive). PHA-022121 is a very potent antagonist of bradykinin-elicited contractions mediated by the endogenous B2 receptor in human umbilical vein tissue. The pA2 value of 9.46 translates to a concentration of 350 pM, i.e. the non-peptide drug is 25-fold more potent than icatibant (pA2 8.06, translating to 8.71 nM) in the same system. PHA-022121 did not exert partial agonist activity up to the highest concentration tested (10 μM). Both antagonists are reversible upon washout, but less completely for the non-peptide drug, consistent with a higher affinity. Icatibant at micromolar levels is a significant antagonist of the B1 receptor expressed in the vein, whereas PHA-022121 is not.

**Conclusions:** The human umbilical vein bioassay confirms and extends the characterization of PHA-022121 in a tissue system: the drug is a competitive, reversible B2 receptor antagonist that is considerably more potent and selective than icatibant.

**Acknowledgements:** Supported by Pharvaris B.V. We thank Ms. J. Bouthillier for technical help.

### O20 Changes of coagulation parameters during erythema marginatum in patients with hereditary angioedema

#### Kinga Viktória Kőhalmi^1,*^, Blanka Mező^2^, Nóra Veszeli^2^, Szabolcs Benedek^3^, Adrienne Fehér^4^, Ágnes Holdonner^1^, Milos Jesenak^5^, Lilian Varga^1^, Henriette Farkas^1^

##### Hungarian Angioedema Reference Center, 3^rd^ Department of Internal Medicine, Semmelweis University, Budapest, Hungary; ^2^MTA-SE Research Group of Immunology and Hematology, Hungarian Academy of Sciences and Semmelweis University, Budapest, Hungary; ^3^ 3^rd^ Department of Internal Medicine, Semmelweis University, Budapest, Hungary; ^4^ Department of Laboratory Medicine, Semmelweis University, Budapest, Hungary; ^5^Department of Pediatrics, Martin University Hospital, Martin, Slovakia

###### **Correspondence:** Kinga Viktória Kőhalmi (kinga.viktoria.kohalmi@gmail.com)

*Allergy, Asthma & Clinical Immunology* 2019, **15(Suppl 4):**O20

**Background:** Hereditary angioedema caused by deficiency of the C1-inhibitor protein of the complement system (C1-INH-HAE) is characterized by recurrent episodes of subcutaneous/submucosal edema which may be preceded by erythema marginatum (EM) as a prodromal symptom. Our aim was to analyze the changes in parameters of the coagulation and fibrinolytic systems during the development of EM and HAE attack.

**Materials and methods:** Eight C1-INH-HAE patients (1 man, 7 women, median age: 45.3 years), followed-up in Angioedema Reference Centers were investigated. These patients experienced EM on several occasions during their lifetime and blood samples were obtained during EM in all cases. Four (Patient #1 to Patient #4) of eight patients had ≥ 1 blood samples taken during EM (min. 2 samples, max. 4 samples). Sodium citrate anticoagulated blood samples were obtained from all patients during symptom-free period, during EM and during HAE attack. The following coagulation and fibrinolytic parameters were measured in each sample: prothrombin time (PT), activated partial thromboplastin time (aPTT), fibrinogen, D-dimer, Factor V, Factor VII, Factor X, Factor XI and Factor XII. The clinical characteristics of EM’s were recorded by the *Erythema Marginatum Detailed Questionnaire*. All subjects consented to the study.

**Results:** We observed the following differences between samples taken during HAE attack vs. symptom-free period: D-dimer levels were significantly elevated [1.9 (0.80–4.80) mg/L *vs.* 0.59 (0.41–1.50) mg/L; p = 0.0391; median (25–75th percentile], while aPTT was significantly shorter during HAE attack [23.55 (22.10–26.35) sec vs. 24.85 (23.08–27.00) sec; p = 0.0159]. The levels of D-dimer were significantly higher during EM vs. symptom-free period [3.50 (0.62–12.02) mg/L vs. 0.59 (0.41–1.50) mg/L; p = 0.0078]. Between during EM and HAE attack samples, there were no significant differences regarding the investigated parameters. Analyzing the patients personally, individual changes were shown in the levels of D-dimer: in Patient #1 0/4, in Patient #2 1/2, in Patient #3 2/2, in Patient #4 0/2 D-dimer levels were higher in during EM blood samples compared to during HAE attack samples.

**Conclusions:** According to our investigations, pathophysiological changes starts during EM as D-dimer levels were elevated during the prodromal symptom. In the background of the personal variations, genetic background is likely. Nevertheless, more patients and further investigations of the complement and kinin-kallikrein systems are needed for the better understanding of the pathomechanism of EM. A new, individualized therapy, administered during EM to prevent the development of HAE attacks seems to be thoroughly grounded.

This study was supported by OTKA K124557, EFOP-3.6.3-VEKOP-16-2017-00009 and the Pharming Group NV.

### O21 Plasminogen missense mutation p.Lys330Glu: altered plasminogen glycoforms type I & II and activation susceptibility

#### Faidra Parsopoulou^1^, Delphine Charignon^2^, Fotis Psarros^3^, Maud Tengo^2^, Coen Maas^4^, Christian Drouet^5^, Anastasios E. Germenis^1^, Arije Ghannam^2,*^

##### ^1^Department of Immunology & Histocompatibility, School of Health Sciences, Faculty of Medicine, University of Thessaly, Larissa, Greece; ^2^KininX SAS, Grenoble, France; ^3^Department of Allergology, Navy Hospital, Athens, Greece; ^4^Utrecht University Medical Center, Utrecht University, Utrecht, The Netherlands; ^5^University Paris-Descartes, Cochin Institute, INSERM, Paris, France

###### **Correspondence:** Arije Ghannam (arije.ghannam@kininx.com)

*Allergy, Asthma & Clinical Immunology* 2019, **15(Suppl 4):**O21

**Background:** Hereditary angioedema with normal C1-inhibitor (nC1Inh-HAE) may be associated to specific mutations, *e.g. F12* and *ANGPT1* gene variants. Recently the new variant c.988A > G altering the plasminogen gene (*PLG*, NM_000301.3) in exon 9 was associated to nC1Inh-HAE. This variant led to the missense mutation p.Lys330Glu (K330E) in the plasminogen (PLG) kringle 3 domain.

PLG has two glycoforms, type I PLG (~ 33% of circulating PLG), which is *O*-glycosylated at Thr346 and *N*-glycosylated at Asn289 and type II PLG, with an *O*-glycosylation on Thr346 and that comprises ~ 67% of circulating forms. Type I PLG has 10-fold less affinity for cells compared to the type II (Gonzalez-Gronow et al., 2002). Conversely, type I PLG appears to function more efficiently than type II in the degradation of fibrin clots. We aimed to investigate biological phenotype of the variant pertaining to angioedema.

**Materials and methods:** Citrate plasma samples from 4 individuals from a Greek family were harvested. We investigated the index case, a 75-year old woman, homozygous carrier and presenting with severe angioedema, her two healthy daughters and her healthy granddaughter, carriers of the heterozygous mutation.

C1-inhibitor (C1Inh) function, spontaneous kallikrein activity, kinin catabolism and kininogen cleavage were investigated. PLG activation and its circulating species, with clot lysis time and basic coagulation tests were carried out.

**Results:** All individuals displayed normal C1Inh function and kallikrein activity. High molecular kininogen (HK) was found cleaved (cHK), at nearly 17% in homozygous patient in samples out of angioedema attack (reference 1–15.6%). During the active period, cHK abundance was found nearly 25% and 21%, 5 h and 24 h, after the attack, respectively.

All subjects had normal activity for carboxypeptidase N, dipeptidylpeptidase IV and angiotensin converting enzyme. But aminopeptidase P activity was low in all subjects.

The basic coagulation tests (PT, PTT, INR) were normal.

Interestingly, the anti-PLG immunoblot showed that the glycoform profiles are reversed in the homozygous patient, with ~ 60% of type I PLG. The other subjects had also altered glycoform patterns, with two bands of approximately equal intensity.

PLG activation was found enhanced by PLG activators urokinase (UK) and streptokinase (SK), more than by tissue plasminogen activator (tPA).

**Conclusion:** Different glycosylation patterns of circulating PLG have important biological impact. The enhanced PLG activation susceptibility may be related to inversed patterns, in agreement with the observation of Takada et al. (Takada and Takada, 1983) showing that type I PLG is more susceptible to activation by UK or SK than type II PLG. These observations are congruent with an increased cleaved HK, a situation likely to be associated with angioedema development.

**Consent to publish:** Written, informed consent for publication was obtained from the patient [or parent/guardian for patients under 16]

**References**

- Gonzalez-Gronow, M., Gawdi, G., and Pizzo, S.V. (2002). Tissue factor is the receptor for plasminogen type 1 on 1-LN human prostate cancer cells. Blood 99, 4562–4567.
- Takada, A., and Takada, Y. (1983). The activation of two isozymes of glu-plasminogen (I and II) by urokinase and streptokinase. Thromb. Res. 30, 633–642.

### O22 Who and when: the analysis of the molecular mechanisms of C1-inhibitor deficiency induced angioedema for the best therapeutic choice

#### Ilaria Ciani^1^, Fernand Junior Gobeil ^2^, Roberta Bulla^1^, Riccardo Addobbati^3^, Gabriella Vascotto^3^, Peter Späth^4^, Henriette Farkas^5^, Marco Cicardi^6^, Francesco Tedesco^7^, Fleur Bossi^3,*^

##### Department of Life Sciences, University of Trieste, Trieste, Italy; ^2^ Department of Pediatrics, Centre Hospitalier Universitaire (CHU) Sainte-Justine Research Center, Montréal, Québec, Canada; ^3^ Institute for Maternal and Child Health, IRCCS Burlo Garofolo, Trieste, Italy; ^4^ Institut für Pharmakologie der Universität Bern, Inselspital, Bern - Switzerland; ^5^ Hungarian Angioedema Reference Center, 3rd Department of Internal Medicine, Semmelweis University, Budapest, Hungary; ^6^ Department of Biomedical and Clinical Sciences Luigi Sacco, University of Milano, ASST Fatebenefratelli Sacco, Milano, Italy; ^7^ IRCCS, Istituto Auxologico Italiano, Milano, Italy

###### **Correspondence:** Fleur Bossi (fbossi@units.it)

*Allergy, Asthma & Clinical Immunology* 2019, **15(Suppl 4):**O22

Angioedema (AE) due to inherited or acquired deficiencies of C1-inhibitor (C1-INH) is characterized by self-limiting localized swelling of deeper layers of the skin or submucosal tissues, becoming particularly life threatening if it occurs in the upper respiratory tract. C1–INH regulates the release of bradykinin which can enhance permeability of post-capillary venules interacting with two different type of receptors, B1 and B2. Since there are different therapeutic options we investigate the molecular mechanisms that lead to the attacks, in particular the ability of C1-INH and bradykinin receptor antagonists to block the ongoing permeabilizing effect of the acute attack plasma collected from patients, in order to identify the most effective therapeutic strategy.

For this purpose, we used a transwell in vitro model with a filter covered by primary human endothelial cells (EC), in the upper chamber we add the fluorescent-BSA and the stimuli and the BSA leaked into the lower chamber was evaluated using a Fluorescence reader. We found that the presence of C1-INH (BehrinertP) was able to block the endothelial permeability induced by the plasma collected from patients during attack (APL) in the majority of the patients. To mimic the in vivo situation we stimulated the EC with the APL for 30 min and then the SN was collected and used to stimulate the ECs in the transwell model. In that case the inhibition of the leakage by C1INH was not seen in all the patients. This observation was further confirmed by using the plasma collected from patients before and 1 h after the clinical treatment with C1-INH. The addition of C1-inhibitor from 10 min before the addition of APL and till 10 min after the addition of APL resulted efficient in the inhibition of endothelial leakage, while the use of C1-inhibitor 20 min after the contact between the APL and the EC resulted completely inefficient. Then we added the antagonist of B1 BK receptor (R954) or the antagonist of B2 BK receptor (Icatibant) and they both resulted able to block the permeabilizing effect of the SN. This inhibition resulted even stronger using a combination of both antagonists.

On the basis of these results, we conclude that the inhibition of endothelial leakage induced by APL stimulation by C1-INH indicates the involvement of that molecule in controlling the onset of AE attacks, although the inability of C1-INH to completely block the permeabilizing effect of the SN indicates that after the activation of the cells there are other molecules involved. The most plausible is BK but also other related metabolites can interact with specific receptors. The effectiveness of the treatment seems to be correlated with the time of treatment, as soon is treated the patient C1-INH is perfectly working but after the activation of the kinin system antagonists of kinins receptors seem to be more efficient in reducing the vascular permeability.

### O23 PHA-022121, the first-in-class orally active bradykinin receptor B2 antagonist for on-demand and prophylactic treatment of HAE

#### Anne S.J. Lesage^1,*^, Jochen Knolle^1^, François Marceau^3^, Christoph Gibson^2^

##### ^1^Pharvaris, Leiden, The Netherlands; ^2^AnalytiCon Discovery, Potsdam, Germany; ^3^Axe Microbiologie-Infectiologie et Immunologie, Research Center, CHU de Québec-Université Laval, Québec, QC, Canada

###### **Correspondence:** Anne S.J. Lesage (anne.lesage@pharvaris.com)

*Allergy, Asthma & Clinical Immunology* 2019, **15(Suppl 4):**O23

Patients are eagerly awaiting next generation treatments for hereditary angioedema (HAE), asking for oral treatment to replace the burden of current injectables. Pharvaris is developing PHA-022121 as a first-in-class novel proprietary small-molecule antagonist of the B2 receptor, for oral on-demand treatment of acute HAE attacks and for prophylactic prevention of attacks. PHA-022121 is entering clinical phase I studies in Q2 2019, was optimized and developed by Pharvaris, a company founded by the team which successfully developed Firazyr the only approved and widely used B2 antagonist for on demand treatment. Based on preclinical studies, PHA-022121 demonstrates excellent drug-like physicochemical properties, primary activity, oral bioavailability and metabolic stability. PHA-022121 shows sub-nanomolar potency in a calcium mobilization assay using recombinant human B2 receptors expressed in a mammalian cell line (0.15 nM) and at endogenous human B2 receptors in the human umbilical vein model (pA2 value corresponding to 0.35 nM). The compound is several thousand-fold selective for the B2 receptor versus the B1 receptor as well as against 130 other molecular targets (including GPCRs, ion channels, enzymes and transporters). Oral bioavailability is high in rat and monkey. In a proof of concept study, PHA-022121 potently inhibits bradykinin-induced haemodynamic changes in freely moving monkeys. The onset of activity was 1 h or less (first time point measured), which was faster than icatibant. PHA-022121 also partially prevents carrageenan-induced paw edema in rat with a longer duration of action as compared to icatibant. Based on experimental data and modeling, Pharvaris expects that a single daily pill of less than 30 mg will provide therapeutic efficacy for at least 24 h. Pharvaris plans to develop PHA-022121 as an oral on-demand and prophylactic treatment of HAE attacks.

### O24 Clinical evaluation of pharmacokinetics, pharmacodynamics, safety, and efficacy dose–response of BCX7353 as an acute treatment for angioedema in patients with hereditary angioedema (HAE)

#### Marcin Stobiecki^1,*^, Emel Aygören-Pürsün^2^, Shimalee Andarawewa^2^, Andrea Zanichelli^3^, Marco Cicardi^3^, Aarnoud Huissoon^4^, Dumitru Moldovan^5^, Noémi Bara^5^, Enikő Mihály^5^, Markus Magerl^6^, Mauro Cancian^7^, Ricardo Senter^7^, Ania Manson^8^, Vesna Grivcheva-Panovska^9^, David Hagin^10^, Urs Steiner^11^, Sorena Kiani-Alikhan^12^, Nancy Agmon-Levin^13^, Anette Bygum^14^, W. Aberer^15^, Saul N. Faust^16^, David Launay^17^, Mark Gompels^18^, Massimo Triggiani^19^, Claire Bethune^20^, Avner Reshef^21^, Kinga Viktoria Kohalmi^22^, Henriette Farkas^22^, Amanda Mathis^23^, Melanie Cornpropst^23^, Sylvia Dobo^23^, Sharon Van Dyke^23^, Sharon Murray^23^, Phillip Collis^23^, William P. Sheridan^23^, Marcus Maurer^6^, Hilary Longhurst^8^

##### ^1^Jagiellonian University Medical College, Krakow, Poland; ^2^Department for Children and Adolescents, Angioedema Centre, University Hospital Frankfurt, Goethe University, Frankfurt, Germany; ^3^Department of Biomedical and Clinical Sciences, Luigi Sacco Hospital, University of Milan, ASST Fatebenefratelli Sacco, Milan, Italy; ^4^Birmingham Heartlands Hospital, Birmingham, United Kingdom; ^5^University of Medicine and Pharmacy of Tîrgu Mures, Mures County Hospital, Tîrgu Mureş, Romania; ^6^Department of Dermatology and Allergy, Charité - Universitätsmedizin Berlin, Berlin, Germany; ^7^Department of Medicine, University of Padova, Padova, Italy; ^8^Department of Immunology, Addenbrooke’s Hospital, Cambridge University Hospitals NHS Foundation Trust, Cambridge, United Kingdom; ^9^Public Health Institution University Clinic of Dermatology, School of Medicine, University Sts. Cyril and Methodius, Skopje, The former Yugoslav Republic of Macedonia; ^10^Allergy and Clinical Immunology Unit, Tel-Aviv Sourasky Medical Center, Tel-Aviv University, Israel, Tel–Aviv, Israel; ^11^Department of Clinical Immunology, University Hospital Zurich, Zurich, Switzerland; ^12^Department of Immunology, Barts Health NHS Trust, Royal London Hospital, London, United Kingdom; ^13^Zabludowicz Center for Autoimmune Diseases, Chaim Sheba Medical Center, Tel Hashomer, Ramat-Gan, Israel; ^14^Department of Dermatology and Allergy Centre, Odense University Hospital, Odense, Denmark; ^15^Department of Dermatology and Venereology, Medical University of Graz, Graz, Austria; ^16^NIHR Clinical Research Facility, Southampton General Hospital, Southampton, United Kingdom; ^17^Department of Internal Medicine, Claude Huriez Hospital, Lille, France; ^18^North Bristol NHS Trust, Southmead Hospital, Bristol, United Kingdom; ^19^Division of Allergy and Clinical Immunology, University of Salerno, Salerno, Italy; ^20^Department of Immunology and Allergy, University Hospital Plymouth NHS Trust, Plymouth, United Kingdom; ^21^Allergy, Immunology and Angioedema Center, Barzilai Medical Center, Ashkelon, Israel; ^22^Hungarian Angioedema Reference Center, Third Department of Internal Medicine, Semmelweis University, Budapest, Hungary; ^23^BioCryst Pharmaceuticals, Inc., Durham, NC, USA

###### **Correspondence:** Marcin Stobiecki (marcin.stobiecki@uj.edu.pl)

*Allergy, Asthma & Clinical Immunology* 2019, **15(Suppl 4):**O24

**Background:** Approved acute treatments for angioedema attacks in hereditary angioedema (HAE) are administered parenterally, including plasma kallikrein inhibitors. Guidelines recommend at–home on–demand treatment. BCX7353, an orally administered kallikrein inhibitor with fast onset and sustained duration of action, may improve access to self-administered treatment and allow earlier dosing after symptom onset.

**Methods:** BCX7353 doses for evaluation of efficacy in HAE subjects were selected using pharmacokinetic (PK) and pharmacodynamic (PD) evidence from the first-in-human trial in healthy subjects (dose range: 30 to 1000 mg). Separately, plasma concentration–time profiles, plasma kallikrein inhibition (KKI)-time profiles, and PK-PD relationships of orally administered BCX7353 (750 mg) were evaluated in 6 HAE subjects. We separately conducted a proof-of-concept clinical trial (ZENITH-1) using a 3-part, dose-de-escalation, randomised-sequence, double-blind, placebo-controlled, 3-period crossover design. Subjects were randomised in each part to 2 treatments with BCX7353 (at the same dose level) and 1 with placebo; for each treatment, subjects confirmed the attack with the investigator before dosing. This trial tested on–demand at-home treatment of angioedema attacks with 750, 500, and 250 mg doses of BCX7353 in 58 subjects with HAE. Subjects recorded symptoms and interventions using standardised questions and visual analogue scales (VAS) for skin swelling, skin pain, and abdominal pain in a diary.

**Results:** Drug levels (Table 1) exceeded 8 × half-maximal effective concentration (EC_50_) for KKI (approximately lower limit of normal for C1INH effect on KKI) within 30 min of dosing and were sustained for > 24 h in all 6 HAE subjects administered BCX7353 750 mg [1]. Single doses of ≤ 500 mg in healthy subjects did not provide uniformly rapid or sustained PK or PD effects. KKI was strongly correlated with plasma drug levels in HAE subjects [1] and healthy subjects [2]. In ZENITH-1, at–home oral BCX7353 was taken 32–35 min (median across dose levels) after onset of symptoms. Predose mean composite VAS scores (active/placebo) were 15/11, 18/13, and 14/15 mm for 250, 500, and 750 mg; least squares mean differences from placebo in change from baseline composite VAS at 4 h postdose were + 0.57, – 2.10, and – 6.98 mm for the 250, 500, and 750 mg doses. Differences from placebo in additional endpoints are detailed in Table 2. The 750 mg dose showed the best results. BCX7353 was generally safe and well–tolerated at all doses.

**Table 1:** PK and PD parameters in healthy subjects and HAE subjects after single oral single doses of BCX7353

Population	N	Dose of BCX7353 mg	C_max_^a^ ng/mL	AUC_0-24_^a^ ng h/mL	[7353] > 8×EC_50_ at 0.5,1,8,24 h n	KKI at 30 min^b^ %	KKI at 24 h^b^ %
Healthy subjects	6	250	104 (24)	995 (25)	0,3,1,0	12 (44)	24 (18)
Healthy subjects	6	500	245 (59)	2700 (43)	0,4,6,0	50 (26)	72 (10)
HAE subjects^c^	6	750	584 (26)	5670 (17)	6,6,6,6	80 (7)	81 (11)

^a^PK parameters are geometric mean (% coefficient of variation)

^b^PD parameters are mean (standard deviation) in an in ex vivo assay (note that n = 5 in HAE subjects), and results reported are percent reduction in specific amidolytic activity from pre-dose sample. As the reagent volume dilutes the plasma sample 4-fold, BCX7353 concentration *ex* *vivo* is 25% of that present *in vivo* postdosing. The reported ex vivo % inhibition therefore underestimates the in vivo inhibitory activity achieved. For example, a value of 80% inhibition ex vivo corresponds to approximately > 94% inhibition in vivo

^c^PK study in subjects with HAE was conducted at an HAE reference center at University Hospital, Frankfurt, Germany

**Table 2:** Efficacy endpoints (difference, active vs placebo) for each dose level in ZENITH-1. Phase 2 trial of oral BCX7353

Endpoint^a^	250 mg	500 mg	750 mg
*Number of attacks, active/placebo*	*21/11*	*25/11*	*64/31*
Change from baseline in VAS score at 4 h, mm^a^	0.57	− 2.1	− 6.98***
Improved or stable VAS at 4 h, %^b^	6.4	18.5	21.0*
Improved or stable VAS at 24 h, %^b^	14.5	23.6	27.0*
Improved or stable symptoms at 4 h, %^b^	12.6	12.4	22.3*
Improved or stable symptoms at 24 h, %^b^	11.6	27.6	28.6**
Standard of care rescue treatment at 24 h, %^b^	− 7.4	− 13.5	− 31.6***
No or mild symptoms at 24 hr^b^	16.4	23.6	31.8***

^a^LS mean difference to placebo reported. P-value generated from a mixed effect linear model including treatment, period, and sequence as fixed effects, subject within sequence as a random effect, and predose 3-symptom composite VAS score as a covariate

^b^Difference to placebo in proportion of attacks achieving the endpoint reported. P-value generated from a generalised logistic model including treatment, period, and sequence as fixed effects, and subject within sequence as a random effect. Use of standard of care HAE medication = failure for endpoint

*** p < 0.005; ** p < 0.01; * p < 0.05 for active vs placebo

**Conclusions:** The observed PK, PD, treatment effects, dose–response, and safety and tolerability profile of oral BCX7353 warrant confirmatory Phase 3 evaluation of the 750 mg dose as an acute treatment for angioedema attacks in HAE.

**Trial Registration:** NCT03240133

**References**

1. Mathis A, DeSpirito M, Cornpropst M, Sheridan WP, Aygören–Pürsün E: Oral Administration of a Liquid Formulation of BCX7353 Rapidly Provides Sustained Concentrations and Kallikrein Inhibition. Annals Allergy Asthma Immunol. 2018; 121(5): S32.
2. Cornpropst M, Dobo S, Collier J, Rose A, Wilson R, Babu YS, Collis P, Sheridan W: BCX7353, a Potent Inhibitor of Plasma Kallikrein, Shows Sustained Maximal Enzyme Inhibition When Dosed Orally Once Daily: Results from a Phase I Trial in Healthy Subjects. J Allergy Clin Immunol. 2016; 137(2): AB401.

### O25 KVD900, a new oral on‐demand treatment of hereditary angioedema attacks achieves complete plasma kallikrein suppression: safety, tolerability, pharmacokinetic and pharmacodynamic results from a phase 1 first-in‐human study

#### Andreas Maetzel^1,*^, Michael S. Smith^1^, Edward J. Duckworth^2^, Sally L. Hampton^2^, Gian Marco De Donatis^2^, Nivetha I. Murugesan^1^, Louise J. Rushbrooke^2^, Lily Li^1^, Danielle Francombe^3^, Edward P. Feener^1^, Christopher M. Yea^2^

##### ^1^KalVista Pharmaceuticals, Cambridge, MA, United States; ^2^KalVista Pharmaceuticals, Tetricus Science Park, Porton Down, Salisbury, United Kingdom; ^3^Simbec Research Ltd, Merthyr Tydfil, Mid Glamorgan, Wales, United Kingdom

###### **Correspondence:** Andreas Maetzel (andreas.maetzel@kalvista.com)

*Allergy, Asthma & Clinical Immunology* 2019, **15(Suppl 4):**O25

**Background:** Attacks of swelling and pain in hereditary angioedema are attributed to increased vascular permeability due to excessive and uncontrolled formation of the proinflammatory peptide hormone bradykinin (BK). BK is generated through cleavage of high molecular weight kininogen (HK) by the serine protease plasma kallikrein (PKa). KVD900 is a novel, potent (Ki 3 nM), selective inhibitor of PKa. An orally available and rapidly absorbed PKa inhibitor could provide a new therapeutic opportunity to halt and resolve HAE attacks early.

**Methods:** We conducted a first in human study to evaluate the safety, tolerability, pharmacokinetics and pharmacodynamics of KVD900 in healthy adult males aged 18 to 55 years. In Part A we investigated single ascending doses (5 to 600 mg) of KVD900 capsule (a powder in capsule formulation); 8 cohorts of 8 subjects with 6 active treatment and 2 placebo subjects per cohort. In Part B we compared single 100 mg doses of KVD900 tablet and KVD900 capsule in a crossover design involving 8 subjects. In Part C we compared single 600 mg doses of KVD900 100 mg tablets under fed and fasting conditions in a crossover design involving 12 subjects. Plasma kallikrein enzyme activity was measured in dextran sulfate stimulated whole plasma samples using a fluorogenic enzyme assay and a capillary based high molecular weight kininogen (HK) cleavage immunoassay.

**Results:** Overall, 68 healthy males received KVD900. KVD900 was generally safe and well tolerated without any severe or serious adverse events and without related gastrointestinal adverse events. All adverse events were mild, except for one AE (headache) in the 10 mg cohort of Part A, which was considered moderate. Mean maximum plasma concentration with the 600 mg capsule reached 4.830 ng/mL (± 1.080). Similar exposures were reached with 600 mg tablets under fed and fasted conditions (area under the curve – 0 to infinity [AUC _0‐inf_] 21,200 h*ng/mL vs. 19,800 h*ng/mL. Complete PKa inhibition (99.3%) was achieved on 600 mg, fasted and fed (CI: 99.0% ‐ 99.5%); > 85% PKa inhibition was maintained for 8 to 10 h. KVD900 rapidly provided HK cleavage protection for at least 10 h consistent with the PKa enzyme inhibition data.

**Conclusion:** This first‐in‐human study of KVD900 showed that a single oral administration of up to 600 mg KVD900 is generally safe and well tolerated without any severe adverse events. KVD900 achieves rapid suppression of PKa activity.

### O26 Pharmacokinetics, safety, and potency of ATN-249, a novel oral plasma kallikrein inhibitor for hereditary angioedema

#### Ira Kalfus^1,*^, Elliot Offman^2^, Andrew McDonald^1^

##### ^1^Attune Pharmaceuticals, Inc., New York, NY, United States^; 2^Certara Strategic Consulting, Toronto, ON, Canada

###### **Correspondence:** Ira Kalfus (ikalfus@aol.com)

*Allergy, Asthma & Clinical Immunology* 2019, **15(Suppl 4):**O26

**Background:** Hereditary angioedema (HAE) is a rare, potentially life-threatening disease characterized by acute skin and mucosal oedema. Currently available treatments are administered s.c. or i.v., thus there is a need for well tolerated, orally-administered therapies. ATN-249 is a novel oral plasma kallikrein inhibitor designed to treat HAE by blocking kallikrein-mediated production of bradykinin. ATN-249 showed dose linear pharmacokinetics and minimal food effects in a phase 1 single ascending dose study in healthy volunteers. Here we report ATN-249’s pharmacokinetics (PK) and safety from a 14-day multiple ascending dose study and its in vitro potency relative to an approved plasma kallikrein inhibitor.

**Materials and methods:** Healthy participants received multiple doses of ATN-249 100 mg QD, 200 mg QD, 400 mg QD, 300 mg BID, or placebo for 14 days (6 ATN-249:2 placebo in each dose cohort). Serial blood draws and urinalysis were conducted to calculate PK parameters. Adverse events (AEs) were assessed. Separately, the potency of ATN-249 and lanadelumab, a subcutaneously administered plasma kallikrein inhibitor, were tested in biochemical inhibition and contact activation assays in human plasma as well as in a semi-quantitative Western blot assay evaluating attenuation of cleaved kininogen.

**Results:** PK parameters following 14-day QD regimens were dose proportional (Table 1). With repeated dosing over 14-days, trough concentrations were on average approximately 200 ng/mL (460 nM) and generally exceeded 527 ng/mL (1.2 µM) for 400 mg QD and 300 mg BID regimens, respectively. Average concentrations at steady-state (C_avg_) were approximately 683 ng/mL (1.57 µM) and 1198 ng/mL (2.7 µM) for the 400 mg QD and 300 mg BID regimens, respectively. Across treatment and placebo cohorts, the most common AEs were headache, contact dermatitis secondary to ECG lead placement, and nausea. All AEs were self-limited and not related to study drug. Mean ATN-249 trough concentrations for the 400 mg QD and 300 mg BID dosages exceeded expected therapeutically relevant concentrations and were greater than those demonstrating attenuation of cHMWK (~ 250–500 nM) as assessed by Western blot analysis of activated whole plasma. In the biochemical assay, the potency of lanadelumab and ATN-249 converged (Fig. 1) and in the contact activation assay, the potency of ATN-249 exceeded lanadelumab above the EC_90_ (Fig. 2).

**Table 1:** Mean (% CV) PK parameters by dose on day 14 in MAD study (n = 6 per cohort)

Parameter	Dose (mg)			
	100 QD	200 QD	400 QD	300 BID
AUC_tau_ (ng*hr/mL)	4592 (26.9)	9749 (27.2)	16,390 (47.8)	28,740 (26.4)
C_avg_ (ng/mL)	191 (26.9)	406 (27.2)	683 (47.8)	1198 (26.4)
C_max_ (ng/mL)	496 (22.7)	1202 (26.1)	2010 (60.5)	2040 (28.6)
C_min_ (ng/mL)	47 (36.0)	92 (44.5)	153 (45.1)	527* (28.2)
T_max_ (Hours)	2.4 (35.6)	2.2 (49.9)	1.9 (43.2)	11.1 (58.8)
Half-Life (Hours)	10.9 (8.1)	10.5 (30.3)	11.7 (20.4)	7.7 (7.6)

*n = *5*

**Fig. 1:**
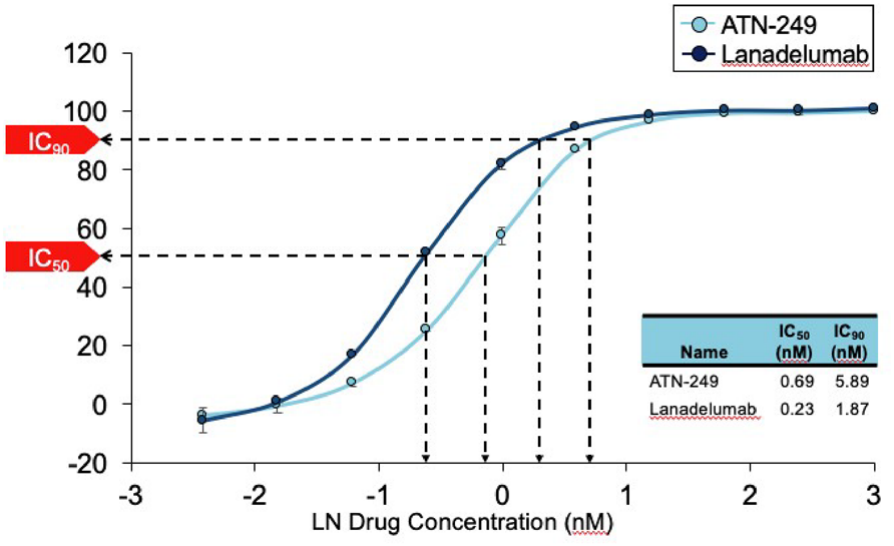
Inhibition of plasma kallikrein via biochemical inhibition (percent inhibition)

**Fig. 2:**
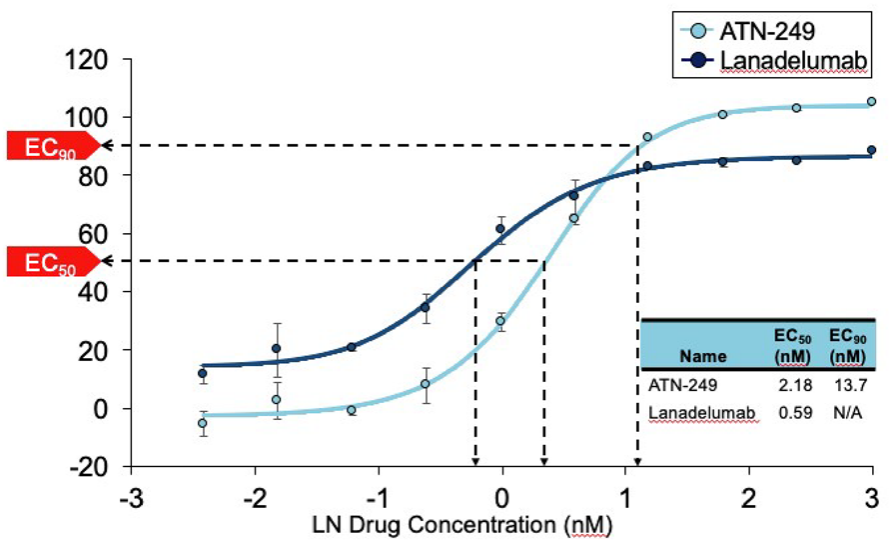
Inhibition of plasma kallikrein via contact activation assay in plasma (percent inhibition)

**Conclusions:** ATN-249’s PK were dose-linear with low to moderate between-subject variability. Repeat dose trough ATN-249 concentrations were above predicted therapeutic concentrations in ex vivo assays of contact activation. ATN-249 was well-tolerated and no AEs were drug related. ATN249 demonstrated potent kallikrein inhibition comparable to lanadelumab in biochemical and exvivo contact activation assays, including Western blot detection of cleaved kininogen. These results, along with predictable PK, suggest ATN-249 may be a potent, safe, oral plasma kallikrein inhibitor for prophylactic treatment of HAE.

### O27 Population pharmacokinetic analysis of C1-esterase inhibitor functional activity in the COMPACT open-label extension study is consistent with previous COMPACT studies

#### Bruce Zuraw^1^, Theresa Yuraszeck^2^, Ingo Pragst^3^, Dipti Pawaskar^2^, Fiona Glassman^2,*^

##### ^1^Department of Medicine, University of California San Diego and San Diego VA Healthcare, La Jolla, CA, United States; ^2^CSL Behring, King of Prussia, PA, United States; ^3^CSL Behring GmbH, Marburg, Germany

###### **Correspondence:** Fiona Glassman (Fiona.Glassman@cslbehring.com)

*Allergy, Asthma & Clinical Immunology* 2019, **15(Suppl 4):**O27

**Background:** In patients with hereditary angioedema (HAE), the relationship between increased risk of attacks and deficiency in complement component 1 (C1) esterase inhibitor (C1-INH) functional activity (C1-INH[f]) is well established. The objective of this study was to characterise the pharmacokinetics (PK) of subcutaneous (SC) C1-INH in patients with HAE enrolled in the COMPACT open-label extension (OLE) study using a population PK model previously developed using data from prior COMPACT studies. Variation in PK of C1-INH(SC) in different age groups was also evaluated.

**Materials and methods:** C1-INH(f) was measured in four trials (COMPACT Phase I, II, III and OLE study [NCT1760343, NCT01912456, NCT01576523 and NCT02316353, respectively]). Previously, a one-compartment model with first-order elimination and bodyweight effect on clearance was used to describe the PK of C1-INH(f) after C1-INH(SC) administration in patients with HAE. One thousand profiles for subjects with HAE were simulated based on the distribution of individual weights from COMPACT OLE to assess whether the previously described model could characterise the PK of C1-INH(SC) in patients who completed COMPACT OLE. Post-hoc clearance estimates were visually evaluated. Age groups evaluated for variation were < 12, 12–17, > 17– < 65 and ≥ 65 years old.

**Results:** The previously developed population PK model was able to characterise observed C1-INH(f) following C1-INH(SC) administration in patients completing COMPACT OLE. Simulations of steady-state C1-INH(f) following C1-INH(SC) administration at 40 IU/kg and 60 IU/kg doses captured most data from the COMPACT OLE study within the prediction interval, suggesting that the previously developed model was consistent with the observed C1-INH(f) in patients in COMPACT OLE (Fig. 1). Visual evaluation of individual post hoc clearance estimates revealed clearance in C1-INH(SC)-naive subjects in COMPACT OLE was similar to those in COMPACT Phase I–III studies. Individual post hoc clearance estimates were also similar across investigated age groups for paediatric, adolescent, adult and geriatric subjects. The relationship between clearance and body weight in the previously developed model was found to be applicable to the relationship observed in COMPACT OLE.

**Fig. 1:**
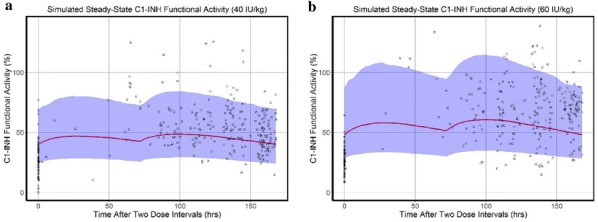
Simulations of steady-state complement component 1 (C1)-esterase inhibitor functional activity (C1-INH[f]) and observed C1-INH(f) concentrations following the administration of subcutaneous (SC) C1-INH at **a** 40 IU/kg and **b** 60 IU/kg doses in the COMPACT open-label extension study

**Conclusions:** Overall, the final analysis of COMPACT OLE was consistent with the analysis performed for prior COMPACT studies. Population PK analysis with data from the COMPACT OLE demonstrated that the previously developed population PK model could predict observed C1-INH(f) in the COMPACT OLE without bias. Population PK parameters remained unchanged and C1-INH(f) was similar across all COMPACT studies following C1-INH(SC) administration. In addition, the PK of C1-INH(SC) was similar in paediatric, adolescent, adult, and geriatric subjects.

### O28 Functional C1-Esterase inhibitor and complement protein 4 levels were not altered by lanadelumab treatment in HAE patients in the phase 3 HELP study

#### Archana Kapoor, Zhiwei Zhou, Dan Sexton, Kim Paes, Peng Lu, Priya Chockalingam^*^

##### Shire (now part of Takeda), Cambridge, MA, United States

###### **Correspondence:** Priya Chockalingam (priya.chockalingam@takeda.com)

*Allergy, Asthma & Clinical Immunology* 2019, **15(Suppl 4):**O28

**Objective:** The HELP Study^®^ is a multicenter, randomized, double-blind, placebo-controlled Phase 3 study to evaluate the efficacy and safety of lanadelumab for long-term prophylaxis against acute attacks of hereditary angioedema (HAE) in subjects with Type-I and Type-II HAE. All three lanadelumab treatment arms demonstrated statistically significant and clinically meaningful reductions in the number of HAE attacks over the 26-week treatment period compared to placebo. HAE results from variations in the *SERPING1* gene that encodes the C1-inhibitor (C1INH), a serine protease inhibitor. Reduced plasma levels of C1INH lead to enhanced activation of the contact system, triggering high levels of bradykinin and increased vascular permeability; this could lead to a feedback loop resulting in further consumption of C1INH by proteases. To investigate the hypothesis whereby plasma kallikrein inhibition minimizes C1-INH consumption and thereby affects complement protein 4 (C4) levels, we assessed the effect of lanadelumab treatment on functional C1-INH (fC1-INH) and C4 levels in HAE patients in the HELP study.

**Methods and materials:** The pharmacodynamic samples collected at Predose, Day(D)56, D98, D140 and D182 from all treatment arms: Placebo, lanadelumab 150 mg Q4 W, 300 mg Q4 W and 300 mg Q2 W were analyzed for fC1-INH and C4 levels. The chromogenic and an enzyme-linked immunosorbent assay (ELISA) methods were validated for measuring fC1-INH; an ELISA method was validated for C4. Absolute protein levels and percent normalized to baseline levels were determined for fC1-INH and C4.

**Results:** Placebo, lanadelumab 150 mg Q4 W, 300 mg Q4 W and 300 mg Q2 W arms had 41, 28, 29 and 27 subjects, respectively. The average pre-dose fC1-INH measured in HAE subjects (n = 115) was 207 mU/mL (SEM of 11 mU/mL) and 306 mU/mL (SEM of 20 mU/mL) in the ELISA and the chromogenic methods, respectively. Average C4 measured in pre-dose samples was 108 µg/mL (SEM of 11 µg/mL). No statistically significant difference was observed in fC1-INH and C4 levels in the treatment arms when multiple comparisons were made for each of the arms to placebo at all timepoints tested, and/or when pre-dose levels compared to each of the other time-points. The comparisons were also made on baseline normalized percentage changes.

**Conclusion:** Long term lanadelumab treatment does not seem to affect fC1-INH and C4 levels in HAE subjects in the HELP study suggesting that specific inhibition of plasma kallikrein may not reduce C1-INH consumption resulting in a notable increase in C4 levels. Ongoing research for other HAE pathway biomarkers in the HELP study might provide additional understanding of disease mechanisms.

### O29 Hereditary angioedema with normal C1-inhibitor: first report of an Argentinian family with factor XII mutation

#### Ricardo D. Zwiener^1,*^, Claudio Fantini^2^, Natalia Fili^3^, Mónica Maroco^4^, Paula Rozenfeld^5^

##### Department of Allergy and Immunology, Austral University Hospital, Pilar, Buenos Aires, Argentina; ^2^ Department of Allergy and Immunology, HIGA Oscar Alende, Mar del plata, Argentina; ^3^ Department of Allergy and Immunology, Hospital Materno infantil, Salta, Argentina; ^4^ Department of Allergy and Immunology, Hospital Aeronáutico, Córdoba, Argentina; ^5^ IIFP, University of La Plata-CONICET, La Plata, Buenos Aires, Argentina

###### **Correspondence:** Ricardo D. Zwiener (ricardozwiener@hotmail.com)

*Allergy, Asthma & Clinical Immunology* 2019, **15(Suppl 4):**O29

Hereditary angioedema (HAE) is a rare genetic disease associated with either a quantitative or qualitative deficiency in C1-inhibitor (C1-INH) or normal C1-INH. 1

HAE with normal C1-INH levels could be caused by mutations in different genes. Until now, there are 3 known proteins whose genes mutations lead to HAE: F12 (HAE-FXII), plasminogen (HAEPLG) and angiopoietin 1 (HAEANGPT1). 2

Approximately 30% of these cases are due to F12 gene (HAE-FXII) mutations. Point mutation (Thr328Lys or Thr328Arg), a large deletion (deletion of 72 base pairs: c.971_1018 + 24del72*) or an 18-bp duplication in the F12 (FXII) gene are detected in HAE-FXII. 3

A 42-year-old woman consulted for facial angioedema. She had 2 episodes of facial angioedema, that lasted 3 days and does not respond to treatment with corticosteroids and antihistamines, in the last 2 years without any recognized trigger,. She referred frequent abdominal pain associated with diarrhea and one of them with hypotension. A diagnosis of gastritis and irritable bowel diagnosis was provided.

She was taking levothyroxine 50mcgs/day and contraceptives (drospirenone 3 mg, ethinyl estradiol 0.03 mg).

She reported that her sister suffered from facial angioedema after a dental procedure.

After the suspicion of HAE, contraceptives were discontinued.

**Materials and methods:** Serum, citrated plasma and EDTA-blood was collected from the patients.

Antigenic values of C4 and C1-INH was assayed by turbidimetric immunoassays in serum samples from patients. Functional C1-INH activity was assayed by a chromogenic assay in plasma samples from patients.

Genetic test for F12 was assayed by sanger sequencing of exon 9 and intron–exon boundaries.

**Results:** The results of quantitative and qualitative C4 (40.15 mg/dl), C1-INH (24.75 m/dl) and functional C1-INH (154%) were normal.

Genetic test for F12 gene revealed the patient is heterozygous for the common missense mutation c.983C > A (p.Thr328Lys).

All symptoms related to angioedema disappeared after contraceptives discontinuation.

After the confirmation of diagnosis of HAE of this patient, pedigree analysis in her family led to diagnosis of 2 other patients. Time to diagnosis since first symptom was 3 years.

**Conclusions:** We described the first Argentinian family with mutation in F12 gene. Interestingly we found that the clinical episodes in the case study are mild and are related to hormonal levels. All family members are less symptomatic than other types of HAE and the delay in diagnosis was 3 years.

**Consent to publish:** Written, informed consent for publication was obtained from the patient [or parent/guardian for patients under 16]

**References**

1. Bork K. Diagnosis and treatment of hereditary angioedema with normal C1-inhibitor Allergy, Asthma & Clinical Immunology 2010, 6:15.
2. Bruce L. Zuraw. Hereditary angioedema with normal C1-inhibitor: Four types and counting. J Allergy Clin Immunol. 2018; 141 (3), 884–885.
3. Bork K, Wulff K, Hardt J, et al. Hereditary angioedema caused by missense mutations in the factor XII gene: clinical features, trigger factors, and therapy. J Allergy Clin Immunol. 2009;124(1):129‒134.

### O30 Acquired angioedema and chronic spontaneous urticaria – one disease or two separated entities? Case report

#### Milos Jesenák^1,*^, Svetlana Hadvabova^2^, Anna Bobcakova^3^, Lenka Kapustova^1^, Otilia Petrovicova^1^, Peter Banovcin^1^

##### ^1^Centre for Hereditary Angioedema, Department of Pediatrics, Jessenius Faculty of Medicine in Martin, Comenius University in Bratislava, University Teaching Hospital in Martin, Slovakia; ^2^Centre for Clinical Immunology and Allergology, Komarno, Slovakia; ^3^Centre for Hereditary Angioedema, Department of Pulmonology and Phisiology, Jessenius Faculty of Medicine in Martin, Comenius University in Bratislava, University Teaching Hospital in Martin, Slovakia

###### **Correspondence:** Milos Jesenák (jesenak@gmail.com)

*Allergy, Asthma & Clinical Immunology* 2019, **15(Suppl 4):**O30

Hereditary angioedema (HAE) represents a rare disease connected with inherited predisposition for angioedema (AE) development in various locations. More frequently, different forms of acquired angioedema can be seen in daily clinical practice. Isolated angioedema can be also a rare clinical phenotype of chronic spontaneous urticaria. Diagnostic approach and treatment strategies differ among different forms of AE.

We present a rare and unique case of 67-year-old man with negative family history for angioedema or urticaria. There were no significant diseases in the history before the onset of angioedema. He was treated for benign prostatic hyperplasia and recurrent urinal infections. At the age of 66 years, he was admitted to the hospital for severe abdominal pain, which was evaluated as acute idiopathic pancreatitis. Abdominal CT scan revealed abdominal lymphadenopathy and small lymphocytic lymphoma/chronic lymphocytic leukaemia was diagnosed. Patient was regularly followed-by haematologist without any treatment (watch&wait). 4 month after the attack of pancreatitis, recurrent facial angioedema with tongue swelling was observed and patient had at least 3 attacks. He was evaluated by clinical immunologist and laboratory profile yielded decreased concentration (0.08 g/L, N: 0.210–0.380 g/L) and function (< 40%, N: > 68%) of C1-inhibitor together with significant decline of C3 (0.307 g/L, N: 0.811–1.570) and C4 (0.01 g/L, N: 0.129–0.392) compounds of complement system. Patient was sent to the National Centre for Hereditary Angioedema in University Teaching Hospital in Martin (Slovakia). Antibodies against C1-INH were negative in all isotypes (Hungarian HAE Centre, Budapest) and significant decline of C1q was confirmed (35.0 mg/L, N: 118–238). A diagnosis of acquired angioedema I type was confirmed (with haematooncological background) and following 2 facial attacks were successfully treated with icatibant with rapid relief of symptoms within 2 h after application. Besides typical symptoms of AAE, recurrent symptoms of generalised urticaria associated with intense pruritus were observed. Treatment with desloratadine was initiated (from single dose up to 4-times increased dose), but without any clinical effect. UAS7 score was permanently above 28 points and therefore a treatment with omalizumab was initiated. After second application of omalizumab, complete remission of urticaria was succeeded.

Hereby, we present a unique case of two separated diseases – acquired angioedema of I type and chronic spontaneous urticaria. Despite their similarities, two therapeutic strategies were used for achievement of clinical control over the symptoms. We would like to point out the possible clinical co-existence of two rare diseases, what should be taken into account during the differential-diagnostic algorithm of angioedema in clinical practice.

**Consent to publish:** Written, informed consent for publication was obtained from the patient [or parent/guardian for patients under 16]

### O31 Oligoarticular juvenile idiopathic arthritis in a child with type I hereditary angioedema: a case report

#### Susan Leech^1^, Elspeth Brooker^1^, Jimmy H.C. Gooi^2,*^

##### ^1^Department of Paediatric Allergy, King’s College Hospital, London, United Kingdom; ^2^Department of Clinical Immunology, King’s College Hospital, London, United Kingdom

###### **Correspondence:** Jimmy H.C. Gooi (jimmyhc.gooi@googlemail.com)

*Allergy, Asthma & Clinical Immunology* 2019, **15(Suppl 4):**O31

**Introduction:** Hereditary angioedema due to C1-inhibitor deficiency is a rare autosomal dominant disorder affecting the C1-inhibitor gene affecting about 1 in 50,000 individuals. The hallmark of HAE is recurrent angioedema. Autoimmune disorders may complicate genetic C1-inhibitor deficiency.

**Case report:** A 7-year-old boy with a family history of angioedema in his father (diagnosis not established, deceased—glioblastoma multiforme) and C1-inhibitor deficiency in paternal grandmother, developed painless swellings of his face following minor trauma from age 6 years. Type I HAE was diagnosed, C4 0.06 g/l (0.16–0.54) C1-inhibitor 0.069 g/l (0.22–0.38), 0% function.

Two months later he developed spontaneous painful swelling of his right knee without trauma or preceding infection. Examination showed warm swollen right knee with minimal restriction of movement and no deformity. No associated systemic or ocular symptoms. FBC and CRP were normal. Autoantibodies and HLA B27 negative. MRI showed large joint effusion with no structural cause. He was referred to Paediatric Rheumatology where a diagnosis of oligoarticular juvenile idiopathic arthritis was made. He made a good response to aspiration and intra-articular triamcinolone. To date there has been no recurrence of JIA. His HAE is under control.

**Discussion:** HAE I and II may be complicated by autoimmune disorders. Case series report 2–12% of the cohort developing a spectrum of autoimmune disorders. Brickman (JACI 1986,77,749) reported a 6-year-old boy with seronegative non-deforming polyarthritis, IgA deficiency and micrognathia. Our patient is clinically similar except for the single joint involvement. Our patient fulfils the criteria for JIA. We believe JIA in this boy is a complication of HAE and are surprised that he developed autoimmune complication so early in his disorder.

**Consent to publish:** Written, informed consent for publication was obtained from the patient [or parent/guardian for patients under 16]

### O32 Cardiac tamponade following cardiac surgery in type 2 HAE patient

#### Sherif Ghabina^1^, Christos Chamos^1^, Michael Shaw^1^ Kamran Baig^2^, Prakash Punjabi^3^, Suranjith Seniviratne^4^, Jimmy H.C. Gooi^5,*^

##### ^1^Department of Anaesthetics, Guy’s and St Thomas’ Hospital, London, United Kingdom; ^2^Cardiothoracic Surgery, St Thomas’ Hospital, London, United Kingdom; ^3^Cardiothoracic Surgery, Hammersmith Hospital, London, United Kingdom; ^4^Department of Immunology, Royal Free Hospital, London, United Kingdom; ^5^Department of Clinical Immunology, King’s College Hospital, London, United Kingdom

###### **Correspondence:** Jimmy H.C. Gooi (jimmyhc.gooi@googlemail.com)

*Allergy, Asthma & Clinical Immunology* 2019, **15(Suppl 4):**O32

**Introduction:** Invasive and surgical procedures are well known to precipitate angioedema in hereditary angioedema. Cardiac surgery has been successfully performed with a number of prophylactic treatment. We report a patient with HAE who had cardiopulmonary bypass mitral valve replacement surgery with C1-inhibitor prophylaxis complicated by pericardial effusion and cardiac tamponade which was successfully treated.

**Case report:** MK (female, date of birth 03/09/1972) who has been resident in UK since 1991 was diagnosed with Type 2 HAE at age 22 years and on danazol prophylaxis. Sjogren’s syndrome was diagnosed in 2005. She developed carcinoma breast in 2013 which was treated by lumpectomy followed by chemotherapy and radiotherapy and long term tamoxifen. Mitral valve disease was diagnosed in 2014.

Mitral valve replacement surgery was performed in February 2017. She received 1000 units C1-inhibitor before and 1500 units after cardiopulmonary bypass surgery. She was discharged a week later to represent with palpitations and peripheral oedema. Atrial fibrillation, large pericardial effusion and early tamponade was diagnosed. She underwent needle pericardiocentesis under 1000 units C1-inhibitor which was complicated by puncture of the pulmonary artery. Pericardial effusion recurred. Emergency surgical drainage was performed under 1000 units C1-inhibitor before and 1500 units after surgery. Pericardial drain was in situ for 3 days following which she had successful cardioversion under 1000 units C1-inhibitor prophylaxis. The patient who is followed up in another hospital has had exacerbations of angioedema since discharge.

**Discussion:** The patient had a major surgical procedures done in 2 hospitals with no resident immunologist. There is no specific evidence based guidance on the prophylactic treatment of HAE in major complex surgery. The treatment was successful but highlights the need for a better prophylaxis and treatment protocol and multidisciplinary approach in the management of complex invasive procedures in HAE.

**Consent to publish:** Written, informed consent for publication was obtained from the patient [or parent/guardian for patients under 16]

### O33 Clinical characteristics and therapeutic modalities in Polish C1–INH–HAE patients. A pilot cohort study in adults population

#### Katarzyna Piotrowicz-Wojcik^1^, Aldona Juchacz^2^, Krzysztof Kuziemski^3^, Krystyna Obtulowicz^1^, Grzegorz Porebski^1,*^

##### ^1^Department of Clinical and Environmental Allergology, Jagiellonian University Medical College, Krakow, Poland; ^2^Centre of Pulmonology and Thoracic Surgery, Poznan, Poland; ^3^Department of Pulmonology and Allergology, Faculty of Medicine, Medical University of Gdansk, Poland

###### **Correspondence:** Grzegorz Porebski (g.porebski@uj.edu.pl)

*Allergy, Asthma & Clinical Immunology* 2019, **15(Suppl 4):**O33

**Background:** Our study aimed to determine current management approaches and clinical characteristics of Polish population of adults patients with hereditary angioedema due to C1-inhibitor deficiency (C1-INH-HAE).

**Materials and methods:** We undertook a survey of consenting C1-INH-HAE patients with a structured medical interview addressing i.a. patients’ sex, date of first symptoms and diagnosis, frequency and localization of angioedema, medication use for on-demand and prophylactic treatment.

**Results:** Ninety patients, at the mean age of 41.7 years (range 18–77), were available for analysis at the present stage (females: 57, males: 33; HAE type I: 90%, HAE type II: 10%). The mean age at onset of symptoms was 13 years (range 1–32) and the mean delay in diagnosis was 14.8 years (range 0–56). Family history was present in 89.8% of subjects, while de-novo mutation was presumed in 10.2%. Family history of HAE related death, unnecessary surgeries and intubations due to laryngeal attacks were reported in 33%, 11.1% and 4.9% of the patients, respectively. The most common symptoms were (i) peripheral swellings: no attacks, < 12 and ≥ 12 attacks in 16.1%, 59.8%, 24.1% of patients respectively, median: 5, mean: 7.4, range: 0–30 attacks/6 months per patient and (ii) abdominal swellings: no attacks, < 12 and ≥ 12 attacks in 20.1%, 58.6%, 21.3% of patients respectively, median: 4, mean: 6.8, range: 0–30 attacks/6 months per patient. Laryngeal attacks, potentially life threatening, were reported in 28.9% of patients within 6 months before a survey. Additionally, 75.4% of patients reported attacks in multiple localizations at the same time. 61.7% of the patients report self-administration of on demand treatment and 78.3% of them carry emergency treatment when travelling. 8.1% of the patients do not have on demand drugs at home. The others have pdC1-INH (83.5%), icatibant (46.8%), rhC1-INH (8.9%) – percent do not add up to 100% due to patients having simultaneously more than one drug. 19.8% of the patients didn’t use on demand treatment within the last 6 months, the others used pdC1-INH (76.8%), icatibant (49.3%), rhC1-INH (8.7%). Long-term prophylaxis with danazol and/or tranexamic acid were used by 9.3% of the patients within the last 6 months and by 51.5% of the patients, ever.

**Conclusions:** Clinical profile of the investigated patients is similar to other reported populations. Collected data gives further insight into unmet medical needs, e.g. self-administration and may help to identify certain patient groups which require additional focus in the future.

### O34 Treatment of patients with hereditary angioedema with normal C1-inhibitor: evaluation of 295 patients

#### Nyla T.M.L. Fragnan^1^, Stéphanie K.A. Almeida^1^, Camila L. Veronez^2^, Rosemeire N. Constantino–Silva^1^, Sandra M.U. Palma^1^, Adriana S. Moreno^3^, Luiza Karla Arruda^3^, Eli Mansour^4^, Faradiba S. Serpa^5^, Solange R. Valle^6^, Maria Luiza Alonso^6^; Sergio Dortas Jr.^6,16^, Herberto J. Chong Neto^7^, Nelson Rosário^7^, Carolina G.F. Batista^7^, Jane da Silva^8^, Régis A. Campos^9^, Rozana F. Gonçalves^10^, Natasha Ferraroni^11^, Gabriela A.C. Dias^12^, Eliana Toledo^13^; Miguel Piccirillo^14^, Pedro Giavina–Bianchi^15^, Priscila M. Takejima^15^, João B. Pesquero^2^, Anete S. Grumach^1,*^

##### ^1^Faculdade de Medicina do ABC, University Center of Health ABC, Brazil; ^2^UNIFESP – Federal University of São Paulo, Brazil; ^3^USP/FMRP – State University of São Paulo, Faculdade de Medicina de Ribeirão Preto, Brazil; ^4^UNICAMP - State University of Campinas, Brazil; ^5^Santa Casa de Vitória – ES, Brazil; ^6^UFRJ - Federal University of Rio de Janeiro, Brazil; ^7^UFPR - Federal University of Paraná, Brazil; ^8^UFSC - Federal University of Santa Catarina, Brazil; ^9^UFBA - Federal University of Bahia and Private Medical Office in Salvador – BA, Brazil; ^10^Private Medical Office in Belo Horizonte – MG, Brazil; ^11^Private Medical Office in Brasília – DF, Brazil; ^12^UERJ - State University of Rio de Janeiro, Brazil; ^13^FAMERP - Faculdade de Medicina de São José do Rio Preto, Brazil; ^14^Private Medical Office in Londrina – PR; ^15^USP - State University of São Paulo, Brazil; ^16^UNIG - Universidade Iguaçu, Brazil

###### **Correspondence:** Nyla T.M.L. Fragnan (asgrumach@gmail.com)

*Allergy, Asthma & Clinical Immunology* 2019, **15(Suppl 4):**O34

**Background:** Hereditary angioedema with normal C1-inhibitor (HAE-nlC1-INH) is a rare condition and clinical features are like those of HAE with C1-INH deficiency. Hormones have special role as triggering factor. There is no biomarker for diagnosis, requiring a consistent clinical and family history and/or identification of associated mutation (Factor 12, Angiopoietin 1 and Plasminogen).

**Methods:** 304 patients of 101 unrelated families were evaluated as suspected HAE-nlC1-INH. Questionnaires were filled out by 16 Brazilian Centers. HAE-nlC1-INH criteria was fulfilled by 225 patients of 78 families. One of the families (4 members) showed Angiopoietin 1 mutation. Detailed clinical data including therapy were recorded. Genetic mutations were performed for the 3 known HAE-nlC1-INH mutations. This study was approved by the Ethics Committee of Centro Universitario Saude ABC (CAAE:51896015.0.1001.0082).

**Results:** 44/225 (19.5%) were asymptomatic and 181/225 (80.5%) were symptomatic. Out of these, 141/181(77.9%) had F12 mutation, 2/181 (1.1%) had Angiopoetin 1 mutation, 21/181 (11.6%) the mutation was unknown and 17/181 (9.4%) weren’t tested yet (Table 1).

**Table 1:** Clinical data of patients with HAE-nlC1-INH according to the identification of mutation

	HAE-F12			HAE-unknown		
Patients	183			38		
Families	62			15		
Family history	170/183		92.9%	38/38		100%
Sex	153F:30M		83.6%:16.4%	33F:5M		86.8%:13.2%
Onset of symptoms (age)	2–68 (136/141) median = 18.5			2–52 (31/38) median = 16		
Site of edema						
Subcutaneous	84/141		59.6%	26/38		68.4%
Facial	119/141		84.4%	31/38		81.6%
Genital	21/141		14.9%	10/38		26.3%
Abdominal	109/141		77.3%	28/38		73.7%
Tongue	32/141		22.7%	5/38		13.2%
Upper airways	65/141		46.1%	17/38		44.7%
Trigger factors						
Hormones (symptomatic women) (n = 128)						
Oral contraceptives	87/128		68.0%	17/33		51.5%
Pregnancy	25/128		19.5%	6/33		18.2%
Menses	1/128		0.8%	3/33		9.1%
HRT	2/128		1.6%	1/33		3.0%
All symptomatic patients						
Stress	87/141		61.7%	18/38		47.4%
Trauma	70/141		49.6%	16/38		42.1%
Surgery	11/141		7.8%	4/38		10.5%
Dental procedure	21/141		14.9%	5/38		13.2%
Infection	6/141		4.3%	4/38		10.5%
Weather	11/141		7.8%	4/38		10.5%
Exercise	4/141		2.8%	5/38		13.2%

HRT: hormonal reposition therapy

Eight symptomatic patients with F12 mutation (8/141, 5.6%) underwent surgical procedures due to angioedema: laparotomy, 4 (2.8%); laparoscopy, 3 (2.1%); tracheostomy, 1 (0.7%). Among symptomatic patients with unknown mutation, 2 were submitted to laparotomy (2/38; 5.3%). Thirteen (13/181, 7.2%) were admitted in ICU during attacks at least once and 3/181 (1.7%) died due to HAE attacks. Of the symptomatic patients, 38/181 (21%) did not receive any specific treatment. The patients treated (143/181, 79%) were 116/143 (81.1%) with F12 mutation, 25/143 (17.5%) with unknown mutation and 2/143 (1.4%) with Angiopoietin 1 mutation. Among the patients who underwent specific treatment: 45/143 (31.5%) only stopped or modified contraceptives; 32/143 (22.4%) were medicated during the attacks only and 66/143 (46.1%) received long term prophylactic treatment. Short term prophylaxis was performed for 28/143 (19.6%). Tables 3 and 4 refer to drugs used for on-demand treatment and prophylaxis.

**Table 3:** Treatment on demand in patients with HAE-nlC1-INH

	Total (32/143 = 22.3%)		HAE-F12 (24/116 = 19.8%)		HAE-Unknown (8/25 = 28%)	
	n	%	n	%	n	%
Icatibant	7	21.9	3	12.5	4	50.0
C1-inhibitor	3	9.4	1	4.2	2	25.0
Tranexamic acid	25	78.1	21	87.5	4	50.0
Danazol	1	3.1	0		1	12.5

**Table 4:** Medications used for continuous prophylactic treatment by patients with HAE-nlC1-INH

	TOTAL (66/143 = 46.1%)		HAE – F12 (53/116 = 45.7%)		HAE – UNKNOWN (12/25 = 48%)		Angiopoetin 1 (1/2 = 50%)	
	n = 66	%	n = 53	%	n = 12	%	n = 1	%
Tranexamic acid	52	78.8	43	81.1	8	66.7	1	100
Danazol	20	30.3	12	22.6	7	58.3	1	100
Oxandrolone	14	21.2	8	15.1	5	41.7	1	100
Epsilon aminocaproic acid	4	6.1	0		3	25	1	100

**Conclusions:** Our registry represents one of the greatest HAE-nlC1-INH casuistry reported. Clinical manifestations were similar both in F12 or ANGPT1 mutated and unknown mutation HAE-nlC1-INH patients. Most of the patients were symptomatic and almost half of them needed prophylactic treatment.

**Keywords:** Hereditary angioedema; C1-inhibitor; therapy; f12 mutation.

### O35 Long-term prophylaxis with C1-inhibitor concentrate in patients with hereditary angioedema

#### David Loli-Ausejo^1,*^, Ana Entrala^2^, Rosario Cabañas^2^, Jesús Jurado-Palomo^3^, Cristina Mañas–Rueda^1^, Irene Hernández-Martín^1^, Teresa Caballero^2,4^

##### ^1^Department of Allergy, Hospital La Paz, Madrid, Spain; ^2^Department of Allergy, Hospital La Paz Institute for Health Research (IdiPaz), Madrid, Spain; ^3^Department of Allergy, Nuestra Señora del Prado Hospital, Talavera de la Reina, Spain; ^4^Biomedical Research Network on Rare Diseases – U754 (CIBERER), Madrid, Spain

###### **Correspondence:** David Loli-Ausejo (david.loli@hotmail.com)

*Allergy, Asthma & Clinical Immunology* 2019, **15(Suppl 4):**O35

**Objectives/introduction:** Hereditary angioedema due to C1-inhibitor deficiency (C1-INH-HAE) is a rare disease characterized by recurrent attacks of subcutaneous and/or submucous edema. Patients may benefit from long-term prophylaxis (LTP) when attacks are frequent or severe. The objective of this study was to describe the clinical and therapeutic characteristics of patients treated with plasma-derived human C1-inhibitor concentrate (pdhC1INH) as LTP.

**Methods:** A retrospective review of clinical histories of patients with C1-INH-HAE receiving LTP with pdhC1INH was performed (2000–2019).

**Results:** Fourteen out of 168 patients with C1-INH-HAE [13/165 type I, 1/3 type II; 12/86 (14.0%) women, 2/82 (2.4%) men] had received LTP with pdhC1INH. A male patient did not continue follow-up in our centers. The median age at onset of LTP with pdhC1INH was 37 years (Q1–Q3:33.5–40.0).

The reason for initiating LTP with pdhC1INH was an increase in the frequency of angioedema attacks, either by contraindication for the administration of conventional LTP with attenuated androgens (AAs) or tranexamic acid (TXA) (pregnancy/lactation 7, cancer 2), inefficiency of conventional LTP (AAs, TXA) (3) or unacceptable AA adverse effects (2).

The most frequent initial dose was intravenous (IV) 1000U pdhC1INH every 5 days. The most frequent final dose was IV 1000U pdhC1INH every 2 days. The 14 patients started with IV pdhC1INH and 1 switched from IV to subcutaneous pdhC1INH (off-label use in Spain). The median time to optimize the dose was 14 months. The median total administration time was 21 months (Q1–Q3:9–73). Total administration time was up to 14 years.

LTP with pdhC1INH was initiated with Berinert^®^ (CSL-Behring, Marburg, Germany) in 12 patients. Two patients are taking currently Berinert^®^ and 5 patients Cinryze^®^ (Shire, now part of Takeda, Zug, Switzerland).

A central venous access was placed initially for home self-infusion in two patients. One of these patients developed sepsis secondary to central venous access contamination. Another patient suffered from headache secondary to fast pdhC1INH infusion and then home self-infusion with a pump was started with good tolerance. This patient also had facial malar erythema with Cinryze^®^ but tolerated Berinert^®^. No viral seroconversion was detected. One patient died due to a breast cancer unrelated to pdhC1INH. No other side-effects were observed.

The patient or a relative were trained in IV pdhC1INH self-administration in all the cases.

**Conclusion:** pdhC1INH LTP was more frequently needed by women, proved to be an effective, safe and well tolerated alternative in patients with contraindications for administration of conventional LTP, including pregnancy and lactation.

### O36 The needs of individually tailored prophylaxis with C1-INH concentrate in pediatric patients with hereditary angioedema (HAE) – real life data from 6 pediatric patients

#### Inmaculada Martinez Saguer, Carmen Escuriola Ettingshausen, Zeynep Gutowski, Richard Linde

##### Haemophilia Centre Rhine Main, Frankfurt-Moerfelden, Germany

###### **Correspondence:** Inmaculada Martinez Saguer (inmaculada.martinez@hzrm.de)

*Allergy, Asthma & Clinical Immunology* 2019, **15(Suppl 4):**O36

**Background:** Hereditary angioedema due to C1-inhibitor deficiency (HAE-C1-INH) is a rare autosomal dominant inherited disease. The recurrent swelling attacks such as subcutaneous edema and colic-like abdominal pain negatively affect quality of life (QoL). Laryngeal edema is rare, but life-threatening if untreated. C1-INH is currently approved for prophylaxis to routinely prevent attacks in patients aged ≥ 6 (EU) and ≥ 12 years (US). Real life data in nine pediatric patients with HAE who received C1-INH concentrate for the routine prevention of angioedema attacks was documented and followed up.

**Methods:** After giving informed consent the following data was collected and analyzed from patient’s diaries and records 1 year before onset of and after introduction of prophylactic treatment: age at first manifestation and diagnosis, age at first treatment, frequency and location of attacks, prophylactic respectively on-demand therapy regimen. Initial standard prophylactic treatment (SP) consisted of 1000 U C1-INH, (bw > 40 kg) or 500 U C1-INH (bw < 40 kg) every 3–4 days i.v. and was intensified (individualized prophylaxis—IP) in case of > 2 breakthrough attacks per month. In 2 patients the prophylactic regimen had to be intensified to every 2 days regimen.

**Results:** Six patients (3 male/3 female) aged 4.5–17.2 years with HAE-C1-INH type I were enrolled. Attacks before onset of prophylaxis occurred 3–11 times/month and affected mainly abdomen and extremities but also face; a history of laryngeal attacks was reported in four patients. SP resulted in zero break-through attacks in 4/6 patients during a prophylaxis period of 6 –41 months. Two patients still presented > 2 break-through attacks/month. Consecutive IP resulted in zero break-through attacks in these patients as well during 20–38 months.

**Conclusions:** These real life data strongly indicate that prophylaxis regimes should be tailored according to the individual patient’s needs in order to achieve favorable results.

## Posters

### P01 A case of hereditary angioedema associated with rheumatoid arthritis: treatment challenges

#### Sladjana Andrejevic^1,3,*^, Radovan Mijanovic^1^, Mirjana Sefik-Bukilica^2,3^

##### Clinic of Allergy and Immunology, Clinical Center of Serbia, Belgrade, Serbia; ^2^ Institute of Rheumatology, Belgrade, Serbia; ^3^ School of Medicine, University of Belgrade, Belgrade, Serbia

###### **Correspondence:** Sladjana Andrejevic (sandrejevic@yahoo.com)

*Allergy, Asthma & Clinical Immunology* 2019, **15(Suppl 4):**P01

**Background:** Patients with hereditary angioedema (HAE) have an increased incidence of autoimmune diseases. such as systemic lupus erythematosus, rheumatoid arthritis (RA), autoimmune thyroiditis, glomerulonephritis, etc.

We here present a case of severe RA in a female patient with HAE and the impact of specific RA treatments on the severity of HAE.

**Case report:** 34-years old female patient in whom diagnosis of HAE was established after diagnosing of her uncle and mother. She is a member of the largest HAE family in Serbia, in which all offspring inherited the disease. Disease-causing missense mutation in *SERPING1* gene (Pro377Ser-c.1195C > T on exon 7) was identified in all family members. The first presentation of HAE was laryngeal angioedema at the age of 24. Since then moderate and severe attacks occurred with the frequency of 1–2 per year. She was diagnosed with seropositive erosive RA at the age of 20 and initially treated with prednisolone and metotrexate. Since she did not go into remission the treatment with tocilizumab (humanized recombinant anti-interleukin-6 receptor antibody) was initiated two years later. She was treated with tocilizumab 480 mg i.v. monthly, daily oral methylprednisolone 4 mg and alfacalcidiol between 22 to 33 years of age with good clinical response. For arterial hypertension since the age of 26 treatment consisted of amlodipine and bisoprolol. As she planned to become pregnant therapy was changed to methyldopa and etanercept (tumor necrosis factor inhibitor) at a dose of 50 mg s.c. weekly. Shortly after the introduction of new therapy she experienced flare-ups of RA and severe HAE attacks 1–3 per month including laryngeal edema. When pregnancy was confirmed etanercept was discontinued. Therapy with methyldopa was continued. She miscarried when she was 6 weeks pregnant at October 2018. Since etanercept was stopped, she has not experienced any severe HAE attack. Tocilizumab treatment was reintroduced in January 2019 and proved to be effective.

There is no literature data of tocilizumab usage in HAE. Etanercept was used in two patients with psoriatic arthritis and angioedema (one with HAE, the other with AAE) with favorable effects. Two cases of severe angioedema, were described in patients treated with etanercept. (one with RA and one with refractory adult-onset Still’s disease).

**Conclusion:** To the best of our knowledge, this is the first case of successful treatment of RA with tocilizumab in HAE patient. Moreover, it’s needed to further evaluate the effect of etanercept on possible worsening of HAE.

**Consent to publish:** Written, informed consent for publication was obtained from the patient [or parent/guardian for patients under 16]

### P02 Acute treatment of pregnant women with hereditary angioedema attacks: administration of recombinant human C1 esterase inhibitor

#### Jonathan A. Bernstein^1,*^, Dumitru Moldovan^2^, Roman Hakl^3^, Grzegorz Porebski^4^, Kimberly Poarch^5^, William R. Lumry^6^, Anurag Relan^7^

##### ^1^College of Medicine, University of Cincinnati, Cincinnati, Ohio, United States; ^2^MediQuest Clinical Research, Sangeorgiu de Mures, Romania; ^3^St Anne’s University Hospital in Brno, Faculty of Medicine; Masaryk University, Brno, Czech Republic; ^4^Department of Clinical and Environmental Allergology, Jagiellonian University Medical College, Krakow, Poland; ^5^Allergy & Asthma Specialists of Dallas, Dallas, TX, United States; ^6^University of Texas Southwestern Medical School, Dallas, Texas, and Allergy & Asthma Specialists of Dallas, Dallas, TX, United States; ^7^Pharming Healthcare, Inc., Bridgewater, NJ, United States

###### **Correspondence:** Jonathan A. Bernstein (bernstja@ucmail.uc.edu)

*Allergy, Asthma & Clinical Immunology* 2019, **15(Suppl 4):**P02

**Background:** Changes in hormone levels during pregnancy may exacerbate hereditary angioedema (HAE) attacks. Recombinant human C1 esterase inhibitor (rhC1-INH) is safe and effective as acute treatment of HAE attacks in adults/adolescents and has also been investigated in a phase 2 trial as prophylaxis in individuals with frequent HAE attacks. Limited data are available in pregnant women. The objective of the current study was to characterise clinical outcomes of pregnant patients with HAE treated with rhC1-INH.

**Materials and methods:** Pregnant women with HAE who received rhC1-INH were reported as a part of pharmacovigilance/clinical trial activities and followed during pregnancy and until delivery. Adverse events/neonatal outcomes were reported.

**Results:** Fourteen pregnant women (aged 17–37 years) with HAE were treated with rhC1-INH (range, 2100–4200 IU) during pregnancy. Most women were treated with a single dose of rhC1-INH; one woman, treated with rhC1-INH 3150 IU, required a second dose. Two women had 10 life-threatening upper airway attacks combined; all HAE attacks responded to rhC1-INH within 2–4 h post-treatment. There were no adverse events considered related to rhC1-INH. All 14 pregnancies resulted in delivery of full-term healthy babies; foetal distress and congenital abnormalities were not observed.

**Conclusions:** Treatment with rhC1-INH in pregnant women with HAE was generally safe and well tolerated. All 14 women delivered healthy babies at full-term without complications.

Supported by Pharming Healthcare Inc.

### P03 Tranexamic acid plus sodium bemiparin as long term prophylaxis in a patient with FXII-HAE during pregnancy: a case report

#### Irene Hernández-Martín^1,*^, David Loli-Ausejo^1^, Rosario Cabañas^2^, Ana Entrala^2^, Mar Gutiérrez–Alvariño^3^, Nuria Martínez-Sánchez^4^, Teresa Caballero^2,5^

##### ^1^Allergy Department, Hospital La Paz, Madrid, Spain; ^2^Allergy Department, Hospital La Paz Institute for Health Research, IdiPaz, Madrid, Spain; ^3^Haematology Department, Hospital La Paz, Madrid, Spain; ^4^Gynecological and Obstetric Department, Hospital La Paz, Madrid, Spain; ^5^Biomedical Research Network on Rare Diseases – U754 (CIBERER), Madrid, Spain

###### **Correspondence:** Irene Hernández-Martín (irenhermar@gmail.com)

*Allergy, Asthma & Clinical Immunology* 2019, **15(Suppl 4):**P03

**Background:** Hereditary angioedema (HAE) is characterized by recurrent attacks of severe swelling with involvement of multiple organs, which are induced by genetic mutations that result in increased bradykinin levels. Patients with a mutation in *F12* gene (FXII-HAE) especially show worsening of symptoms under hyperestrogenic conditions, such as pregnancy. Therapy for HAE is limited during pregnancy, delivery, and postpartum (1). Plasma-derived C1-INH (pdC1-INH) concentrate is the election treatment recommended during these periods (2).

**Materials and methods:** We present the evolution and management of repeated angioedema attacks during pregnancy in a woman with HAE, with normal levels and function of C1-INH and a mis-sense mutation in *F12* gene. She had been diagnosed in 2012, during her first pregnancy, due to a severe facial angioedema attack. She had previously suffered from several angioedema attacks after starting oral contraceptive hormone treatment.

**Results:** Her second pregnancy was marked by recurrent episodes of facial angioedema. She was on sickness leave since the 7th week of pregnancy and was followed up in the high risk pregnancy consultation. During the first pregnancy trimester the patient was treated several times with intravenous plasma derived human C1-INH concentrate (pdhC1INH) (Berinert^®^, CSL-Behring, Marburg, Germany) in the Emergency Room, sometimes needing two or three 1,500U doses to resolve the attack. pdhC1INH was discarded as LTP due to lack of efficacy in the treatment of acute angioedema attacks. The patient was evaluated by a haematologist before starting oral tranexamic acid (TXA), who recommended co-treatment with sodium bemiparin 7,500U daily and hematologic controls every few weeks. She was asymptomatic for 4 months immediately after starting oral TXA 500 mg every 8 h, having just 3 mild attacks in the last 3 months of pregnancy and treating 2 of them with 1,500U of pdhC1INH with acceptable response. A caesarean delivery was scheduled because of a prior caesarean and short term prophylaxis with IV pdhC1INH (1,000U) was administrated. She discontinued TXA the night before the delivery, which went through without incidences, and a healthy female baby was born. A tubal ligation was also performed. She was breastfeeding and had to continue the treatment with heparin for 6 weeks after the delivery. No new angioedema attacks were observed postdelivery.

A *F12* gene mutation was discarded in the newborn.

**Conclusions:** In our patient, a successful management of the angioedema attacks during pregnancy was carried out with TXA and anticoagulant treatment. No side effects were observed.

**References**

1. Caballero T, Canabal J, Rivero-Paparoni D, Cabanas R. Management of hereditary angioedema in pregnant women: a review. Int J Womens Health. 2014 Sep 9;6:839-48.
2. García JFB, Takejima P, Veronez CL, Aun MV, Motta AA, Kalil J, Giavina-Bianchi P. Use of pdC1-INH concentrate for long-term prophylaxis during pregnancy in hereditary angioedema with normal C1-INH. J Allergy ClinImmunolPract. 2018; Aug 6 (4): 1406-1408.
3. Milingos DS, Madhuvrata P, Dean J, Shetty A, Campbell DM. Hereditary angioedema and pregnancy: successful management of recurrent and frequent attacks of angioedema with C1-inhibitor concentrate, danazol and tranexamic acid – a case report. Obstetric Medicine. 2009. 2(3), 123–125.
4. Zuraw BL, Bork K, Binkley KE, et al. Hereditary angioedema with normal C1-inhibitor function: consensus of an international expert panel. Allergy Asthma Proc. 2012; 33 Suppl 1:S145–S156.
5. Caballero T, Farkas H, Bouillet L, et al. International consensus and practical guidelines on the gynecologic and obstetric management of female patients with hereditary angioedema caused by C1-inhibitor deficiency. J Allergy ClinImmunol. 2012; 129(2):308–320.

### P04 Metabolic complications of late diagnosis in hereditary angioedema

#### Natalia Vélez-Tirado^1,*^, Alberto Contreras-Verduzco^1^, Sandra Nieto–Martinez^2^

##### ^1^Pediatric Allergy Department, Instituto Nacional de Pediatría, Mexico City, México; ^2^Genetic of Nutrition Unit, Instituto Nacional de Pediatría, Mexico City, México

###### **Correspondence:**Natalia Vélez-Tirado (natalia_velezt5@hotmail.com)

*Allergy, Asthma & Clinical Immunology* 2019, **15(Suppl 4):**P04

**Background:** Type I hereditary angioedema (type I HAE) is a rare genetic disease characterized by episodes of subcutaneous or mucosal edema, without urticaria or pruritus, that can be fatal due to laryngeal swelling. In Mexico the diagnosis is usually delayed due to lack of clinical suspicion in first-contact physicians. The most frequent previous diagnosis is chronic urticaria, whereby patients receive recurrent and prolonged treatment with antihistamines and corticosteroids. Metabolic syndrome (MS) is traditionally an adult disease, however its prevalence in pediatrics is increasing; is characterized by a combination of dyslipidemia, abnormal glucose regulation, central adiposity and hypertension. It has defined diagnostic criteria for children between 10 and 16 years (Table 1), in those under 10 years of age it is not possible to make the diagnosis due to lack of consensus. MS is associated with a higher risk of developing cardiovascular disease and type 2 diabetes (T2D). We present the case of 2 patients with type I HAE who met criteria for metabolic syndrome. The third patient had a diagnosis of Cushing syndrome (Table 2).

**Table 1:** Definition of metabolic syndrome in children and adolescents by the International Diabetes Federation

Parameters	10–16 years
Waist circumference	≥ 90^th^ percentile*
Number of abnormalities	≥ 2 of the following
Triglycerides	≥ 150 mg/dl
High density protein (HDL) colesterol	< 40 mg/dl
Blood pressure	Either
Systolic	> 130 mmHg
Diastolic	≥ 85 mmHg
Fasting glucose	≥ 100 mg/dl

*Fernandez JR, et al. Waist circumference percentiles in nationally representative samples of African-American, European-American, and Mexican–American children and adolescents. J Pediatr 2004; 145:439

**Table 2:** Metabolic abnormalities

Parameters	Patient 1	Patient 2	Patient 3
Age	12 years	14 years	7 years
Weight	60 kg	74 kg	40
BMI	26.3 (P97)	27.9 (P99)	24 (P99)
Waist circumference	89 cm	102 cm	72 cm
Triglycerides	162 mg/dl	216 mg/dl	132 mg/dl
High density protein (HDL)	33.3 mg/dl	36.1 mg/dl	41
Blood pressure			
Systolic	125 (P > 95)	120 (P50)	100 (P50)
Diastolic	75 (P90-95)	84 (P99)	60 (P50)
Fasting glucose	93 mg/dl	106 mg/dl	83 mg/dl

**Case report:** We present the case of 3 patients with type I HAE who before diagnosis received multiple cycles of systemic corticosteroids as treatment for angioedema, emesis and abdominal pain. Two patients met obesity criteria by body mass index (BMI); within their diagnostic approach multiple metabolic disorders were evidenced: hypertriglyceridemia, low high density protein (HDL) cholesterol, increased abdominal circumference and hypertension for age, fulfilling criteria for metabolic syndrome. The third patient was obese, with full moon face, hirsutism, dorsal fat pad and total cholesterol level in 232 mg/dl, diagnosis of Cushing syndrome was made.

**Conclusion:** Metabolic syndrome, obesity and cushing syndrome are serious complications of the excessive use of steroids in patients with type I HAE. As allergologist we must intervene quickly and aggressively seeking to avoid the increased in cardiovascular risk and type 2 diabetes in this group of patients.

**Consent to publish:** Informed consent to publish has been obtained from this patients parents.

### P05 HAE Patients in Ukraine: frequency and localization of attacks in 2018

#### Liudmyla Zabrodska

##### Centre of Allergic Diseases of Upper Airways, Institute of Otolaryngology by Name of O.S. Kolomiychenko of the National Academy of Medical Sciences of Ukraine, Kyiv, Ukraine

###### **Correspondence:** Liudmyla Zabrodska (zabrodskalv@gmail.com)

*Allergy, Asthma & Clinical Immunology* 2019, **15(Suppl 4):**P05

**Background:** Ukraine has HAE 37 patients: 5 children (mean age 9 y.o, age range 2 to 16 y.o.), 2 men and 20 women (mean age 41 y.o., age range 21 to 68 y.o.)). The patients have no access to the contemporary treatment, but the situation is expected to change in 2019.

**Objective:** this study focuses on the anamnesis, localization, severity and frequency of HAE attacks.

**Method:** analysis of the patients’ answers to the questionnaire about time of the first attack, date of HAE established diagnosis, frequency, severity, most frequent localization of the attacks, triggers and family anamnesis.

**Results:** 30 patients indicated that they had attacks between 0 and 5 years old, 5 patients developed attacks in the puberty, 2 patients started suffering after 30 years old. Out of these 30 patients 21 adult patient and 1 boy has severe throat and abdominal attacks that require hospitalization. The boy was diagnosed at 5 years old with no family history (parents have had no blood tests yet).

20 patients consider psychological and physical stress, respiratory viral infections, physical traumas, hypothermia, overheat as triggers to develop an HAE attack. Taking ACE inhibitors is a trigger as well: an HAE patient took ACE inhibitor (captopril) to decrease the blood pressure, and in 4 h he developed extremely severe abdominal attack, hypotony and loss of consciousness. In 6 h of intensive care (FFP and other treatment) he was back to normal.

5 adults (3 men and 2 women) had upper airway attacks in 2018. They were hospitalized and transfused FFP [fresh frozen plasma]. FFP was effective in all cases, the edema and pain decreased within one hour and in 4–6 h the patients were transferred from intensive to a usual unit. Yet, after the FFP 2 patients developed rash and scleral icterus (probably due to not sufficient plasma purification) that went back to normal in 3–5 days of antiallergic and detoxication therapy. In Ukraine, FFP is also transfused upon a severe abdominal attack when a patient is in intensive care for an average of 3 days. The attacks of the peripherals are not treated because they are not life threatening.

As shown in Table 1, during 2018 men and women had HAE attacks mainly of peripherals and abdominal parts. Men had twice more facial and upper airway attacks. When compared with others, the patients that suffer HAE from their childhood have more severe and frequent attacks.

**Table 1:** Localization and frequency of HAE attacks in 2018

Edema localization	Man	Women
Peripheral	30	40
Face	15	7
Abdominal	25	27
Upper airways	5	1

### P06 Hereditary angioedema with C1-inhibitor deficiency (HAE-C1-INH) in childhood and adolescence

#### Emel Aygören-Pürsün, Schimalee Andarawewa

##### Department for Children and Adolescents, Angioedema Centre, University Hospital Frankfurt, Goethe University, Frankfurt, Germany

###### **Correspondence:**Emel Aygören-Pürsün (aygoeren@em.uni-frankfurt.de)

*Allergy, Asthma & Clinical Immunology* 2019, **15(Suppl 4):**P06

**Background:** Hereditary angioedema with C1- inhibitor deficiency (HAE-C1-INH) is a rare genetic disease with an estimated prevalence of 1.5 in 100.000. Clinical symptoms usually start in childhood. The mean disease onset occurs at age 7–11. In this study we report data on the clinical course in a cohort of individuals with HAE-C1-INH aged 18 years and younger.

**Methods:** 130 patients with HAE-C1-INH aged ≤ 18 years were followed at the HAE Comprehensive Care Center at the University Hospital Frankfurt, Germany. Data on the clinical course of the disease including the age at disease onset, location of HAE attacks at symptom onset and frequency of attacks in the further course of the disease documented in the patients´ records and symptom diaries were analyzed. The Kaplan-Meyer method was applied for determination of the risk of disease onset at each age until age 18. The study was approved by the Ethics Committee of the University Hospital Frankfurt, Germany.

**Results:** In 75 of 129 (58.1%) patients with a known date of the first manifestation, an onset of disease during the observation period was documented. The median age at onset of disease in this cohort was 4.3 (0.1 to 16.8) years. However, 54 patients were still asymptomatic. According to the Kaplan-Meyer method, the risk of disease onset until age 18 years was 86.3%.

The sites of the initial angioedema episode were mainly extremities (43%), gastrointestinal tract (22%) and face (21%). Laryngeal edema was the initial presentation in two patients (2.6%).

In the year following the onset of disease the mean attack frequency was 2.21 attacks/year, as opposed to a mean of 3.97 attacks in the subsequent year.

At the age of 11, the mean attack frequency was 17.51 per year, followed by a peak frequency of 38.89 attacks per year at the age of 14, and 26 attacks per year at age 18 years.

**Conclusions:** The clinical manifestation of HAE-C1-INH begins in childhood mainly, the vast majority occurs already until age 18. Even in childhood and adolescence, HAE-C1-INH is a many facetted disease. All sites of attacks that are involved in manifestation of HAE in adult patients, including the laryngeal region, are represented in the initial manifestation already, although with a tendency towards involvement of extremities. In total, attack frequency increases with age from the onset of disease until the adult age is reached.

### P07 Higher annual rate of angioedema attacks in HAE-C1-INH patients above the age of 65 compared to patients aged 18 to 64 years

#### Emel Aygören-Pürsün, Schimalee Andarawewa

##### Department for Children and Adolescents, Angioedema Centre, University Hospital Frankfurt, Goethe University, Frankfurt, Germany

###### **Correspondence:** Emel Aygören-Pürsün (aygoeren@em.uni-frankfurt.de)

*Allergy, Asthma & Clinical Immunology* 2019, **15(Suppl 4):**P07

**Background:** Hereditary angioedema with C1- inhibitor deficiency (HAE-C1-INH) is a rare genetic disease that usually starts to manifest in childhood. Almost all adult patients with HAE-C1-INH who are under clinical care are symptomatic. Data on the course of the attack frequency with increasing age in adulthood are scarce. In this cross-sectional study we report data on the annual attack rate in patients over 65 years of age compared to the attack rate in patients at the age of 18 to 64.

**Methods:** Attack characteristics in 147 adult patients with HAE-C1-INH followed at the HAE Comprehensive Care Center at the University Hospital Frankfurt, Germany, were investigated. Patients on exclusive on demand therapy were included. The main exclusion criteria were long-term prophylaxis or pregnancy during the investigational period. After approval by the Ethics Committee of the University Hospital Frankfurt, Germany, data on the clinical course of the disease including frequency and sites of HAE attacks, triggering factors and therapy documented in the patients´ records and symptom diaries were analyzed. The frequency of HAE- attacks in the entirely documented previous year was assessed in two age groups of patients, age ≥ 65 years (47) and age 18 to 64 (100). To analyze the effect of age in more detail, a subset was analyzed further: male and female patients aged 18–30 (28), 31–50 (49) and 51–64 (23).

**Results:** The mean annual rate of angioedema attacks was higher in patients aged 65 or more versus patients aged 18 to 64 years (37.7 vs 31.5 attacks per year, p = 0.000). The mean annual attack frequency in the subsets of patients aged 18–30 years and aged 31–50 years was 28.1 per year and 29.9 per year (p = 0.000). The highest annual number of attacks was found in patients aged 51 to 64 (39.5 per year) (p = 0.000).

**Conclusions:** In this cross-sectional study, patients with HAE-C1-INH aged 65 years or more had more attacks than younger adults. Apart from more diligence in the documentation of attacks or possible selection of more severe cases in the older patient population, this may be an indication of an individual increase of the attack rate over a life-time.

### P08 A questionnaire survey study to determine association of dental hygiene practices in hereditary angioedema subjects with the incidence of post-procedural angioedema attacks

#### Umesh Singh^1^, Aleena Banerji^2^, Paula Busse^3^, Sandra Christiansen^4^, Timothy Craig^5^, Mark Davis-Lorton^6^, Joshua Jacobs^7^, Henry Li^8^, William Lumry^9^, Marc Riedl^4^, Raffi Tachdjian^10^, James Wedner^11^, Bruce Zuraw^4^, Jonathan Bernstein^1,*^

##### ^1^Division of Immunology/Allergy, Dept. of Internal Medicine, University of Cincinnati College of Medicine, Cincinnati, OH, United States; ^2^Division of Rheumatology, Department of Allergy & Immunology, Massachusetts General Hospital, Boston, MA, United States; ^3^Division of Clinical Immunology, Mount Sinai, New York, NY, United States; ^4^Divison of Rheumatology, Allergy & Immunology University of California, San Diego, La Jolla, CA, United States; ^5^Department of Medicine and Pediatrics, Penn State University, Hershey, PA, United States; ^6^Department of Medicine, Winthrop University Hospital, Mineola, NY, United States; ^7^Allergy and Asthma Clinical Research, Inc., Walnut Creek, CA, United States; ^8^Institute for Asthma and Allergy, PC, Chevy Chase, MD, United States; ^9^Allergy and Asthma Research Associates Research Center, Dallas, TX, United States; ^10^Allergy and Immunology, UCLA Medical Center, Santa Monica, United States; ^11^Allergy and Immunology, Washington University School of Medicine, United States

###### **Correspondence:** Umesh Singh (bernstja@ucmail.uc.edu)

*Allergy, Asthma & Clinical Immunology* 2019, **15(Suppl 4):**P08

**Rationale:** Dental hygiene behaviors and practices in HAE subjects, (i.e., the frequency of routine dental care visits and use of dental hygiene products) may be influenced by severe life-threatening episodes triggered by routine professional dental procedures. We utilized a questionnaire to analyze perceptions by HAE subjects about personal dental care practices compared to non-HAE (control) populations.

**Methods:** A self-reported questionnaire linked to REDCap^®^ server was distributed to gather information on differences in dental care perceptions and behaviors in HAE (n = 250) and non-HAE populations (n = 256) matched by age, gender, race, and ethnicity. Chi square analyses and a generalized linear model (SAS) were used to determine the significance of association between several parameters related to dental care beliefs and utilization of dental care products, using the questionnaire responses from both groups.

**Results:** He frequency of routine dentist visits did not differ significantly between groups, but among the HAE group it was significantly less in subjects with previous post-procedure angioedema (AE) attacks. Interestingly, antibacterial toothpaste usage was higher among HAE subjects (9.6% vs. 4.3%, p = 0.02). The odds of using antibacterial toothpaste was significantly higher among HAE subjects concerned about AE attacks and actually experiencing attacks than those not concerned or experiencing such attacks (OR: 3.3 [1.1, 9.7], p = 0.03).

**Conclusion:** Experiencing angioedema attacks after prior dental procedures was the most significant determinant in HAE subjects resulting in less frequent routine dentist visits and preference for using anti-bacterial toothpaste.

### P09 The relationship between disease activity and quality of life – a first–time survey in hereditary angioedema

#### Bettina Ignácz^*^, Rebeka Tóháti, Kinga Viktória Kőhalmi, Henriette Farkas

##### Hungarian Angioedema Reference Center, 3^rd^ Department of Internal Medicine, Semmelweis University, Budapest, Hungary

###### **Correspondence:** Bettina Ignácz (ignaczbethi@gmail.com)

*Allergy, Asthma & Clinical Immunology* 2019, **15(Suppl 4):**P09

**Objective:** To assess the quality of life in relation to the number of angioedema episodes and complement parameters in patients with hereditary angioedema resulting from C1-inhibitor deficiency (C1-INH-HAE) followed up at the National Angioedema Reference Center.

**Materials and methods:** During the period from 2016 to 2018, altogether 125 C1-INH-HAE patients (53 males and 72 females) were enrolled, who completed the Angioedema Quality of Life (AE-QoL) questionnaire on occasion of their annual follow-up visit (95 in 2016, 97 in 2017, and 94 in 2018). The findings from the survey were subjected to statistical analysis in order to establish whether the AE-QoL total score is correlated with the annual number of HAE episodes, or with the levels of complement parameters (classical total complement, C3, C4, C1-INH concentration, and C1-INH functional activity) measured in blood samples obtained upon completion of the questionnaire.

**Results:** We found a significant positive correlation between AE-QoL total score and the annual number of HAE attacks in each year of the survey (2016: *p *< 0.0001, r = 0.41; 2017: *p *< 0.0001, r = 0.51, 2018: *p *< 0.0001, r = 0.51). However, a significant relationship could not be established in any of these years between AE-QoL total score and the level of classical total complement, C3, C4, and C1-INH functional activity.

**Conclusion:** AE-QoL is a valuable tool for the assessment of quality of life in C1-INH-HAE patients, because our findings established AE-QoL as a good indicator of disease severity. This makes us to believe that the patients’ quality of life can be reliably assessed based on the annual number of HAE episodes. However, when AE-QoL reflects a greatly reduced quality of life, but the number of HAE attacks is low, individualized assessment is necessary to develop an appropriate therapeutic strategy.

*This study was supported by OTKA K124557*

### P10 Are HAE patients able to distinguish prodromes from attacks, and are they correlated?

#### Iris Leibovich-Nassi^1,2,*^, Hava Golander^1^, Raz Somech^3^, Dov Har-Even^4^, Avner Reshef^2^

##### ^1^Department of Nursing, Sackler school of Medicine, Tel Aviv University, Israel; ^2^Barzilai University Medical Center, Ashkelon, Israel; ^3^Safra Pediatric Medical Center, Sheba Medical Center, Ramat Gan, Israel; ^4^Bar-Ilan University, Ramat Gan, Israel

###### **Correspondence:** Iris Leibovich-Nassi (irisl@bmc.gov.il)

*Allergy, Asthma & Clinical Immunology* 2019, **15(Suppl 4):**P10

**Background:** Prodromes of Hereditary Angioedema (HAE) are frequently reported in close association with the swelling attacks. Since attacks usually follow prodromes in a close proximity, patients may not be able to tell the difference between the two events. For practical purposes, especially self-administration and timing of treatments, it is important to distinguish prodromes from attacks. We sought to investigate the differences and correlations between prodromes and attacks, by using a new validated PRO instrument.

**Methods:** We designed and tested a questionnaire-based PRO instrument for the evaluation of HAE prodromes and attacks. The high internal content validity and reliability (Cronbach’s α = .70 to .96) render it suitable for studying clinical expressions of HAE. A cohort of 66 HAE patients completed a questionnaire, developed specifically to address their most recent experience with prodromes and attacks. It incorporates five ‘clusters’ of body systems: limbs, abdomen, face, laryngeal, genitalia. This instrument was powered to evaluate if they could distinguish between five dimensions of prodromes and subsequent attacks. Items included: location, pain, intensity, impairment and dysfunction. Patients were asked to grade their experience in both events on a Visual Analog Scale (Likert) of 0–10 cm.

**Results:** One-way MANOVA with repeated measurements, on all dimensions, showed significant differences between the clinical dimensions of prodromes and attacks, in all body clusters [F (4, 56) = 45.7, P < .001, Eta^2^ = .77]. Patients could distinguish between the two and consider them separately, but associated. Additionally, one-way ANOVA for each dimension demonstrated very high differences. For example, differences in abdominal pain [F (1, 59) = 104.1, P < .001, Eta^2^ = .64], in the limbs [F (1, 64) = 57.2, P < .001, Eta^2^ = .47], and less in the face [F (4, 60) = 30.7, P < .001, Eta^2^ = .33]. Association between the events, analyzed by Pearson’s coefficient, showed high statistical correlation (i.e. abdominal pain r = .45, p < .001, laryngeal pain r = .46, p > .001, facial pain r = .36, p < .01). Therefore, we could demonstrate that higher intensity of a prodrome was followed by higher intensity of an attack. Collectively, the intensity, severity, impairment and loss of functionality were much lower in the prodromes than in the attacks, particularly in the skin and abdomen clusters.

**Conclusions:** By using HAE-specific instrument, we found that patients could identify the prodromes, and describe them accurately by using the questionnaire items. We demonstrate that prodromes and attacks in various locations are distinguishable but associated.

### P11 Psychological processes in the adaptation to disease in adults with hereditary angioedema due to C1-inhibitor deficiency: a pilot study from Italian referral centers

#### Assunta Maiello^1,*^, Livia Savarese^1^, Maria Bova^2^, Ilaria Mormile^3^, Angelica Petraroli^2^, Riccardo Senter^4^, Mauro Cancian^4^, Arturo Genovese^2^, Maria Francesca Freda^1^

##### ^1^Department of Humanities, University of Naples “Federico II”, Naples, Italy; ^2^Department of Translational Medical Sciences and Center for Basic and Clinical Immunology Research (CISI), University of Naples Federico II, WAO Center of Excellence, Naples, Italy; ^3^Post-Graduate Program in Clinical Immunology and Allergy, University of Naples Federico II, Italy; ^4^Department of Medicine, University of Padua, Padua, Italy

###### **Correspondence:** Maria Bova (maielloassunta@gmail.com)

*Allergy, Asthma & Clinical Immunology* 2019, **15(Suppl 4):**P11

**Background:** In a previous research with pediatric C1-inhibitor Angioedema (C1-INH-HAE) patients we found higher levels of perceived stress and alexithymia, evaluated by self-report scales, compared to the normative validation samples.

**Objectives:** The aim of this study is to evaluate the relation between psychological processes and: a) severity of the disease; b) patient’s adaptation to it, in a sample of adults with C1-INH-HAE. In particular, we evaluated the processes of emotion regulation, alexithymia, perception of stress and patient’s health engagement.

**Method:** Within a broader mixed-method research design in clinical health psychology, n. 60 people affected by HAE from the main Italian referral centers and n. 30 controls – affected from different chronic conditions- will be administered the following psychometric scales: Toronto Alexithymia Scale (TAS-20- Taylor, 2004), Emotion Regulation Questionnaire (ERQ, Gross e John, 2003), Perceived Stress Scale (PSS, Cohen, and Williamson, 1988), Patient Health Engagement Scale (PHE-S, Graffigna et al., 2015).

**Results:** Preliminary results on 19 subjects are reported. 79% of the sample are females. The mean age is 43.95 (± 10.47) years and the mean of the years elapsed from the diagnosis is 14.64(± 14.42). The levels of perceived stress are sensibly higher (20.26(± 7.5) than the normative sample (12.1(± 5.9)). Alexithymia and Emotion Regulation scores do not differ significantly from the normative validation samples. The scores to Patient Health Engagement Scale (PHE-S) on the adaptation to the disease show that the majority of the subjects lays on a level of “alert” or “adhesion” toward the disease.

**Conclusion:** First of all, our results seem to confirm the higher levels of stress found in pediatric C1-INH- HAE patients. Moreover, the adaptation to the disease measured by the PHE-S, that refers to the level of eudaimonic wellbeing, is low regardless to the years elapsed from the diagnosis. These results seem to suggest that the clinical features of C1-INH-HAE, such as the high unpredictability of the attacks may impair patients’ capacities to adapt to the disease and to cope with stress. An enlargement of the sample is needed to confirm and enrich such results. In a second phase, a broader aim of our project will be focused on developing an ad hoc counseling intervention focused on the disease management.

### P12 Depression in hereditary angiodema can cause sexual morbidity

#### Sevgi Yatarkalmaz^1^, Gökten Bulut^1^, Melih Özışık^1^, Özlem Kuman^2^, Okan Gülbahar^1^, Semiha Özgül^3^, Nihal Mete Gökmen^1,*^

##### ^1^Division of Allergy and Immunology, Department of Internal Medicine, Ege University Faculty of Medicine, Bornova, Izmir, Turkey; ^2^Department of Psychiatry, Ege University Faculty of Medicine, Bornova, Izmir, Turkey; ^3^Department of Biostatistics and Medical Informatics, Ege University Faculty of Medicine, Bornova, Izmir, Turkey

###### **Correspondence:** Nihal Mete Gökmen (enihalmete@yahoo.com.tr)

*Allergy, Asthma & Clinical Immunology* 2019, **15(Suppl 4):**P12

**Introduction:** Hereditary angioedema (HAE) is a rare disease characterized by recurrent attacks involving hand-arm-leg, face, bowels, genitals and upper airways. Although symptoms usually begin in the first 10 years of life, the frequency and severity of attacks increase in adolescence. In this study we analyzed that how the sexual life quality is affected by HAE attacks’ severity and frequency, attacks after sexual intercourse and anxiety and depression in HAE.

**Material and method:** Forty-eight patients who have been followed at Ege University Medical Faculty Internal Medicine HAE special clinic were enrolled in this study. Socio-demographic characteristics of the patients, disease onset age, attack localization and frequency, gender-specific sexual life questions were assessed. Hospital Anxiety and Depression Scale (HAD) and new sexual satisfaction scale (NSSS) were also applied.

The adaptation and reliability studies of HAD for the Turkish population showed that 7 were cut-off scores for depression and 10 were cut-off scores for anxiety. Higher values than these cut-off points were defined as depression risk and anxiety disorder risk.

**Results:** Of the 48 patients with the average age 39.89 ± 13.68; 26 (54.2%) were female. In females, the mean score of NSSS was 65 ± 14.24 and the mean anxiety score 8.38 ± 3.16 and the mean depression score was 6.1 ± 3.85; whereas mean scores of males were 72.95 ± 17.71, 7.4 ± 3.69 and 5.36 ± 3.27, respectively. The mean NSSS score of the patients which had attacks depending on sexual intercourse (n = 10; 63.8 ± 25.02) was lower than the patients which did not have attacks (n = 32; 71 ± 12.96) (t = 0.87, p = 0.402). The NSSS mean score of the patients with depression risk (n = 14) was significantly lower than those who had no risk of depression (n = 29); [61.57 ± 15.04 vs 72.69 ± 16.06; (t = 2.17; p = 0.036)]. When both sexes were examined separately for NSSS score; in only males, the risk of having depression affected the mean NSSS total scores [with depression risk n = 7; 60.42 ± 17.94) and without depression risk (n = 15; 78.8 ± 14.75) (t = 2.543; p = 0.019)].

**Discussion:** The sexual life of HAE patients with depression risk are affected negatively, therefore an intervention for this group should be planned. We believe that this study shed light on the related problem and will lead to further studies. We did not find any significant finding in women. Most probably female patients had not been able to answer the questions honestly due to the privacy of sexual satisfaction scale questions.

### P13 Immigrants’ perspective on living with hereditary angioedema in Denmark – a qualitative study

#### Malin Thomsen Sandberg^*^, Trine Svenssson, Anette Bygum

##### HAE Centre Denmark, Department of Dermatology and Allergy Centre, Odense University Hospital, Odense, Denmark

###### **Correspondence:** Malin Thomsen Sandberg (malin.sandberg@rsyd.dk)

*Allergy, Asthma & Clinical Immunology* 2019, **15(Suppl 4):**P13

**Background:** In the last decades several migrants with C1-inhibitor hereditary angioedema (C1-INH-HAE), a rare and potentially life-threatening disease, has moved to Denmark. HAE is known to impact quality of life (QoL) and patients often face a high disease burden, but there is a lack of data pertaining immigrants living with this rare disease. The objective of this study is therefore to investigate and improve our understanding of how patients of other ethnic backgrounds experience living with HAE in Denmark, and to gain deeper insight into these patients’ understanding of disease, challenges and management of symptoms in their everyday life. We also explore their past and familial experiences from their countries of origin.

**Materials and methods:** The study is designed as a qualitative study, based on semi-structured interviews, to ensure a deep and broad understanding of the patients’ experiences. The participants are recruited from the group of adult immigrants that are treated at the national HAE center. In addition to the interviews, patients are asked to complete two quality of life questionnaires, EQ-5D and HAE-QoL. The anonymized data will be analyzed descriptively.

**Results:** 9 out of 13 adult immigrants with HAE in Denmark have chosen to participate in the study (age 21–60 years). Collection of data is ongoing until April 2019 and results of selected thematic areas will be presented. Preliminary results show that one main concern for several migrant patients is not receiving accurate treatment at emergency departments, when they present with severe attacks and emergency medication.

**Conclusion:** This study is expected to provide unique information about migrants experiences living with a rare disease in Denmark. Preliminary data highlights the challenges, but also importance of teaching migrant patients home therapy, as is also the case for ethnic Danish patients.

### P14 Description of angioedema episodes prompting a call on the bradykin mediated angioedema reference centre on-call hotline

#### Nicolas Simon, Isabelle Boccon-Gibod^*^, Catherine Mansard, Alban Deroux, Alexis Bocquet, Laurence Bouillet

##### National Reference Center for Angioedema, Internal Medicine Department, Grenoble University Hospital, France

###### **Correspondence:** Isabelle Boccon-Gibod (iboccon-gibod@chu-grenoble.fr)

*Allergy, Asthma & Clinical Immunology* 2019, **15(Suppl 4):**P14

**Introduction:** Angioedema (AE) are acute, localized subcutaneous swellings that regress over hours to days. Their severity and treatment differ depending on the underlying cause: mast cell (MC) or bradykinin (BK) mediated angioedema. To this date, no reliable diagnostic tests are available to rapidly differentiate these forms of AE.

**Objective:** This study aimed to describe the possible underlying cause of an AE episode after a call on the French national reference center for AE hotline.

**Methods:** Physicians calling on the CREAK hotline between March and August 2018 were asked to fill a clinical description form for the AE episode. Patients were classified in the groups AE in the presence of a renin-angiotensin system inhibitor (ACE inhibitor or Angiotensin receptor blocker) (Drug-AE), mast cell mediated AE (MC-AE) and possibly bradykinin mediated AE in the absence of a renin-angiotensin inhibitor (BK-AE).

**Results:** 88 patients were included. 41 (48.8%) in the Drug-AE group, 39 (46.4%) in the MC-AE group, and 4 (4.8%) in the BK-AE group. The patients in the drug-AE group had more lingual and respiratory AE, and a tendency (p = 0.057) to more frequently require monitoring in intensive care units (ICU). 79.1% of calls originated from an emergency department(ED) or an ICU.

**Conclusion:** This study highlights the importance of AE episodes in EDs or ICUs concerning patients under renin-angiotensin system inhibitors. No calls were made for known bradykinin mediated AE patients, as these patients are educated to the treatment of acute episodes. The absence of validated diagnostic criteria or tests to formally differentiate mast cell mediated AE in patients treated by an ACEi from an authentic ACEi induced BK-AE render the use of costly and restricted treatments unavoidable in urgent settings like the ER or ICUs. These patients should be addressed to specialists to avoid ulterior inappropriate treatment in case of recurrence.

### P15 Quality of life among HAE patients in South West England (Devon and Cornwall) using HAE-QoL questionnaire designed by Foundation for Biomedical Research of La Paz University Hospital Madrid (FIBHULP)

#### Christine C. Symons^1,*^, Claire A. Bethune^1^, Andrew F. Whyte^1^, Lucy Leeman^1^, Teresa Caballero^2^

##### ^1^Department of Clinical Immunology and Allergy, University Hospitals Plymouth NHS Trust (UHPNT), Plymouth, United Kingdom; ^2^ Foundation for Biomedical Research, Hospital Universitario La Paz Madrid (FIBHULP), Madrid, Spain

###### **Correspondence:** Christine C. Symons (christine.symons1@nhs.net)

*Allergy, Asthma & Clinical Immunology* 2019, **15(Suppl 4):**P15

**Background:** Hereditary Angioedema (HAE) is a rare but potentially life-threatening inherited condition. HAE is characterised by episodes of angioedema affecting various body parts including the hands, feet, face and airway. Incidence is approximately 1 in 50,000. The Department of Clinical Immunology and Allergy at University Hospitals Plymouth has 43 patients with HAE from across Devon and Cornwall: an incidence of approximately 1 in 42,000. Dedicated HAE clinics are held 3 times a year where patients are seen, clinically assessed, have treatment reviews and are encouraged to enroll in clinical trials and research studies or have ongoing review by the research team.

**Method:** In June 2017 permission was granted to the lead author to use the HAE-QoL v.2 questionnaire, and the project was registered with UHPNT Audit Department.

Between October 2017 and February 2019 patients attending HAE clinics were approached to complete the questionnaire and 24 agreed. All HAE patients in our clinics have access to a range of therapies. Some prefer to take traditional oral prophylactic therapies (attenuated androgens, tranexamic acid); others treat symptoms with icatibant or C1 esterase inhibitor concentrate (C1INH). C1INH (plasma-derived or recombinant) is available for short-term prophylaxis (pre-surgery, dental) or longer term (school/college exams) according to the UK HAE consensus document [1].

**Results:** The questionnaire comprises 25 questions reflecting the previous 6 months. The answers are graded out of 5 or 6 with higher score reflecting ‘not a problem’ and lower score ‘extremely’. Answers are captured into 7 dimensions: physical functioning and health, disease related stigma, emotional role and social functioning, concern about offspring, perceived control over illness, mental health, and treatment difficulties. The results are presented here by gender and age ranges for each dimension. Note: no female patients in this cohort fell into the age range 35 – 50 years.

**Conclusion:** High scores across all dimensions (mean and median scores) suggest this group of patients has reasonably good quality of life apart from one outlier, a female aged 50 + . However lower scores for perceived control over illness highlight the unpredictable nature of HAE attacks even if few patients experience treatment difficulties. The author intends to repeat this study when new ‘pipeline’ prophylactic medications become available for UK patients.

**Acknowledgements:** Alex Symons, MSc Student Archaeological Science, University of Oxford for help with data presentation.
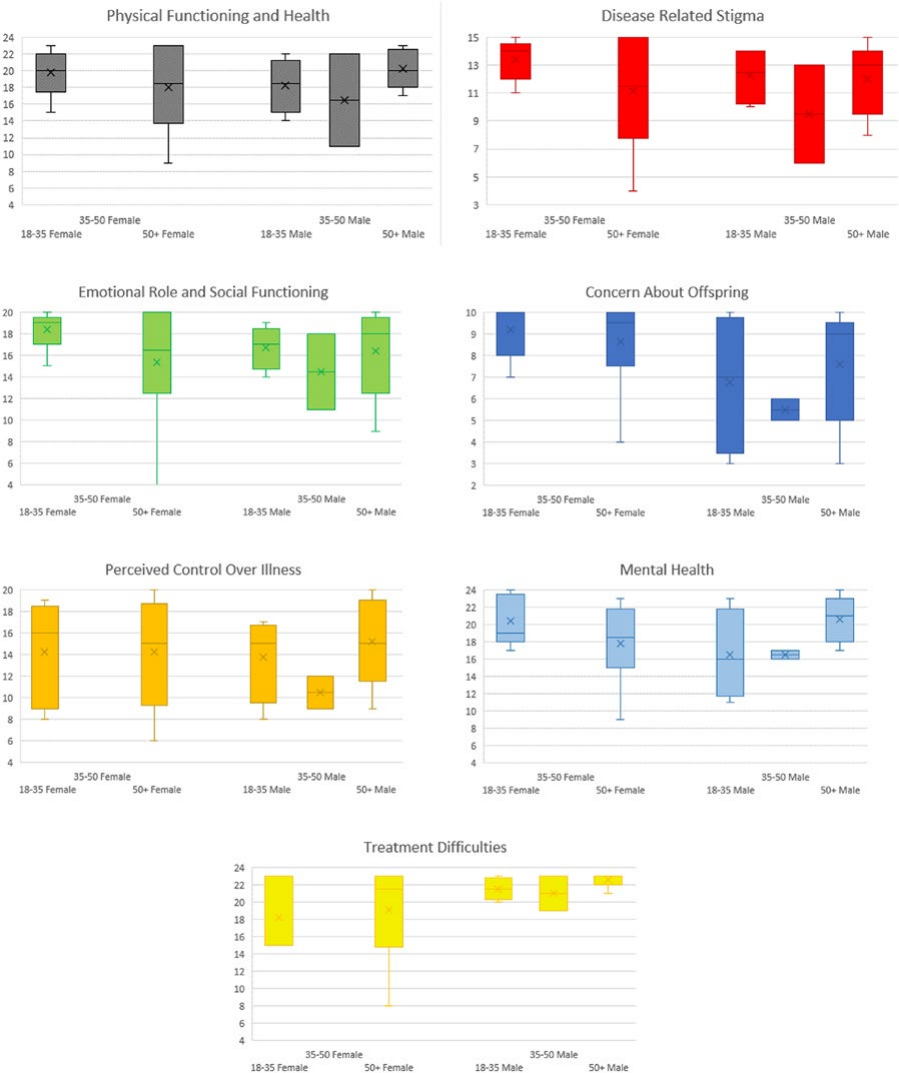


**Reference**

1. H.J. Longhurst et al. C1-inhibitor deficiency: 2014 United Kingdom consensus document. Clin & Exp Imm 2015 **180:** 475-483

### P16 First kinetic follow-up of coagulation and fibrinolytic parameters in a single edematous attack of a patient with hereditary angioedema

#### Nóra Veszeli^1,2,*^, Erika Kajdácsi^2^, Kinga Viktória Kőhalmi^2,3^, György Temesszentandrási^3^, Katalin Várnai^4^, Adrienne Fehér^4^, László Cervenak^2^, Lilian Varga^2,3^, Henriette Farkas^2,3^

##### MTA-SE Research Group of Immunology and Hematology, Hungarian Academy of Sciences and Semmelweis University, Budapest, Hungary^; 2^ Research Laboratory, 3^rd^ Department of Internal Medicine, Semmelweis University, Hungary; ^3^ Hungarian Angioedema Reference Center, 3^rd^ Department of Internal Medicine, Semmelweis University, Hungary; ^4^ Department of Laboratory Medicine, Semmelweis University, Budapest, Hungary

###### **Correspondence:** Nóra Veszeli (veszeli.nora88@gmail.com)

*Allergy, Asthma & Clinical Immunology* 2019, **15(Suppl 4):**P16

Hereditary angioedema with C1-inhibitor deficiency (C1-INH-HAE) is characterized by recurring and spontaneously resolving edematous attacks with not fully understood pathomechanism. Previously many studies published on the activation of plasma enzyme systems during edematous attacks, nevertheless kinetic follow-up has never been performed. For the first time, we aimed to study the kinetics of parameters in the coagulation and fibrinolytic systems in a spontaneously resolved edematous attack of a C1-INH-HAE patient.

In a 56-year-old female with C1-INH-HAE we monitored the severity of the symptoms during the observation period and altogether twelve blood samples were obtained. Blood samples collected from a healthy control volunteer at 5 different times during a 24–hour period. We measured Factors XI and XII activities (FXIa, FXIIa), prothrombin time (PT) and activated partial thromboplastin time (aPTT) as well as concentration of Factor V, VII and X (FV, FVII and FX respectively), prothrombin fragment 1 + 2 (F1 + 2), thrombin-antithrombin (TAT)-complex, D-dimer, fibrinogen.

After a 24-hour symptom-free period and another 19-hour prodromal period (characterized by erythema marginatum), the patient had a 29-hour-long edematous attack in multiple skin locations, and was followed up for another day. During prodromal stage—the levels of D-dimer, F1 + 2 and TAT-complex were as constantly low as those levels measured in the healthy control, whereas fibrinogen level increased. Levels of F1 + 2 and TAT-complex were significantly elevated at the onset of edematous symptoms whereas level of D-dimer was elevated after 6 h. Levels of all three parameters reached maximum 12 h after reaching the maximum severity score of symptoms. FXIIa and FXIa as well as the levels of FV, FVII and FX did not show unidirectional changes during the observation period. PT and aPTT did not shorten before or during the edema.

Real-time monitoring of F1 + 2 and TAT complex suggest that thrombin may contribute to edema formation. Whereas the generation of thrombin probably not related to the activation of neither the intrinsic nor the extrinsic pathway of coagulation but related to other factors such as MASP-1 or MASP-2. We confirmed that D-dimer is a prominent biomarker of an ongoing edematous attack while fibrinogen is could be a biomarker of prodromal period. This study was a part of a project aimed to better understandthe mechanisms leading to the onset and to the resolution of edematous attack.

This study was supported by OTKA 112110 and the ÚNKP-16-3 New National Excellence Program of the Ministry of Human Capacities”.

**Consent to publish:** Written, informed consent for publication was obtained from the patient and control person.

### P17 Development and verification of a quantitative systems pharmacology disease model of hereditary angioedema

#### Rangaraj Narayanan^1,*^, Dan Sexton^1^, Haobin Luo^2^, Zhiwei Zhang^2^, Paul Jasper^2^, Jon Kenniston^1^, Madhusudan Natarajan^1^, Patrick Martin^1^, Devin Welty^1^

##### Shire (now part of Takeda), Cambridge, MA, United States; ^2^ RES Group, Inc., Needham, MA, United States

###### **Correspondence:** Rangaraj Narayanan (rangaraj.narayanan@takeda.com)

*Allergy, Asthma & Clinical Immunology* 2019, **15(Suppl 4):**P17

**Background:** Hereditary Angioedema (HAE) is a long-term, debilitating, and potentially life-threatening disease caused by mutations in the C1-INH gene, resulting in deficiency (Type I) or dysfunction (Type II) of C1-INH protein. HAE manifests clinically as unpredictable, intermittent attacks of subcutaneous or submucosal edema of the face, larynx, gastrointestinal tract, limbs, and/or genitalia (Zuraw, 2008). The underlying mechanism is due to the excess activation of the contact system wherein plasma kallikrein (PKa), formed following factor XII activation, acts on high molecular weight kininogen (HMWK) leading to release of bradykinin, a potent vasodilator. Bradykinin binds to B2 receptors on endothelial cells resulting in vascular leak and edema. A quantitative understanding of the disease process was undertaken using QSP modeling.

**Materials and methods:** A mechanistic biological model of HAE disease was developed using QSP modeling that incorporated critical components of the contact system – compiled from the scientific literature and a population of virtual HAE patients was established. Cleaved HMWK pharmacodynamic biomarker data and outcome results from clinical studies with lanadelumab, a recently approved monoclonal antibody inhibitor of PKa for prophylaxis to prevent HAE attacks, were used to verify the HAE disease model.

**Results:** The HAE disease model successfully described the decreased cleaved HMWK observed in patients following administration of all lanadelumab doses evaluated in the Phase 3 study, i.e. 300 mg Q2 W, 300 mg Q4 W and 150 mg Q4 W. The corresponding impact on disease outcome – reduction of HAE attacks per month – was also successfully recovered and supported model verification. The utility of the model to potentially improve understanding of HAE pathophysiology was demonstrated through the estimation of kinetics of cleaved HMWK inhibition and bradykinin levels generated between and during an attack

**Conclusions:** A mechanistic biological model of HAE disease was successfully established using QSP modeling and verified using lanadelumab clinical data.

### P18 Serum complexes between C1-INH and C1-INH-autoantibodies for the diagnosis of Acquired Angioedema

#### Alberto López-Lera^1,2,*^, Sofía Garrido^2,3^, Pilar Nozal^2,3^, Lillemor Skatum^4^, Anette Bygum^5^, Teresa Caballero^1,2,6^, Margarita López Trascasa^1^

##### Instituto de Investigación Sanitaria del Hospital La Paz (IdiPaz), Madrid, Spain; ^2^ Centre for Biomedical Network Research on Rare Diseases (CIBERER) U-754, Madrid, Spain; ^3^ Immunology Unit, Hospital Universitario La Paz, Madrid, Spain; ^4^ Clinical Immunology and Transfusion Medicine, Office for Medical Services, Lund, Sweden; ^5^ National HAE Centre, Odense University Hospital, Odense, Denmark; ^6^ Department of Allergy, Hospital Universitario La Paz, Madrid, Spain

###### **Correspondence:** Alberto López-Lera (alberlole@gmail.com)

*Allergy, Asthma & Clinical Immunology* 2019, **15(Suppl 4):**P18

**Background**: Acquired angioedema due to C1-inhibitor (C1INH) deficiency (AAE) is an ultra rare disease caused by secondary C1INH deficiency leading to bradykinin-mediated angioedema. AAE typically presents in adulthood and is significantly associated to B cell lymphoproliferation. Anti-C1INH autoantibodies (antiC1INHAbs) are detectable in a subset of AAE cases and considered a hallmark of the disease. When free antiC1INHAbs and malignant tumours are not detectable, diagnosis of AAE relies on the finding of low C1INH levels and/or function, lack of family history, no C1NH gene mutation, age at onset of angioedema symptoms and low or undetectable C1q levels.

**Objectives**: To test the diagnostic value of a novel ELISA assay for the detection of circulating complexes between C1INH and C1INH-autoantibodies (C1INH-antiC1INH) in serum.

**Methods**: An in-house sandwich ELISA was designed to measure free antiC1INHAbs and complexed C1INH-antiC1INHAbs. Twenty European patients with AAE were included and characterized on the basis of their complement levels and function.

**Results:** Free antiC1INHAbs were detected in 9/20 patients (6 of IgG class, 2 of IgM class and one simultaneously presenting IgG and IgM classes), whereas C1INH-antiC1INH complexes were found in 90% (18/20) of the AAE cases, regardless of the presence or absence of detectable free anti-C1INHAbs. It is of note that 9/20 (45%) patients showed negative serum free antiC1INHabs, but positive C1INH-antiC1INH complexes in their first measurement. In the cohort presented, IgM-class C1INH-Ab’s are specifically and strongly associated to undetectable C1q serum levels.

**Conclusion**: Detection of C1INH-antiC1-INHAbs provides a diagnostic added value for AAE diagnosis, especially in those cases in whom no free anti-C1INH antibodies are detected. The link between IgM-class C1INH-antiC1INH complexes and C1q consumption could have further implications for the development of autoimmune manifestations in AAE.

### P19 The Romanian Hereditary Angioedema Center started the Global Hereditary Angioedema Registry activities

#### Noémi-Anna Bara^1,*^, Enikő Mihály^1^, Valentin Nădășan^1^, Attila Borka-Balás^1^, Ruggero di Maulo^2^, Alice Pizzolato^2^, Carlotta Cicardi^2^, Marco Cicardi^3^, Dumitru Moldovan^1^

##### Hereditary Angioedema Expertise Center, Mediquest Clinical Center, Sîngeorgiu de Mureș, Romania; ^2^ Cloud-R Srl, Technology Startup of Polihub Innovation District of Milan, Italy; ^3^ Universita Degli Studi Di Milano, Milan, Italy

###### **Correspondence:** Noémi-Anna Bara (noemi.bara@yahoo.com)

*Allergy, Asthma & Clinical Immunology* 2019, **15(Suppl 4):**P19

**Introduction:** Taking into consideration that hereditary angioedema is a rare disorder, studies on a large number of patients are still wanted. The Global Hereditary Angioedema Registry is a common, worldwide platform that collects demographic and clinical data from consenting people with hereditary angioedema, in accordance with agreed inclusion criteria and definitions.

**Methods:** The Global Angioedema Registry was developed in 2017, by the Italian hereditary angioedema working group. Romania joined this registry in December 2018, the fifth country after France, Greece, and Hungary. The minimum requirements for affiliation included recruiting at least ten patients with a follow-up visit/phone contact with the center during the last two years and obtaining the signed informed consent from the patients. For each patient the following data were recorded: demographic characteristics (date of birth, gender, date of diagnosis, the age at diagnosis), plasma levels of C4 and C1-INH (antigenic and function), presence of family history of angioedema, concomitant diseases, frequency, location and severity of attacks, use of on-demand and prophylactic treatments. The data source was the medical record of individual patients. The acquisition of data was performed by the treating physician.

**Results:** Till January 2019, a total of 14 patients were included by the Romanian Hereditary Angioedema Center: nine female and five men with C1-INH-HAE (10 type1 and four type 2), median age 45, median age at diagnosis 26. Eight patients had positive family history for angioedema. Based on patient diary, between April-November 2018, 144 attacks were recorded: 85 peripheral, 86abdominal and 13 laryngeal. Sixty-six of the attacks were severe, 71 moderate and seven mild. One hundred and twelve attacks were treated with subcutaneous injection of Icatibant and four with additional plasma-derived C1-INH. Ten patients were on prophylactic therapy.

**Conclusions:** A prospective long-term registry is a critical tool in building a broad and comprehensive knowledge base. By continuing to introduce valid data from as many patients as possible,useful information can be obtained for the better knowledge of thisrare disease.

### P20 Hereditary angioedema: quality of life in 19 patients

#### Ana Lainez Nuez^*^, Irene García Gutiérrez, Alberto Álvarez-Perea, Alicia Prieto García, Maria Luisa Baeza Ochoa de Ocariz

##### Allergy Service, Hospital Universitario Gregorio Marañón, Madrid, Spain

###### **Correspondence:** Ana Lainez Nuez (alainuez@gmail.com)

*Allergy, Asthma & Clinical Immunology* 2019, **15(Suppl 4):**P20

**Background:** Hereditary angioedema (HAE), is characterized by recurrent episodes of angioedema that expose patients to the risk of asphyxiation and have considerable impact on their quality of life (QoL). New therapies have been developed for treating or preventing attacks. On demand treatment is the first option, however many of them need long-term prophylaxis (LTP). The aim of this study is to evaluate the QoL of patients with HAE according to their treatment schedules.

**Materials and methods:** 24 patients with HAE attended at the Gregorio Marañón University hospital were asked to complete the AE-QoL questionnaire. Nineteen returned the fulfilled document. AE-QoL questions are grouped into four domains (Functioning, Fatigue/Mood, Fears/Shame and nutrition). Individual domain scores were transformed into a linear 0–100% scale where higher scores are indicative of a higher QoL impairment. Patients were classified in Group A: patients on “on demand” treatment; Group B: received LTP (plasma-derived C1INH 63%, attenuated androgens 28% or tranexamic acid 9%); Group B1: i.v. treatment; Group B2: LTP with oral drugs.

**Results:** Group A, n = 8, (50%F), average age of 53y (range: 26–86). Group B, n = 11, (64%F), average age of 53y (range: 28–73). 62.5% patients from group A have type 1 HAE-C1INH, 25% type 2 and 12.5% nC1-INH (HAE-FXII). 100% group B have type 1 HAE-C1INH. The average age at the time of the first symptoms was 15.75y in group A and 4.9y in group B, (p = 0.17). The average time between onset of symptoms and diagnosis was 12.12y in group A and 9.82y in group B (p = 0.9). AE-QoL scores from group A and group B were: Functioning domain: 16% *vs* 19.31%; Fatigue/Mood dimension: 23.33% *vs* 36.36%; Fears/Shame domain: 18.97% *vs* 24.98%; Nutrition domain: 22.22% *vs* 25%. No score exceeded > 40%. No significant differences in QoL were found between both groups. A comparison between B1 and B2 and B1 and A groups was performed. Significant increased differences were only found in the functioning domain between group B1 and B2 17.85% *vs* 21.87% (p < 0.05).

**Conclusion:** To achieve an optimal control of symptoms, a precise treatment adapted to the severity of the disease is needed. According to this questionnaire, patients had an impaired QoL most prominent in the fatigue/Mood dimension. Surprisingly, no significant differences in QoL were found between patients requiring LTP, more severe cases, and patients in “on demand” treatment. The long interval between the onset of symptoms and the confirmation of the diagnosis was notorious in both groups.

### P21 The French side of the Global Angioedema Registry

#### Isabelle Boccon-Gibod^1,*^, Maddalena A. Wu^2^, Ruggero Di Maulo^3^, Marta De Munari^3^, Luca De Roberto^3^, Anne Pagnier^1^, Catherine Mansard^1,^ Alban Deroux^1,^ Mélanie Arnaud^1^, Nelly Carrat^1^, Marco Cicardi^2^, Laurence Bouillet^1^

##### ^1^Department of Internal Medicine, Centre Hospitalier Universitaire de Grenoble Alpes, National Reference Center for Angioedema, Grenoble, France; ^2^Department of Biomedical and Clinical Sciences “Luigi Sacco”, University of Milan, Luigi Sacco Hospital, Milan, Italy; ^3^CEO Cloud-R s.r.l., Milan, Italy

###### **Correspondence:** Isabelle Boccon-Gibod (iboccon-gibod@chu-grenoble.fr)

*Allergy, Asthma & Clinical Immunology* 2019, **15(Suppl 4):**P21

**Rationale:** Angioedema is a recurrent localized swelling of cutaneous and mucosal tissues. Potentially life-threatening, creates temporary disability which deteriorates quality of life. Seven inherited or acquired forms of angioedema without wheals are yet classified, included hereditary or acquired C1-inhibitor deficiency (C1-INH-HAE and C1-INH-AAE). This year, the French angioedema network (CREAK) joined the registry of angioedema without wheals **(Cloud-R HAE).** Here we present the contribution of the Grenoble Alpes University Hospital (CHUGA) to this disease registry.

**Methods:** Study population is composed of C1-INH-HAE/AAE patients with a proved diagnosis.

The following items are collected: patients’ personal-demographic data, clinical/labora-tory/genetic characteristics, major comorbidities, treatments (prophylaxis/acute attacks).

As from Cloud-R HAE structure, patients can directly provide information on angioedema attacks and their treatment through a dedicated electronic app, web connection or paper support, which is then transferred into the registry at CHUGA.

The Study Protocol has received approval from the Ethical Committee

**Results:** Since February 2018, 23 C1-INH-HAE patients have been included (informed consent signed). Within C1-INH-HAE, median age is 44 years (range 12–72), sex-ratio: 6/17 (M/F). Seventeen per cent of them provide prospective data on angioedema attacks. 71% of attacks have been treated. Due to the frequency of symptoms, 52% of them are on long-term prophylaxis (LTP) with tranexamic acid (8%), Danazol (8%), C1-INH concentrate or recombinant (16%) and 34% with progestin. Since February 2019, 8 additional centers have joined the registry: they included 13 more patients without event yet.

**Conclusions:** Angioedema registry gives the possibility to gather information to define natural history of angioedema and to evaluate treatment efficacy in real life. The possibility that data from single countries merge into a global structure facilitates improvement and dissemination of the knowledge on this rare disease and its treatment

### P22 Designing and delivering Educational Therapeutic Program training kit for HAE patients across France

#### Isabelle Boccon-Gibod^1,*^, Mélanie Javaud^2^, Isabelle Citerne^3^, Patricia Minasi^4^, Hélène Humeau^5^, Astride Khune^6^, Kevorkian Charlotte^7^, Anne Pagnier^7^, Laurence Bouillet^1^, Johanna Limone^1^, Nelly Carrat^1^

##### ^1^Department of Internal Medicine, Grenoble Alpes University Hospital, National Reference Center for Angioedema, Grenoble, France^; 2^Department of Internal Medicine, Saint Antoine University Hospital, National Reference Center for Angioedema, Paris, France; ^3^Department of Internal Medicine, Lille University Hospital, National Reference Center for Angioedema, Lille, France; ^4^Department of Internal Medicine, Nice University Hospital, National Reference Center for Angioedema, Nice, France; ^5^Department of Internal Medicine, Angers University Hospital, Angers, France; ^6^Department of Internal Medicine, Strasbourg University Hospital, Strasbourg, France; ^7^Pediatric Department, Grenoble Alpes University Hospital, National Reference Center for Angioedema, Grenoble, France

###### **Correspondence:** Isabelle Boccon-Gibod (iboccon-gibod@chu-grenoble.fr)

*Allergy, Asthma & Clinical Immunology* 2019, **15(Suppl 4):**P22

**Rationale:** In 2016, the French Educational Program “EDUCREAK” was assessed by the French Health Care Agencies (ARS) and further approved for an additional 4 years. This pilot program was initially developed in cooperation with staff from four sites in France. After the program’s implementation and success in four years, our new objectives are to scale up the program on a national level and streamline best practices among HAE healthcare professionals (HCP).

**Methods:** To reach these objectives, meetings and workshops were held to interactively share the different experiences and methods used by HCP. From these sessions we reflected and drew conclusions on the optimal way to cater to both adult and children HAE patients. We weighed costs and grants required to deliver adequate outputs. In a participatory approach, patients and HCP were asked to work together to attain common objectives, guided by a step-by-step explanatory sheet. Training kit components were then tested by HAE advocate patients and HCP in the last five months to ensure satisfaction. An evaluation system has been established to keep measuring the kit’s efficacy. Indicators are not only based on autonomous and safe patient skills but also on quality of life (HAE-QoL and AE-QoL scores)

**Results:** According to the above criteria, an educational training kit was designed and delivered to assist HCP in providing skills for quality care for HAE patients. It has now been streamlined in 12 sites across the country (an additional 8 compared to the previous 4). We thank the French Ministry of Health for their financial support to the National Reference Centre for Angioedema “CREAK”.

**Conclusions:** We are pleased to present a valuable training kit for HAE patients and their HCP. In May, the training kit will be launched in Paris. Next steps include ensuring the training of HCP on using the training kit and systematically evaluating the 10 different sites where they will be used.

### P23 Daily routine laboratory assessment of C1-INH function – different methods, different results, different interpretations

#### Peter J. Späth^1,*^, Brunello Wüthirch^2^

##### ^1^Institute of Pharmacology, University of Bern, Bern, Switzerland; ^2^Allergy Unit, University of Zurich, Zurich, Switzerland

###### **Correspondence:** Peter J. Späth (peter.spaeth@pki.unibe.ch)

*Allergy, Asthma & Clinical Immunology* 2019, **15(Suppl 4):**P23

**Background:** We have compared performance of three marketed C1-INH functional assays (C1-INH[f]) two decades ago. We have presented only a small part of results scattered over various publications. We present complete data set here because (i) test kits used at that time being in use in routine laboratory diagnostics nowadays; (ii) not being aware of similar extensive comparative studies and; (iii) the COMPACT study having used one of these tests. This is a supplementary to an other abstract we present in this journal.

**Methods:** C1-INH[c] routine diagnostic assessment vs at least one of the C1-INH[f] assays was in 2038 samples from patients suffering from autoimmune/inflammatory diseases, supposed complement deficiencies, their family members and some clinical studies. Validated assays were performed for C1-INH[c] by radial immunodiffusion and for C1-INH[f] using one with read-out by complex formation of C1-INH with its target protease C1s (A, Quidel) and two with read-outs using chromogenic substrates CH_3_CO-R (B1, Berichrom) or C_2_H_5_CO-R (B2, Technochrom; R = Lys(ε-Cbo)-Gly-Arg-pNA).

**Results:** The three tests prove useful and perform well for the majority of C1-INH dysfunction-associated HAEs. Over the entire concentration range measured, a linear relationship (r = 0.80 and r = 0.81, respectively) exist for C1-INH[c] vs [f] for B1 and B2, i.e. a gradual increase in C1-INH[c] showed comparable gradual increase in C1-INH[f_B1 or B2_], while increase in C1-INH[f_A_] was prominent at low and flattened at higher concentrations. In a range of C1-INH[c] of 35–45% (100% = 0.196 g/L), a concentration range at which frequency of HAE attacks starts weaning, 5.5% of [f_B1_], 1.3% of [f_B2_] while 24.2% of [f_A_] were within the normal range.

**Conclusion:** C1-INH[f] by B1 and B2 between 50 and 70%, i.e. below the normal range (NR), is not convincingly associated with C1-INH-HAE, while for A values even within the NR can be indicative for C1-INH dysfunction. B1 and B2 appear less sensitive to patients’ condition at time point of sample drawing and more suitable for initial laboratory diagnosis. Patients’ condition at time point of venepuncture and response to treatment however seem better mirroring by A. Interpretation of values obtained by method A needs much more expert knowledge. To our opinion, assessment of concentration and function together is best for laboratory diagnosis of C1-INH dysfunction associated AE.

### P24 Genetic segregation study in angioedema with normal C1-inhibitor (n-C1-INH-HAE) in Southern Spanish population

#### Krasimira Baynova^1^, Teresa De Aramburu Mera^1,*^, Raul García Lozano^2^, Lourdes Lagarda^2^, Macarena Piñero Saavedra^1^, Teresa Gónzalez-Quevedo^1^

##### Andalusian Reference Unit of Angioedema, University Hospital Virgen del Rocío, Seville, Spain; ^2^ Inmunology Departament, University Hospital Virgen del Rocío, Seville, Spain

###### **Correspondence:** Teresa De Aramburu Mera (teresadearamburu@hotmail.com)

*Allergy, Asthma & Clinical Immunology* 2019, **15(Suppl 4):**P24

**Background:** In the last two decades have been more frequently described families with HAE with normal C1-INH levels and function. The pathophysiologic mechanism and the genetic basis of this type of HAE were unknown, but subject of recent studies. The field of medical genomics is rapidly growing and interpret genetic and genomic data is driving a new era of healthcare. It constitutes, therefore, a key component of personalized medicine.

Our objective was to study the possible genetic mutations in 8 unrelated families with nC1-INH-HAE.

**Materials and methods:** We have included 24 patients from 8 unrelated families with nl-C1-INH-HAE. In all of them, the index case had been previously analyzed and carried low prevalence variants of a panel of 55 selected genes encoding proteins related with Kallikrein-Kinin system. Some of the studied relatives presented angioedema and some of them were asymptomatic. For the familial study, we studied 45 genes out of theses 55 previously selected.. The genetic analysis was carried out through a customized NGS platform (Ampliseq, Thermo Scientific), examining the coding regions and the exon–intron splice junctions of these genes. Subsequently, the results were compared with the Ion Reporter v.5.2 software (Thermo Scientific).

**Results:** Of the 45 genes studied, we have found mutations in 22 out of 45 genes: SERPINA1, XPNPEP2, A2 M, KLK3, ESRRA, BDKRB2, PLG, PLAT, PLAUR, MME, TNF, F12, ACE, KLKB1, *SERPING1*, MPO, TLR4, ELANE, HSP90AA1, CPN1, SERPINE1, F13B. A big number of the detected mutations are not described previously. Most mutations occurred in heterozygosis and we can not know at present their clinical significance. We have found a mutation in homozygosis for PLAUR, present in one patient. The clinical situation as the age of the studied members varies between the different families, as well as between members of the same family, although most episodes of angioedema involve facial, peripheral and abdominal areas.

**Conclusion:** In the genetic segregation analysis we describe a series of heterogeneous mutations in our patients of n-C1-INH-HAE and in some of their asymptomatic relatives. Further studies are needed to found correlation among the detected mutations and clinical expression.

### P25 A deep intronic *SERPING1* variant associated with hereditary angioedema due to C1-inhibitor deficiency (C1-INH-HAE)

#### Sofia Vatsiou^1,2^, Maria Zamanakou^1^, Fotis Psarros^3^, Matthaios Speletas^2^, Gedeon Loules^1^, Faidra Parsopoulou^1,2^, Anna Valerieva^4^, Henriette Farkas^5^, Anastasios E. Germenis^1,2,*^

##### ^1^CeMIA SA, Larissa, Greece; ^2^Department of Immunology & Histocompatibility, School of Health Sciences, Faculty of Medicine, University of Thessaly, Larissa, Greece; ^3^Department of Allergology, Navy Hospital, Athens, Greece; ^4^Department of Allergology, Medical University of Sofia, Sofia, Bulgaria; ^5^ Hungarian Angioedema Center, 3^rd^ Department of Internal Medicine, Semmelweis University, Budapest, Hungary

###### **Correspondence:** Sofia Vatsiou (agermen@med.uth.gr)

*Allergy, Asthma & Clinical Immunology* 2019, **15(Suppl 4):**P25

In about 5% of C1-INH-HAE cases no mutation in the coding region of *SERPING1* can be detected.

Bearing in mind that non-coding variants can potentially have deleterious effects on a transcript through the regulation of splicing or transcription, defects located in an intronic or an untranslated region of the gene have been considered responsible for modifying C1-INH expression in these cases. However, until today no such alteration has been described. Here, we present a deep intronic *SERPING1* variant associated with type I C1-INH-HAE.

Six members of a Greek family with type I C1-INH-HAE were examined. Conventional genotyping of the family members by sequencing all *SERPING1* translated regions and intron–exon boundaries, long-range PCR and multiplex ligation dependent probe amplification (MLPA) did not reveal any defect. Analysis by a next-generation sequencing (NGS) platform targeting the entire *SERPING1* gene [1] uncovered a novel mutation (c.-22-155G>T) in intron 1 that was confirmed by Sanger sequencing. All four affected family members (3 men, 1 woman, mean age at disease onset 6 years) were carriers of the mutation while none of the two healthy members did. The same mutation was also detected in an additional unrelated Greek male patient and was not detected in 6 additional C1-INH-HAE families without defects in translated regions.

Bioinformatics analysis by the use of Neural Network (score 0.96, range 0–1), NetGene 2 Server (confidence 0.79, range 0.5–0.95), Alternative Splice Site Predictor (ASSP) (confidence 0.458, range 0–1) and FSPLICE (threshold 10.16) bioinformatics tools corroborated that c.-22-155G>T mutation creates an alternative donor site and as a result alters the splicing process. Analysis of the effect of the mutation on the SpliceAid 2 bioinformatics tool showed that it eliminates the number of possible proteins acting in the specific region. TraP score evaluating a single nucleotide variant’s ability to cause disease by damaging the final transcript, classified the mutation at the intermediate pathogenic range, akin to possibly damaging classifications (score 0.686, range 0–1). TraP evaluates the mutation based on DANN (score 0.9507, range 0–1) and GERP (conservation score 4.8, range − 12.3 to 6.17) prediction tools.

Up to now, 49 different intronic mutations in *SERPING1* gene have been associated with hereditary angioedema but all are located in the donor and acceptor site or a few nucleotides from these regions. Provided that it will be confirmed by appropriate functional tests, the c.-22-155G>T is the first deep intronic mutation associated with C1-INH-HAE.

**Reference**

1. Loules G, Zamanakou M, Parsopoulou F, et al. Targeted next-generation sequencing for the molecular diagnosis of hereditary angioedema due to C1-inhibitor deficiency. Gene. 2018; 667:76-82.

### P26 The silo effect in the annotation of *SERPING1* variation

#### Sofia Vatsiou^1,2^, Maria Zamanakou^1^, Faidra Parsopoulou^1,2^, Gedeon Loules^1^, Anastasios E. Germenis^1,2,*^

##### ^1^CeMIA SA, Larissa, Greece; ^2^Department of Immunology & Histocompatibility, School of Health Sciences, Faculty of Medicine, University of Thessaly, Larissa, Greece

###### **Correspondence:** Anastasios E. Germenis (agermen@med.uth.gr)

*Allergy, Asthma & Clinical Immunology* 2019, **15(Suppl 4):**P26

Defining the clinical validity and the pathogenicity of *SERPING1* variants is a particularly challenging task. Reporting of variants by reputable variant databases is considered as a criterion for their classification (criteria PP5 and BP6 of ACMG-AMP guidelines) [1].

We investigated the usefulness of public databases in regard to the interpretation of *SERPING1* variants. The ClinVar, an NCBI-funded primary centralized database for archiving clinically relevant variants for many diseases, and the HAEdb, a C1-inhibitor gene mutation database were examined.

Using an appropriate broad PubMed search query we retrieved 213 publications referred to 574 distinct *SERPING1* variants the great majority of which (95.3%) are associated with hereditary angioedema due to C1-inhibitor deficiency (C1-INH-HAE). Among these, 42.9% were missense/nonsense, 9.1% splice site and 0.6% regulatory variants, 35.5% were indels, and 11.9% large defects. At the same time, 544 out of the 567 *SERPING1* variants listed in the obtainable by subscription professional version of Human Gene Mutation Database (HGMD) [2] are reported to be disease-causing and five as likely disease-causing. By December 2018, 82 and 302 *SERPING1* variants had been reported in ClinVar and HAEdb, respectively. All variants reported in HAEdb but only 51% of those in ClinVar were reported in the literature (Fig. 1). In order to examine whether classifiable *SERPING1* variants reported in other databases were included in ClinVar and HAEdb, we filtered the 351 *SERPING1* variants reported in the Exome Aggregation Consortium (ExAC) database v.0.3.1 in regard to their global frequency, according to the BS1 criterion of ACMG-AMP guidelines. Variants with an allele frequency lower than the prevalence of angioedema (< 0.002%), and non-canonical transcript variants were excluded. ACMG-AMP criteria were applied to the remaining 99 variants using the available open-access evidence. 15/99 variants were found reported in ClinVar (2 benign, 8 likely benign, 2 variants of unknown significance -VUS- and 2 with multiple assertions) while only 4/99 in HAEdb (1 pathogenic, 1 unknown and 2 polymorphisms).

**Fig. 1:**
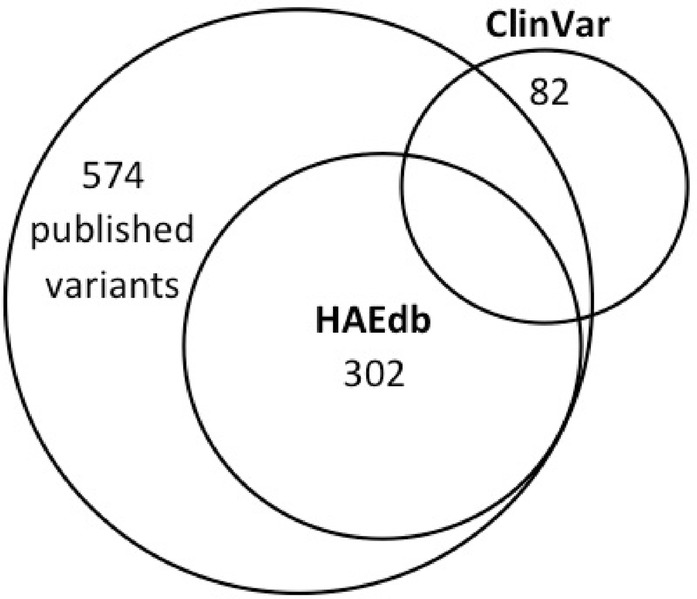
SERPING1 variants reported in the literature and the ClinVar and HAEdb databases

Public databases include less than 50% of classifiable *SERPING1* variants with ClinVar containing approximately 4-times less than HAEdb references. However, the ClinVar database allows for the deposition of variants with clinical observations and assertions, with review status tracked to enable a more transparent view of the levels of quality of the curation [3]. Therefore, clinical laboratories and researchers involved in genotyping angioedema patients are encouraged for real-time submission in ClinVar of variant data, including clinical assertions and evidence used for the variant classification.

**References**

1. Richards S, Aziz N, Bale S, et al. Standards and guidelines for the interpretation of sequence variants: a joint consensus recommendation of the American College of Medical Genetics and Genomics and the Association for Molecular Pathology. Genet Med. 2015; 17:405-424.
2. Stenson PD, Mort M, Ball EV, Shaw K, Phillips AD, Cooper DN. The Human Gene Mutation Database: building a comprehensive mutation repository for clinical and molecular genetics, diagnostic testing and personalized genomic medicine. Hum Genet 2014; 133:1–9.
3. Landrum MJ, Lee JM, Benson M, et al. ClinVar: public archive of interpretations of clinically relevant variants. Nucleic Acids Research. 2015; 44:D862–D868.

### P27 Variant pathogenicity curation in primary angioedema with normal C1-inhibitor (nl-C1-INH-HAE)

#### Gedeon Loules^1^, Faidra Parsopoulou^1,2^, Maria Zamanakou^1^, Dorottya Csuka^3^, Maria Bova^4^, Teresa González-Quevedo^5^, Fotis Psarros^6^, Gregor Porebski^7^, Matthaios Speletas^2^, Davide Firinu^8^, Tiziana De Pasquale^9^, Alessandra Zoli^10^, Anna Radice^11^, Stefano Pizzimenti^12^, Chiara Suffritti^13^, Emmanouil Manoussakis^14^, Marco Cicardi^13^, Henriette Farkas^3^, Anastasios E. Germenis^1,2,*^

##### CeMIA SA, Larissa, Greece; ^2^ Department of Immunology & Histocompatibility, School of Health Sciences, Faculty of Medicine, University of Thessaly, Larissa, Greece; ^3^ Hungarian Angioedema Center, 3rd Department of Internal Medicine, Semmelweis University, Budapest, Hungary; ^4^ Department of Translational Medicine, University of Naples, Italy; ^5^Reference Unit for Angioedema in Andalusia, Allergy Department, Virgen del Rocío University Hospital, Seville, Spain; ^6^ Department of Allergology, Navy Hospital, Athens, Greece; ^7^Department of Clinical and Environmental Allergology, Jagiellonian University Medical College, Krakow, Poland; ^8^ Department of Medical Sciences and Public Health, University of Cagliari, Italy; ^9^ Department of Allergy, Hospital of Civitanova Marche, Italy; ^10^Department of Clinical Immunology, Ospedali Riuniti, Ancona, Italy; ^11^Unit of Immunoallergology, Azienda Ospealiera Universitarta Careggi, Florence, Italy^; 12^Department of Allergy, University of Torino, Italy; ^13^Department of Biomedical and Clinical Sciences Luigi Sacco, University of Milan, Milan, Italy; ^14^Allergy Unit, 2^nd^ Pediatric Clinic, University of Athens,“P & A Kyriakou” Children’s Hospital, Athens, Greece

###### **Correspondence:** Maria Zamanakou (agermen@med.uth.gr)

*Allergy, Asthma & Clinical Immunology* 2019, **15(Suppl 4):**P27

**Background:** Last year, genomic analysis allowed the discovery of two variants pathogenic for nl-C1-INH-HAE in two different genes (*PLG* and *ANGPT1*). However, the implementation of high throughput DNA sequencing results in a dramatic increase of variants identification the classification of which represents a challenging task. Here, we present the variant pathogenicity curation following the next-generation sequencing (NGS) genotyping of nl-C1-INH-HAE patients.

**Materials and methods:** 130 unrelated patients with nl-C1-INH-HAE (53 Hungarian, 32 Italian, 27 Spanish, 9 Polish, 9 Greek) were submitted to targeted genotyping focused on 55 genes possibly involved in angioedema pathogenesis. The gene list was compiled from literature data on angioedema and genetic predisposition, protein–protein interaction networks, and pathway analysis.

169 patients with HAE due to C1-inhibitor deficiency (C1-INH-HAE) were genotyped as controls. A NGS custom platform (Ampliseq, Thermo Scientific) was developed by which all coding regions and exon–intron splice junctions of these genes (coverage > 90%) were analyzed. Analysis of primary data was conducted with Ion Reporter software v.5.2 (Thermo Scientific). Variants with worldwide frequency > 1% (1000 Genomes Global Minor Allele Frequency, ExAC) and polymorphisms (UCSC Common SNPs) for which no disease associations are reported in the ClinVar database were excluded. SIFT and PolyPhen2 tools were used for bioinformatics prediction of the pathogenicity of the remaining uncommon variants. Family segregation studies were performed where feasible.

**Results:** Among nl-C1-INH-HAE patients, no carriers of the *ANGPT1* p.Ala119Ser variant were found while the *PLG* p.Lys330Glu was detected in 4 (2.9%) unrelated probands (one homozygote) along with other uncommon *PLG* variants (p.Arg89Lys, p.Arg253His, p.Arg490Gln, p.Arg523Trp). 259 uncommon variants were filtered in amongst which 35 mutations were not previously reported in population databases. Notably, the novel p.Tyr94Ser in *KRT1* was detected twice. 7/130 and 3/130 nl-C1-INH-HAE patients, but none of C1-INH-HAE patients, were found to be heterozygous of *KNG1* and *XPNPEP1* uncommon variants, respectively. Amongst them, the novel *KNG1* p.Pro574Ala variant was found segregated with the disease in an Italian family, while segregated compound heterozygosities were also detected in two other families.

**Conclusions:** We provide evidence indicating the existence of a heterogeneous genetic background linked with nl-C1-INH-HAE cases. Family segregation and functional studies are in progress for the pathogenicity or the disease modifying effect of these genetic alterations to be confirmed.

### P28 Hereditary angioedema: a report from the Republic of Belarus

#### Irina E. Guryanova^1,*^, Katsiaryna A. Paliakova^1^, Valeria V. Pugacheva^1^, Olga I. Puhach^2^, Victar I. Lebedz^2^, Mikhail V. Belevtsev^1^

##### ^1^Belarussian Research Center for Pediatric Oncology, Hematology and Immunology, Minsk, Belarus; ^2^Belarusian National Public Organization “HAE Patients Care”, Belarus

###### **Correspondence:** Irina E. Guryanova (guryanovairina1985@gmail.com)

*Allergy, Asthma & Clinical Immunology* 2019, **15(Suppl 4):**P28

**Background:** Hereditary angioedema due to C1-inhibitor deficiency (Type I) or dysfunction (Type II) is a rare genetic condition characterized by recurrent episodes of edema with an estimated frequency of 1:50,000 in the global population without racial or gender differences. HAE Type III is even less common, and unlike Types I and II, does not appear to be connected with the levels of C1-inhibitor. Recently three genes associated with HAE type III have been found. As long as U-HAE is present in the classification, it will encourage scientists to further searches for new genes.

**Materials and Methods:** For patients with angioedema of unclear etiology, with or without a family history, the following tests are performed according to need: measuring levels of C3c, C4, C1-INH, C1q; C1 function test and expression of C1-INH. NGS-seq amplicons of *SERPING1* gene applies to confirm HAE Types I and II. To confirm HAE Type III, the analysis of up to 201 amplicons of 18 genes using Nextera XT (Illumina) is required. All clinically significant observations are confirmed by Sanger sequencing, MLPA.

**Results:** Overall 149 patients (56.37% female; 43.63% male) were included in this analysis. For 45 patients (64.44% female; 35.56% male) from 19 unrelated families C1-INH-HAE were confirmed.

17 splicing (37.7%), 15 missense (33.3%), 8 frameshift (17.8%), 3 large del (6.7%), 2 nonsense (4.5%) mutations have been found.

It was decided to compare whether there is a pattern between the type of mutation and the expression of C1-INH. A statistically significant dependence has not been found.

**Conclusion:** Belarus is a country in Eastern Europe with a population of 9,508 million. Comparing the frequency of occurrence of HAE to the total number of population, there should be about 190 patients in the register, that is, about 76% of HAE patients are still undiagnosed. Improving the HAE diagnosis by enhancing the education of physicians and patients as well as raising awareness in the society is our main goal to the upcoming years.

The NGS-sequencing could be a useful in determining the exact genetic alteration. Using NGS technology in genetic diagnostic is much cheaper and easier than SSCP and Sanger sequencing of all exons.

### P29 A national audit of hereditary and aquired angioedema in New Zealand

#### Karen Lindsay^1,*^, Peter Flanagan^2^, Penny Fitzharris^1^, Anthony Jordan^1^

##### ^1^Auckland City Hospital, Auckland, New Zealand; ^2^New Zealand Blood service, New Zealand

###### **Correspondence:** Karen Lindsay (klindsay@adhb.govt.nz)

*Allergy, Asthma & Clinical Immunology* 2019, **15(Suppl 4):**P29

Hereditary Angioedema (HAE) is a rare potentially life threatening genetic condition, but access to effective therapies can reduce mortality and improve quality of life. Patients with this condition in New Zealand remain uncharacterized by number, geographical distribution, severity or treatment experience. New Zealand Immunologists were invited to recruit patients with HAE and AAE to the audit or those identified as having Berinert^®^ for hereditary or acquired angioedema. Participants were consented, their angioedema related health information was collected and they were invited to take part in telephone or face to face interviews about their experience of healthcare. Twenty one patients with hereditary angioedema were recruited, three of whom had acquired angioedema C1-inhibitor deficiency. Three patients were diagnosed prior to the onset of symptoms due to the diagnosis of a family member with the disease by screening family members. The average diagnostic delay was 12.9 years. Variation in delay between different types of HAE, with the greatest mean delay being in Type 1 HAE at 18.5 years, 13 years in type 2 HAE, and 3.6 years in AAE. Within the cohort of 21 patients there were reports of 4 deaths of family members due to HAE. The majority of patients 19/21 (90%) had a written plan to present to the emergency department. Few (24%) had a Medic Alert^®^ bracelet. In 2015 there were a total of 217 HAE attacks in 16 patients. Five patients (24%) were asymptomatic. Only one patient had angioedema of the upper airway in 2015 but did not require intubation. Six patients had 136 abdominal attacks; some with high frequency (range 1–52). 4 patients said HAE had no impact on their life, 10 had minor impact, and 4 moderate and 3 described it as severe. This study characterizes a cohort of AAE and HAE patients in New Zealand.

### P30 Situational analysis of diagnosis and treatment of hereditary angioedema in Latin America

#### Sandra A. Nieto^*^, José E. Fabiani, F. Alberto Contreras and workgroup for study of HAE ALaeh

##### Latin American Hereditary Angioedema Association (ALaeh)

###### **Correspondence:** Sandra A. Nieto (sandranietoaeh@gmail.com)

*Allergy, Asthma & Clinical Immunology* 2019, **15(Suppl 4):**P30

**Background:** Hereditary angioedema (HAE) is an autosomal dominant disease resulting in unpredictable attacks of swelling that can be debilitating or life-threatening for affected patients, so early diagnosis and appropriate therapy are essential. Since tests and drugs needed for diagnosis and treatment are not always available, management of this condition remains a challenge both for the treating physicians their patients. HAE affects an estimated of 1 in 50,000 individuals, although this may vary in different regions.

**Objective:** To develop a situational analysis regarding the avai lability to perform diagnostic tests and the access to treatment in patients with HAE in Latin America.

**Methods:** Cross-sectional survey in which 45 expert physicians in HAE from fourteen countries in Latin America were asked about the availability and access to diagnostic tests and treatment for hereditary angioedema in their country of origin. Three patient organizations were also included (Brazil, Chile and Mexico).

**Results:** The percentage of patients with diagnosis related to the estimated prevalence can be observed in Table 1. The diagnostic laboratory tests and the treatments available by country, at the time of the survey are shown in Tables 2 and 3, respectively.

**Table 1:** Estimated patients by population vs patients with diagnosis

Country^@^	Population 2018*	Estimated prevanlece 1:50,000	Patients diagnosed	% With diagnosis
Argentina	44,556,277	891	430	48.3
Brazil	212,664,367	4,253	578	13.6
Chile	18,433,065	368	11	2.98
Colombia	49,436,892	989	227	22.9
Costa Rica	4,945,674	99	19	19.1
Domincan Republic	10,859,463	217	22	10.1
Ecuador	16,783,322	335	5	1.49
El Salvador	6,170,519	123	8	6.5
Guatemala	17,206,382	344	3	0.87
Mexico	131,452,016	2629	296	11.2
Panama	4,092,816	82	13	15.8
Paraguay	6,863,728	137	12	8.75
Peru	32,424,843	648	15	2.31
Puerto Rico	3,669,093	73	90	123
TOTAL	559,558,457	11,188	1729	15.45

@Bolivia and Venezuela were invited but, they did not present data

*World population prospects 2017 United Nations

**Table 2:** Diagnostic laboratory tests available by country

	fC1-INH	AgC1-INH	C4	C1q	Anti-C1-INH Abs
Argentina	Y	Y	Y	Y	N
Brazil	Y	Y	Y	Y	Y
Chile	Y	Y	Y	Y	N
Colombia	Y	Y	Y	Y	N
Costa Rica	Y	Y	Y	N	N
Domincan Republic	Y	Y	Y	N	N
Ecuador	Y	Y	Y	Y	N
El Salvador	N	N	Y	N	N
Guatemala	N	N	Y	Y	N
Mexico	Y	Y	Y	Y	N
Panama	Y	N	Y	N	N
Paraguay	N	N	Y	N	N
Peru	Y	N	Y	Y	N
Puerto Rico	Y	Y	Y	Y	Y

fC1-INH C1-inhibitor function, AgC1-INH C1-inhibitor concentration (antigen), Anti-C1-INHB Ab autoantibodies against C1-inhibitor, Y yes, N no

**Table 3:** Treatments available by country

	Berinert^®^	Cynryze^®^	Firazyr^®^	Ruconest^®^	Kalbitor^®^	LMWH*	Tranexamic acid	FFP**	Danazol
Argentina	Y	N	Y	N	N	N	Y	Y	N
Brazil	Y	N	Y	N	N	N	Y	Y	N
Chile	Y	N		N	N	Y	Y	Y	N
Colombia	Y	N	Y	N	N	N	N	Y	N
Costa Rica	N	N	N	N	N	N	N	Y	N
Domincan Republic	N	N	N	N	N	N	Y	Y	Y
Ecuador	N	N	N	N	N	N	Y	Y	N
El Salvador	N	N	N	N	N	Y	Y	Y	N
Guatemala	N	N	N	N	N	Y	Y	Y	Y
Mexico	Y	N	Y	N	Y	Y	Y	Y	Y
Panama	N	N	N	Y	N	Y	Y	Y	N
Paraguay	N	N	N	N	N	Y	N	Y	Y
Peru	N	N	N	N	Y	N	Y	Y	N
Puerto Rico	N	Y	N	Y	Y	Y	Y	Y	Y

*Low molecular weight heparins, **Fresh frozen plasma, Y yes, N no

**Conclusion:** In Latin America, the availability diagnostic tests of HAE seem to be related to the degree of development of the country and treatment depends of the health laws of each. In some countries first-line treatments are not offered to all patients and even non recommended treatments continue to be used, despite the known adverse effects. The need for better access to diagnosis and treatment of HAE in Latin American patients cannot be underestimated.

### P31 Hereditary angioedema in Belarus: epidemiology, clinical characteristics and access to diagnosis and treatment

#### Irina E. Guryanova^1^, Nastassia Ishchanka^1^, Mikhail V. Belevtsev^1^, Katsiaryna A. Paliakova^1^, Antonio Gidaro^2^, Chiara Suffritti^3^, Sonia Caccia^3^, Marco Cicardi^2,3^, Francesca Perego^4,*^

##### ^1^Belarussian research center for pediatric oncology, hematology and immunology, Minsk, Belarus; ^2^General Medicine Department, ASST-Fatebenefratelli-Sacco, Milan, Italy; ^3^Department of Biomedical and Clinical Sciences Luigi Sacco, University of Milan, Milan, Italy; ^4^Istituti Clinic Scientifici Maugeri IRCCS, Milan, Italy

###### **Correspondence:** Francesca Perego (francescappe@gmail.com)

*Allergy, Asthma & Clinical Immunology* 2019, **15(Suppl 4):**P31

**Background:** Hereditary angioedema due to C1-inhibitor deficiency (C1INH-HAE) is a rare disease caused by deficiency of complement C1-INH. Only few states in developing countries have an adequate management of HAE, but none of them belongs to the former USSR area. This study analyses data from C1INH-HAE patients from Belarus, as example of the region.

**Materials and methods:** In Belarus were identified 3 referral centres and 2 assosiations for the evaluation of patients with HAE (Belarussian Research Center for pediatric oncology, hematology and immunology (Jeffrey Modell Belarussian Center for primary immunodeficiency)(Minsk); Health Care Institution with 4 City Children’s clinical Hospital and 10 City clinical Hospital (Minsk); Republican Scientific Center for Radiation Medicine and Human Ecology (Homel); Republican Association of parents of patients with primary immunodeficiency “save the immunity”; republican association “help HAE patients”. Clinical characteristics and access to diagnosis and treatment were collected from 2014 by the Belarusian Research Centre for Paediatric Oncology, Haematology and Immunology in Minsk. A questionnaire about disease’s severity, prophylactic and on-demand therapy was administered to patients. Data about attacks refers to year 2016.

**Results:** We identified 37 C1-INH-HAE patients, 22 female (59%); 35 type 1 (95%), 2 type 2 (5%) HAE; mean age 31 years (min 4- max 67- median 32); mean age at onset of symptoms 11y (min 1-max 63 y) and a median age at the diagnosis of 29 y (min 1-max 63 y), with a diagnostic delay of 18y. Mean levels of C1-INH and C4 were 0.04 g/L and 0.03 respectively. The estimated prevalence was 1:255000. Twenty patients accepted to compile the questionnaire and to collect data about attacks. 271 attacks were reported with an attacks mean/patients of 16 (min 3, max75, median 22). 181 were peripheral, 111 abdominal, laryngeal attacks were not reported separately. 8 patients defined their disease severity as severe, 8 moderate, 4 mild. 7 patients used on-demand therapy during attacks (2 patients used fresh-frozen plasma, 2 C1-INH concentrate, 2 icatibant, 1 tranexamic) although C1-INH concentrate nor icatibant are registered; 5 patients used prophylactic therapy with attenuated androgens.

**Conclusions:** In Belarus the possibility to diagnose and manage patients with C1-INH-HAE is scarce, effective on-demand treatments for acute attacks are still lacking. Nonetheless the growing knowledge of the disease, the registration of patients associations among the Ministry of Health and the identification and development of center of reference are the first steps to guarantee patients prompt diagnosis and adequate treatment.

### P32 Comparison of the C1-INH productions of different endothelial cells

#### Erika Kajdácsi^1,*^, Lilian Varga^1,2^, László Cervenak^1^

##### Research Laboratory, 3^rd^ Department of Internal Medicine, Semmelweis University, Budapest, Hungary; ^2^ Hungarian Angioedema Reference Center, 3^rd^ Department of Internal Medicine, Semmelweis University, Budapest, Hungary

###### **Correspondence:** Erika Kajdácsi (erika.kajdacsi@gmail.com)

*Allergy, Asthma & Clinical Immunology* 2019, **15(Suppl 4):**P32

Endothelial cells (ECs) play a key role in edema formation. In the case of hereditary angioedema (C1-INH-HAE), the edema formation is the consequence of the permeability increasing effect of the elevated bradykinin (BK) level. Because of this key role of ECs in the edema formation, the question emerges: can ECs somehow downregulate the permeability increasing effect of BK?

Although C1-INH production of endothelial cells was reported, the data are not consistent and sometimes are controversial. Since different tissues behave quite diversely during edematous attacks, we aimed to map the C1-INH producing capabilities of various ECs. We studied the regulation of C1-INH production by several potential factors – known to trigger edematous attacks- on endothelial cells.

We measured the C1-INH mRNA production of primary ECs, such as human umbilical vein and arterial ECs (HUVECs and HUAECs), human dermal microvascular ECs (HDMECs) and human glomerular ECs (GECs), as well as of EC line, human brain microvascular ECs (HCMEC-D3), and of HepG2 cell line as a positive control. After this we used different stimuli: thrombin (TR), BK, TGF-beta, interferon gamma (IFNg), and TR and BK together, and measured the change of mRNA and protein levels of C1-INH after 24 and 48 h. We used qPCR to measure mRNA levels, and an in-house ELISA to detect C1-INH protein production.

We found that all investigated ECs can produce C1-INH at mRNA level. Moreover, HUVEC and HDMEC produced and secreted C1-INH into the cell culture supernatant (2.41 ± 0.34 ng/10^5 cells for HUVEC, 0.496 ± 0.018 ng/10^5 cells for HDMEC). IFNg treatment caused a significant increase in the expression of C1-INH at both mRNA and protein level in HUVECs and in HDMECs. Although TGF-beta, and BK together with TR or TNF, and in some HUVEC lines BK alone could also induced the expression of C1-INH, these changes were minor compared to the effect of IFNg.

All endothelial cell types produced C1-INH, which suggests that ECs can actively regulate the plasma serine protease cascades, thus the pathophysiological process of angioedema.

The difference between C1–INH expression upon stimulation with several potential trigger factors highlights that initiation routes of HAE attacks may implicate distinct predisposition for edema formation as well as to distinct efficiency to resolve the attacks. Taken together, we propose that the pathophysiology of HAE attacks may depend on the integrative function of bradykinin metabolism, C1-INH metabolism and the actual phenotype of endothelial cells.

Supported by OTKA 112110.

### P33 Sensitive assays for measuring C1-inhibitor

#### Zsófia Jandrasics^1,*^, Erika Kajdácsi^1^, Nóra Veszeli^1,2^, Kinga Viktória Kőhalmi^1,3^, Dominik Gulyás^1^, László Cervenak^1^, Péter Gál^4^, József Dobó^4^, Henriette Farkas^1,3^, Lilian Varga^1,3^

##### Research Laboratory, 3^rd^ Department of Internal Medicine, Semmelweis University; ^2^ MTA-SE Research Group of Immunology and Hematology, Hungarian Academy of Sciences and Semmelweis University, Budapest, Hungary; ^3^ Hungarian Angioedema Reference Center, 3^rd^ Department of Internal Medicine, Semmelweis University, Budapest, Hungary; ^4^ Institute of Enzymology, Research Centre for Natural Sciences, Hungarian Academy of Sciences, Budapest, Hungary

###### **Correspondence:** Zsófia Jandrasics (zsofi.j11@gmail.com)

*Allergy, Asthma & Clinical Immunology* 2019, **15(Suppl 4):**P33

Measurement of C1-inhibitor (C1-INH) levels has diagnostic importance. The commercially available diagnostic tests fail to fulfill all requirements for research purposes, therefore, we developed proprietary methods to determine the antigenic concentration and functional activity of the C1-INH protein.

We used affinity-purified, polyclonal anti-C-INH IgG, biotinylated anti-C1-INH, and biotinylated recombinant C1s. Plasma-derived, active C1-INH was chosen as standard, the concentration was measured by spectrophotometry. We developed an antigenic ELISA (AgELISA) and a complex based assay for functional activity (FELISA). Both methods were tested on blood samples from symptom-free C1-INH-HAE patients (AgELISA 33, FELISA 60). The results were compared to those obtained with commercially available kits of a similar principle (by Molecular Innovation [MI] and Quidel [Q]). Further, we measured C1-INH levels of 6 controls, as well as of 5–5 symptom-free patients with hereditary angioedema (C1-INH-HAE) type I or type II– 5 samples were obtained during edematous attack by AgELISA and FELISA. We also monitored the changes of C1-INH levels during the spontaneous course of an edematous attack in a type I patient, from onset to resolution.

The concentrations measured with AgELISA and MI (expressed as g/L) correlated with each other (Spearman’s r = 0.4689; *p *= 0.0059), and the results of the two tests were not significantly different. Activity determined with FELISA closely correlated with that determined with the percentage values measured with the Q test (Spearman’s r = 0.8982; *p *< 0.0001).

The concentrations measured with AgELISA in type II patients and in the controls were similar; however, activity levels were lower in the patients. In type I HAE, the activity/concentration ratio was not different from that seen in controls; however, it was the lowest in type II C1-INH-HAE. C1-INH levels were similar between samples obtained during attacks or during symptom-free periods. Kinetic monitoring of an edematous attack revealed that the activity/concentration ratio was the lowest (56%) at the onset of edematous symptoms, owing to the reduced activity measured at this time (0.018 g/L).

We showed for the first time that absolute activity and activity/concentration ratio of C1-INH touches a bottom at the onset, but some improvement occurs at the peak of the symptoms. In contrast with kinetic monitoring, C1-INH levels measured during symptom-free periods or during attacks were not different in the cross-sectional study of the patients. This may be explained by the variability among individuals and edematous attacks, as well as by the differences in time between symptom onset and blood sampling.

**Supported by:** OTKA 112110.

### P34 Increased fibrinolysis-induced bradykinin formation in hereditary angioedema confirmed using stored plasma and biotechnological inhibitors

#### François Marceau^1,*^, Hélène Bachelard^1^, Georges-Étienne Rivard^2^, Jacques Hébert^3^

##### ^1^Axe Microbiologie-Infectiologie et Immunologie, Research Center, CHU de Québec-Université Laval, Québec, QC, Canada; ^2^Hematology-Oncology, CHU Ste-Justine, Montreal, QC, Canada; ^3^Service d’allergie, CHU de Québec-Université Laval, Québec, QC, Canada

###### **Correspondence:** François Marceau (francois.marceau@crchudequebec.ulaval.ca)

*Allergy, Asthma & Clinical Immunology* 2019, **15(Suppl 4):**P34

**Background:** We recently investigated the pathways of immunoreactive bradykinin (iBK) formation in fresh blood of normal volunteers and of patients with hereditary angioedema due to C1-esterase inhibitor deficiency (HAE-1/-2) [1]. Since we did not detect iBK formation following platelet or neutrophil activation in whole blood, we adapted the techniques to small volumes (200 μl) of previously frozen plasma and further analyzed the mechanisms of iBK formation with additional biotechnological inhibitors.

**Materials and methods:** Each experimental point was obtained using 200 μl of thawed citrated plasma transferred to a 1.5 ml conical test tube; activators or inhibitors were added to test various pathways of kinin generation. All tubes contained enalaprilat to protect BK from rapid inactivation. The tubes were incubated under rotary agitation in a pre-equilibrated (37 °C) Thermomixer apparatus. The ethanol extraction, sample evaporation and enzyme immunoassay were then performed as described [1].

**Results:** iBK formation was observed under stimulation with tissue kallikrein (KLK-1, 10 nM), the particulate material Kontact-APTT (concentration reduced to 2% v/v) or recombinant tissue plasminogen activator (tPA, 169 nM), with little background in unstimulated plasma incubated for up to 2 h. Plasma samples from HAE-1/-2 patients responded earlier to tPA than those from controls, as previously reported with whole blood. Lanadelumab inhibited iBK formation induced by Kontact-APTT and tPA. A highly specific plasmin inhibitor, DX-1000, abolished tPA-induced iBK formation in plasma but had no effect against Kontact-APTT, confirming the role of fibrinolysis in tPA-induced kinin formation. The anti-lanadelumab neutralizing antibody M293-D02 reversed the inhibitory effects of lanadelumab.

**Conclusions:** Frozen plasma is a suitable material for measuring iBK formation kinetics, with possible applications such as investigating the effect of rare disease states on the kallikrein-kinin system and monitoring the effect of HAE prophylactic treatments. The instability of the kallikrein-kinin system in HAE-1/-2 may reside in the upstream fibrinolytic system. Common attack triggering factors are compatible with this hypothesis.

**Acknowledgements:** Supported by Shire, now part of the Takeda group of companies (IIR-CAN-001615). We thank Dr. D.J. Sexton for useful interaction and the gift of inhibitors.

**Consent to publish:** The CHU de Québec-Université Laval ethical review board approved the study (file no. 2018-3857). All study subjects gave written informed consent.

**Reference**

1. Charest-Morin X, Hébert J, Rivard GÉ, Bonnefoy A, Wagner E, Marceau F. Comparing pathways of bradykinin formation in whole blood from healthy volunteers and patients with hereditary angioedema due to C1-inhibitor deficiency. Front Immunol. 2018; 9: 2183.

### P35 High plasma exposures of KVD900 achieved in First in Human study markedly inhibit plasma prekallikrein activation; early blockade of plasma kallikrein (PKa) may halt attacks in hereditary angioedema (HAE) by reducing contact system activation

#### Edward Duckworth^1,*^, Sally Hampton^1^, Nivetha Murugesan^2^, Lilly Li^2^, Gian Marco De Donatis^1^, Louise Rushbrooke^1^, Michael Smith^2^, Rachel Morten^1^, Andreas Maetzel^2^, Chris Yea^1^, Edward Feener^2^

##### ^1^KalVista Pharmaceuticals, Salisbury, United Kingdom; ^2^KalVista Pharmaceuticals, Cambridge, MA, United States

###### **Correspondence:** Edward Duckworth (ejd@kalvista.com)

*Allergy, Asthma & Clinical Immunology* 2019, **15(Suppl 4):**P35

**Introduction:** Early treatment of acute attacks in HAE is associated with improved clinical outcomes. These clinical benefits are attributed to the reduction of plasma kallikrein mediated high molecular weight kininogen (HK) cleavage and bradykinin action. The effects of PKa inhibition on contact system activation has received relatively little attention. This study examines the effects of KVD900, a rapidly acting oral PKa inhibitor with high clinical exposure, on plasma prekallikrein cleavage and PKa activity during contact system activation.

**Methods:** Single ascending doses of KVD900, a potent and selective small molecule plasma kallikrein inhibitor, were administered orally using a powder-in capsule formulation to healthy adult males. Pharmacodynamic measurements were determined in dextran sulphate (DXS) stimulated whole plasma using a fluorogenic enzyme assay to measure PKa catalytic activity. A capillary based immunoassay was used to measure plasma prekallikrein and HK cleavage following DXS stimulated contact system activation.

**Results:** Kinetic enzyme assays on whole plasma demonstrated that the orally administered 600 mg capsules of KVD900 achieved > 98% inhibition of DXS-stimulated PKa activity between 90 min and 3 h and provided > 90% inhibition of PKa catalytic activity between 30 min and 6 h post-dose. Moreover, the time for detectable DXS-stimulated PKa activity (lag time) was prolonged > 5 fold in plasma samples at 30 min post-dose, suggesting that KVD900 rapidly prevented the generation of PKa from plasma prekallikrein. Immunoassay analysis of plasma prekallikrein confirmed that KVD900 reduced DXS-induced plasma prekallikrein activation from 60.5% in pre-dose to < 20% at 1 h and 6 h post dose. Reduced plasma prekallikrein activation correlates with protection of HK from cleavage. The tablet formulation to be used in upcoming clinical studies achieves effective concentrations in less than 30 min, rapidly reaching complete PKa inhibition, and maintains > 85% PKa inhibition for up to 10 h.

**Conclusions:** Orally administered 600 mg KVD900 achieves rapid plasma exposure sufficient to inhibit the formation of active PKa from plasma prekallikrein during contact system activation. These results suggest a mechanism to halt HAE attacks following early administration of KVD900.

### P36 The treatment of angioedema attacks by bradykinin B2-receptor antagonist icatibant in hereditary angioedema – a real-life study

#### Noémi Andrási^1,3,*^, Nóra Veszeli^1,2^, Kinga Viktória Kőhalmi^1^, György Temesszentandrási^4^, Lilian Varga^1^, Henriette Farkas^1^

##### ^1^Hungarian Angioedema Reference Center, 3^rd^ Department of Internal Medicine, Semmelweis University, Budapest, Hungary; ^2^MTA-SE Immunology & Hematology Research Group, Semmelweis University and Hungarian Academy of Sciences, Budapest, Hungary; ^3^2^nd^ Department of Pediatrics, Semmelweis University, Budapest, Hungary; ^4^3^rd^ Department of Internal Medicine, Semmelweis University, Budapest, Hungary

###### **Correspondence:** Noémi Andrási (nono.andrasi@gmail.com)

*Allergy, Asthma & Clinical Immunology* 2019, **15(Suppl 4):**P36

**Objective:** The synthetic peptide icatibant – a bradykinin B2-receptor antagonist administered subcutaneously– is intended for the acute treatment of angioedema attacks in patients with hereditary angioedema with C1-inhibitor deficiency (C1-INH-HAE). Our study analyzed the efficacy and the adverse effects of icatibant, as well as patient satisfaction with treatment.

**Materials and methods:** We analyzed 546 angioedema attacks experienced by 40 C1-INH-HAE patients. By completing a questionnaire, the patients recorded treatment-related information. The severity of the individual angioedema attacks was graded. The patients also recorded any adverse effects, and rated their satisfaction with the treatment.

**Results:** The distribution of the analyzed attacks: 278 subcutaneous, 178 gastrointestinal, 8 upper airway, and 82 multiple locations. Icatibant was injected at a median interval of 65.0 min after the onset of the angioedema attack, the severity of which was 66 (median), according to the visual analog scale (VAS). The symptoms started to improve 35.0 min (median) and resolved 420.0 min (median) after treatment. The time between the administration of the injection and the resolution of the attack was correlated with time until injection (r = 0.2322, p < 0.0001). On 39 occasions, the symptoms failed to improve or to resolve after the administration of a single dose. A second dose was administered in 23/39 instances, and this eliminated the symptoms in 20/23. The angioedema attack recurred in 9.3% of the instances (n = 51). We did not find any significant difference in time to injection between icatibant-responsive and non-recurrent *vs*. rebound attacks. However, the latter occurred in 16.32% of instances when the time to injection was 30 min or less, and in 7.71% when it was longer than 30 min. Thirty-three patients experienced a skin reaction at the injection site. On average, satisfaction with the treatment was 88.65 on a 100-mm VAS, and a mean score of 9.05 was assigned on a scale of 1 to 10.

**Conclusion:** Icatibant is an effective and safe medicine with a rapid onset of action in C1-INH-HAE. We can conclude that early treatment resulted in quicker resolution of the symptoms, however 30 min or less administration time showed higher percent of rebound attacks. Local skin reactions were common, but possibly drug-related systemic adverse effects did not occur.

This study was supported by OTKA K124557.

### P37 Evaluation of the efficacy and safety of home treatment with the recombinant human C1-inhibitor in hereditary angioedema

#### Noémi Andrási^1,3,*^, Ágnes Holdonner^1^, Nóra Veszeli^1,2^, Kinga Viktória Kőhalmi^1^, György Temesszentandrási^4^, Lilian Varga^1^, Henriette Farkas^1^

##### Hungarian Angioedema Reference Center, 3^rd^ Department of Internal Medicine, Semmelweis University, Budapest, Hungary; ^2^ MTA-SE Immunology & Hematology Research Group, Semmelweis University and Hungarian Academy of Sciences, Budapest, Hungary; ^3^ 2^nd^ Department of Pediatrics, Semmelweis University, Budapest, Hungary; ^4^3^rd^ Department of Internal Medicine, Semmelweis University, Budapest, Hungary

###### **Correspondence:** Noémi Andrási (nono.andrasi@gmail.com)

*Allergy, Asthma & Clinical Immunology* 2019, **15(Suppl 4):**P37

**Objective:** Conestat alpha, a C1-inhibitor produced by recombinant technology (rhC1-INH) is an acute treatment for angioedema attacks in hereditary angioedema with C1-inhibitor deficiency (C1-INH-HAE). Our study evaluated the efficacy and safety of rhC1-INH administered during angioedema attacks, and for short-term prophylaxis (STP).

**Material and method:** Our prospective study analyzed the course of 544 angioedema attacks experienced by 21 C1-INH-HAE patients treated – as well as the outcome of 69 instances of STP implemented – with rhC1-INH. Using a purpose-designed questionnaire, the patients recorded treatment-related information. Furthermore, the severity of the individual attacks was graded on a 100-mm visual analog scale (VAS). The patients also recorded any adverse events, and rated their satisfaction with the treatment.

**Results:** The 544 angioedema attacks comprised 240 subcutaneous, 217 gastrointestinal, 14 upper-airway attacks, and 73 multiple locations. Time to the administration of rhC1-INH was 90.0 min (median) after the onset of the attacks, the severity of which was 60 (median) on a VAS. The symptoms started to improve as early as 60 min (median) after the injection of rhC1-INH, and the attack resolved 730.0 min (median) after treatment. The interval between the onset of the attack and the administration of rhC1-INH correlated with time to cessation of the worsening of symptoms (R = 0.171, p < 0.0001), as well as with time until the onset of improvement (R = 0.2053 p < 0.0001), and with time to the complete resolution of symptoms (R = 0.2805, p < 0.0001). In 16 HAE attacks, administering a second dose was necessary – and a third injection was required in one attack. Nine patients received STP with rhC1-INH in 98 instances, including minor surgery, stressful situations, fatigue, change of the weather, or travel. Furthermore, prophylaxis was administered because of the occurrence of erythema marginatum in 79 instances – STP failed to prevent the angioedema attack on five occasions. The repeated administration of rhC1-INH did not reduce its efficacy. The time to the resolution of the angioedema attack was not significantly different among the instances of administering rhC1-INH to the same patient. Satisfaction rate was 93.36 (VAS) on average.

**Conclusions:** Treatment with rhC1-INH is effective both for the management of acute angioedema attacks and for STP. Following the onset of attack, early administration of this drug may reduce time to the improvement and to the complete resolution of symptoms. Repeated administration of rhC1-INH does not impair its efficacy. This treatment is well tolerated and it is devoid of systemic adverse effects.

This study was supported by OTKA K124557.

### P38 Characterizing the minority of hereditary angioedema attacks that require more than a single injection of icatibant

#### Irmgard Andresen^1,*^, Marcus Maurer^2^, Hilary J. Longhurst^3^, Laurence Bouillet^4^, Teresa Caballero^5^, Andrea Zanichelli^6^, Anete S. Grumach^7^, Jaco Botha^1^, Werner Aberer^8^

##### ^1^Shire, a Takeda company, Zug, Switzerland; ^2^Department of Dermatology and Allergy, Allergie-Centrum-Charité, Charité–Universitätsmedizin Berlin, Berlin, Germany; ^3^Department of Clinical Biochemistry and Immunology, Cambridge University Hospitals NHS Foundation Trust, Cambridge, United Kingdom; ^4^National Reference Centre for Angioedema, Internal Medicine Department, Grenoble University, Grenoble, France; ^5^Department of Allergy, Hospital La Paz Institute for Health Research (IdiPaz), Biomedical Research Network on Rare Diseases (CIBERER, U754), Madrid, Spain; ^6^Department of Biomedical and Clinical Sciences Luigi Sacco, University of Milan, ASST Fatebenefratelli Sacco, Milan, Italy; ^7^Faculdade de Medicina ABC, São Paulo, Brazil; ^8^Department of Dermatology and Venereology, Medical University of Graz, Graz, Austria

###### **Correspondence:** Irmgard Andresen (irmgard.andresen@takeda.com)

*Allergy, Asthma & Clinical Immunology* 2019, **15(Suppl 4):**P38

**Background:** The Icatibant Outcome Survey (IOS; NCT01034969), an ongoing international observational registry, monitors the safety and effectiveness of icatibant, a bradykinin B2 receptor agonist approved for the treatment of hereditary angioedema with C1-inhibitor deficiency (C1-INH-HAE) attacks. Findings from the IOS have shown most C1-INH-HAE attacks are successfully treated with one injection of icatibant. In this analysis, we evaluated reinjection data from more than 6000 attacks to determine the characteristics of HAE attacks that required icatibant reinjection.

**Methods:** Descriptive, retrospective analyses were conducted on data obtained from patients reporting ≥ 1 icatibant-treated attack between July 2009 and January 2019 to determine patient and attack characteristics associated with icatibant reinjection. For these analyses, patients were stratified by number of icatibant injections administered per attack.

**Results:** Included in this analysis were 501 patients with C1-INH-HAE (HAE type I and II; 58.9% females) who had 6047 icatibant-treated attacks. Icatibant was self-administered in 94.5% of attacks. The majority of attacks (93.3%) were treated with a single injection of icatibant. Of 405 attacks requiring icatibant reinjection, 224 (55.3%) were abdominal attacks and 197 (48.6%) were severe or very severe (in any location). Few laryngeal attacks (21/225 [9.3%]) required reinjection. Reinjection was associated with longer median attack duration. The second injection was administered > 12 h after the first dose in most attacks (86.3%). BMI (< 25 kg/m^2^ vs ≥ 25 kg/m^2^) was not a significant predictive factor for icatibant reinjection (*P *= 0.169). There was no significant difference for C1-INH rescue medication use after one icatibant injection (5.8%) versus more than one injection (5.4%; *P *= 0.751).

**Conclusions:** Reinjection was required in less than 10% or 1 in 10 attacks treated with icatibant. Abdominal attacks and those that were severe or very severe were most likely to require reinjection. Most patients requiring more than one dose waited at least 12 h after the first dose to reinject.

**Trial registration:** The Icatibant Outcome Survey, NCT01034969

### P39 Long–term prophylaxis in hereditary angioedema patients followed in ITAlian Centers for Angioedema (ITACA) and enrolled in HAE Global Registry (HGR)

#### Francesco Arcoleo^1^, Mauro Cancian^2,*^, Marco Cicardi^3^, Tiziana De Pasquale^4^, Ruggero Di Maulo^5^, Domenica Maria Guarino^6^, Mariangela Lo Pizzo^1^, Riccardo Senter^2^, Paolina Quattrocchi^7^, Carmelo Umina^1^, Andrea Zanichelli^3^

##### ^1^Department of Clinical Pathology Cervello Hospital of Palermo, Palermo, Italy; ^2^Department of Medicine University of Padua, Padua, Italy; ^3^Department of Biomedical and Clinical Sciences Luigi Sacco Milan, Milan, Italy; ^4^Department of Allergology Hospital of Civitanova Marche, Civitanova Marche, Italy; ^5^Cloud-R-Srl Milan, Milan, Italy; ^6^Department of Rheumatology Policlinico Tor Vergata Rome, Rome, Italy; ^7^Department of Medicine, Allergology and Immunology Policlinico of Messina, Messina, Italy

###### **Correspondence:** Mauro Cancian (mcancian@unipd.it)

*Allergy, Asthma & Clinical Immunology* 2019, **15(Suppl 4):**P39

Long-term prophylaxis (LTP) is targeted to prevent the recurrence of acute attacks in patients affected by hereditary angioedema due to C1-inhibitor deficiency (C1-INH-HAE). Approved for LTP in Italy are plasma derived C1-INH (pdC1-INH), attenuated androgens (danazol/stanozolol), tranexamic acid. According to guidelines, patients start LTP when disease control is not satisfactory with on-demand treatment.

This study aims at evaluating prevalence and efficacy of LTP at ITACA centers.

Four hundred and four patients with C1-INH-HAE (mean age 51.2 yo, range 8 to 88 yo) at 6 ITACA centers are listed in the HGR, a multicenter disease registry (ClinicalTrials.gov NCT03828279). The study considered patients on stable LTP at July 1, 2018. ITACA policy for starting LTP is presence of more than one attack per month. All patients in the study recorded angioedema attacks prospectively; the number of attacks between July-December 2018 was analyzed. Primary efficacy endpoint was number of attack-free days. Side effects were recorded during the observation period.

Ninety nine patients of 404 (24.5%) were on LTP (61 F, 38 M); 60 with danazol, 9 with stanazolol, 22 with pdC1-INH and 8 with tranexamic acid. Androgen posology was highly variable, from 200 to 2800 mg/week (mean 860) for danazol and from 4 to 14 mg/week for stanozolol (mean 9.2). The mean dose for pdC1-INH was 1985 IU/week (from 1000 IU to 6000 IU/week). Tranexamic acid dose fluctuated between 0.7 and 21 g/week, (mean 6.54 g/week). 38/60 patients (63.3%) on danazol LTP were attack-free throughout the 6 months observation period, the remaining 22 (36.6%) had 139 HAE attacks, with a mean frequency of 6.3 attacks/semester ranging from 1 to 30 attacks/semester. One of 9 patients on stanozolol was attack-free the 8 remaining had 13 angioedema episodes (range: 1–3/semester; mean = 1.6/semester). PdC1-INH LTP was fully effective in preventing the recurrence of angioedema in 11 patients (50%), whereas 11 experienced a total of 95 attacks (range: 1–36/semester; mean = 8.6/semester). Finally, 3/8 patients (37.5%) treated with tranexamic acid were attack-free and 5 had total of 20 attacks in the semester (range 1–9; mean = 4).

**Conclusions:** In our population, attenuated androgens represent the main approach to LTP. All LTP approaches are effective (attacks reduction over 50%) in the large majority of patients. Pooling patients’ data in HGR allows rapid evaluation of drug efficacy, improves knowledge on the disease and facilitates treatment personalization.

### P40 Shortage in France of plasma derived C1-INH concentrates: state of play and consequences for patients

#### Laurence Bouillet^*^, Isabelle Boccon-Gibod, David Launay, Delphine Gobert, Anne Du Than, Yann Olliver, Guillaume Armengol, Fabien Pelletier, Pierre Yves Jeandel, Claire Demoreuil, Stephane Gayet, Stephane Guez, Sophie Debort, Oliver Fain from the CREAK

##### CREAK, Internal Medicine/Clinical Immunology Department, Grenoble University Hospital, Grenoble, France

###### **Correspondence:** Laurence Bouillet (lbouillet@chu-grenoble.fr)

*Allergy, Asthma & Clinical Immunology* 2019, **15(Suppl 4):**P40

**Introduction:** In France, several treatments are available for Hereditary angioedema: two plasma derived C1Inh (Berinert, Cinryze), a recombinant C1Inh (Ruconest), Firazyr, danazol and tranexamic acid. Since 2017, France has been experiencing a shortage of blood products that affect C1Inh concentrates. This shortage has triggered a health crisis that has altered the care of patients. The CREAK (National Reference Center for Angioedema) and the ANSM (National Agency for Health and Drugs) have had to take exceptional measures, including those to offer Ruconest, including pregnant women and children, as well as long term prophylaxis.

**Methods:** CREAK conducted a survey within its network and referring physicians to assess the impact of this health crisis.

**Results:** The shortage induced a therapeutic change in 61 patients, half of whom had a very severe illness and one-third a severe illness. For all these patients, it was a modification of their long term prophylaxis with C1Inh concentrate (Cinryze or Berinert). In 70% of cases, patients were switched to Ruconest with an imbalance of pathology in 40% of cases. 40% of doctors could not apply international recommendations on short-term prophylaxis. 5 pregnant women had difficulties of care because of this shortage. The doctors appreciated the help of CREAK in 80% of the cases and followed its recommendations. The image of laboratories has been altered with 70% of doctors who no longer trust them. 70% of physicians consider that the lives of their patients have been jeopardized.

**Conclusion:** The shortage of plasma derived C1Inh has impaired patients’ quality of life and sometimes put their lives at risk. CREAK and ANSM had to advocate off-label uses of certain products. Since October 2018, France has obtained the availability of lanadelumab for these severe patients in order to depend less on plasma derived C1Inh. The first encouraging results give new hope to patients and doctors.

### P41 Limelight on erythema marginatum: a review of clinical features and the introduction of a new management strategy in hereditary angioedema

#### Ágnes Holdonner^*^, Kinga Viktória Kőhalmi, Henriette Farkas

##### Hungarian Angioedéma Reference Center, Semmelweis University, 3^rd^ Department of Internal Medicine, Budapest, Hungary

###### **Correspondence:** Ágnes Holdonner (holdonnera@gmail.hu)

*Allergy, Asthma & Clinical Immunology* 2019, **15(Suppl 4):**P41

**Objective:** To assess the incidence and clinical characteristics of erythema marginatum (EM) along with the efficacy of the therapies administered during episodes of EM to patients receiving follow-up care for C1-inhibitor-deficient hereditary angioedema at the Hungarian Angioedema Reference Center.

**Materials and methods:** During the first stage of our study, we surveyed the incidence of EM using the Erythema Marginatum Basic Questionnaire (EMBQ) developed by our team. In the second stage, we evaluated therapeutic efficacy with the Erythema Marginatum Detailed Questionnaire (EMDQ).

**Results:** According to the EMBQ, 72 out of 134 C1-INH-HAE patients from 60 families (mean age 40 years, min.: 6 years, max.: 82 years) experienced EM during their lifetime. Forty-three of these 72 patients were females (59.7%). In 41.7% of families with C1-INH-HAE, EM occurred in all family members, whereas 20% of the families have never experienced this skin lesion. In these 72 patients, EM appeared in the following locations: on the upper extremities in 79.2%, on the chest in 63.9%, on the lower extremities in 33.3%, on the back in 25%, in the abdominal region in 19.4%, and on the face in 12.5%. EM first occurred at the age of 17 years on average, and as an isolated cutaneous lesion in 47.2% of the patients. EM was followed by an acute episode of hereditary angioedema in 8 out of 10 instances. As shown by the EMDQ, 16 C1-INH-HAE patients (15 females and 1 male) administered the following treatments on 160 occasions: plasma-derived C1-INH concentrate (pdC1-INH – n = 73), recombinant C1-INH concentrate (rhC1-INH – n = 79), and icatibant (n = 8). The onset of the HAE attack was prevented by pdC1-INH in 94.5%, by rhC1-INH in 93.6% and by icatibant in 50% of cases.

**Conclusion:** EM is characterized by a variegated clinical picture, as regards both the time of the onset and the location of symptoms. Being an objective prodromal sign, its occurrence creates an opportunity to administer acute treatment for HAE attacks earlier and thereby to prevent their onset. This new therapeutic strategy is cost-effective, and it might significantly improve the patients’ quality of life.

This study was supported by OTKA K124557.

### P42 Hereditary angioedema: dental management in a Brazilian reference center

#### Solange Oliveira Rodrigues Valle^1^, Bernardo Correia Lima^2^, Maria Luiza Oliva Alonso^1,*^, Cláudia de San Thiago Ragon^3^, Alessandra Oliveira Ferrari^3^, Sandra Regina Torres^2,3^, Michelle Agostini^2,3^

##### Federal University of Rio de Janeiro, HUCFF, Department of Internal Medicine, Clinical Immunology Service, RJ, Brazil; ^2^ Federal University of Rio de Janeiro, Department of Oral Pathology and Diagnosis, School of Dentistry, RJ, Brazil; ^3^ Federal University of Rio de Janeiro, HUCFF, Oral Health Special Program, RJ, Brazil

###### **Correspondence:** Maria Luiza Oliva Alonso (mloalonso@yahoo.com.br)

*Allergy, Asthma & Clinical Immunology* 2019, **15(Suppl 4):**P42

**Objective:** To describe the dental management and oral health status of nine patients with hereditary angioedema (HAE).

**Methods:** Demographic data, type and severity of HAE, long and short-term prophylaxis, number of HAE attacks following dental procedures were retrieved from the files of patients submitted to dental procedures in a University Hospital from Brazil from 2012 to 2017. Number of teeth and presence of bone loss were evaluated by panoramic radiographs.

**Results:** Nine patients were enrolled, all in use of long-term prophylaxis, most with attenuated androgens (AA); most patients (78%) were women, with HAE-C1INH type I and mean age of 47 years. Five patients (58%) showed signs of periodontal disease. Forty-three dental procedures were performed, the most common being tooth extractions, dental restorations and supra-gingival removal of calculus. Six patients (67%) were submitted to dental procedures without modification of long-term prophylaxis, whereas an increased dose of AA and/or C1-INH concentrate was administrated before the dental procedures in three patients (33%), especially in those undergoing dental extractions. Angioedema attacks were not observed.

**Conclusion:** This is the first large series on dental management of Brazilian patients with HAE. Interdisciplinary evaluation is crucial for proper management of patients with HAE.

### P43 Development and validation of the self-efficacy assessment questionnaire in the management of hereditary angioedema for patients and family caregivers (HAE-SES)

#### Francisco Sánchez-Hernández^1,*^, Iria Dobarrio-Sanz^1^, Jose Granero-Molina^1^, Cayetano Fernández-Sola^1^, Isabel M. Fernández-Medina^1^, Carmen M. Alonso-Castro^2^, Jose M. Hernández-Padilla^1^

##### ^1^Department of Nursing, Physiotherapy and Medicine, University of Almería, Spain; ^2^Department of Allergology, Hospital Meixoeiro, Vigo, Spain

###### **Correspondence:** Francisco Sánchez-Hernández (fransh1988@gmail.com)

*Allergy, Asthma & Clinical Immunology* 2019, **15(Suppl 4):**P43

**Purpose:** Research suggests that most patients and family caregivers lack competence in the management of Hereditary Angioedema (HAE). Self-efficacy is considered to be paramount in the development of people’s competence. The aim of this study is to design and psychometrically assess the pilot version of a scale to measure self-efficacy in the management of HAE by patients and their family caregivers.

**Materials and methods:** The study followed an observational, cross sectional design. Fifty-one participants (30 patients and 21 family caregivers) and 16 experts participated in the study. Firstly, a 32item version of the scale was developed by the researchers. Secondly, a panel of 16 experts in the topic were asked to score the relevance of each item using a Likert-type scale (1 = not relevant;2 = somewhat relevant;3 = quite relevant;4 = very relevant) in order to determine its content validity. The individual item’s content validity index (i-CVI) and the scale’s content validity index (S-CVI) were calculated. Thirdly, the tool’s reliability was tested by exploring its internal consistency calculating the Cronbach alpha coefficient (a) once 51 participants had completed the scale.

**Results:** After the analysis, the pilot version of the HAE-SES was comprised of 29 items grouped into 3 dimensions: “Identification of symptoms and initial decision making”, “Treatment management” and “Post-treatment crisis management and waste disposal”. The HAESES’s content validity proved to be excellent (i-CVIs ranged between 0.8 and 1). All the dimensions of the questionnaire obtained CVI scores higher than 0.9, with an overall score of S-CVI = 0.93. In addition, the excellent reliability of the HAE-SES was evidenced by its internal consistency (a = 0.957).

**Conclusion:** The pilot version of the HAE-SES showed excellent psychometric properties to measure self-efficacy in the management of HAE for patients and family caregivers, allowing the validation process to continue.

### P44 Headache as a symptom of hereditary angioedema

#### Beáta Visy^1,3^, Kinga Viktória Kőhalmi^2,3^, Noémi Andrási^2,3^, Ágnes Holdonner^2,3^, Bettina Ignácz^2,3^, Lilian Varga^2,3^, Henriette Farkas^2,3,*^

##### ^1^Heim Pál Children’s Hospital, Budapest, Hungary; ^2^3^rd^ Department of Internal Medicine, Semmelweis University, Budapest, Hungary; ^3^National Angioedema Reference Center, Budapest, Hungary

###### **Correspondence:** Henriette Farkas (farkas.henriette@med.semmelweis-univ.hu)

*Allergy, Asthma & Clinical Immunology* 2019, **15(Suppl 4):**P44

**Background:** Headache is among the most common symptoms, and a possible accompanying sign of a number of diseases. In the family of primary headache disorders, bradykinin – a vasodilatory substance, which also enhances vascular permeability – might be involved in the pathomechanism of migraine. Bradykinin is the key vasoactive mediator responsible for the symptoms of hereditary angioedema resulting from C1-inhibitor deficiency (C1-INH HAE). In addition to the frequent recurrence of subcutaneous and submucosal edema formation, angioedema has been reported to occur in rare locations including the central nervous system, and cause headache along with neurological symptoms.

Our study assessed the incidence and properties of headache, as well as the possible efficacy of HAE therapies in its relief.

**Materials and methods:** All C1-INH HAE patients attending the annual follow-up visits between 2014 and 2018 were enrolled because since 2014, our practice for taking the medical history also involves administering a questionnaire on headache.

**Results:** In the past five years, 156 patients (63 males and 93 females, mean age 39.2 (1.2 to 86.7 years) returned to the National Angioedema Reference Center for follow-up visits yearly. During this period, 84 of these 156 patients – mostly (71.4%) women – reported headache. Migrain-like features were identified in 17 patients including 13 women.

During follow-up, C1-inhibitor treatment was administered for 39 migraine-like episodes. Five of the six patients (1 male and 5 female, with a mean age of 38.1 years) received plasma-derived C1-INH concentrate (500 to 1500 IU), whereas a single patient was treated with 500 IU plasma-derived and 4200 IU recombinant C1-inhibitor. Except for a single episode during which the patient did not experience any therapeutic effect, the symptoms exhibited substantial regression, and resolved completely in 2 h. No adverse effects occurred. According to the patients, without treatment with C1-INH, migraine-like headache persisted for about half a day or for a full day, and this had a rather negative effect on their well-being.

**Conclusion:** As shown by our experience accumulated during the past 5 years in patients with C1-INH HAE, treatment with C1-INH might prove beneficial in the management of episodic migrain-like headache unresponsive to treatment and accompanied by unilateral neurological signs. Further studies relying on imaging modalities or other biomarkers are necessary to decide whether these episodes are the manifestations of HAE, or the components of a separate, co-occurring disorder.

This study was supported by OTKA K124557.

### P45 Short-term prophylaxis in patients with angioedema due to C1–inhibitor deficiency undergoing dental procedures

#### Andrea Zanichelli^1,*^, Mario Ghezzi^2^, Ivan Santicchia^1^, Romualdo Vacchini^1^, Marco Cicardi^1^

##### ^1^Department of Biomedical and Clinical Sciences Luigi Sacco, University of Milan, ASST Fatebenefratelli Sacco, Milan (MI), Italy; ^2^Odontoiatric Unit, ASST Fatebenefratelli Sacco, Milan (MI), Italy

###### **Correspondence:** Andrea Zanichelli (andrea.zanichelli@unimi.it)

*Allergy, Asthma & Clinical Immunology* 2019, **15(Suppl 4):**P45

**Background:** Patients affected by angioedema due to hereditary and acquired C1-inhibitor deficiency (C1-INH-HAE and C1-INH-AAE, respectively) report troubles in accessing dental care. In patients with C1-INH deficiency, dental procedures can trigger life-threatening laryngeal attacks. Dentists often are not familiar with the management of angioedema attacks and do not dare to treat dental disease in these patients. As a consequence, patients with C1-INH deficiency suffer a lack of proper dental care. Short-term prophylaxis (STP) is recommended by international guidelines [1] before dental procedures, but prospective clinical trials are lacking. It is well known that infections, also localized in the oral cavity, may trigger angioedema attacks [2]. Therefore, improving dental care may be a useful strategy to reduce the frequency of angioedema attacks [3]. The primary endpoints of this study were the assessment of the presence of hurdles in receiving dental care and the effectiveness of short-term prophylaxis in preventing angioedema attacks. The secondary endpoint was the impact of dental care in angioedema course.

**Materials and methods:** All patients affected by angioedema due to C1-INH deficiency treated in the dentistry outpatient department of ASST Fatebenefratelli Sacco hospital in the period 2009–2017 were considered for the analysis. Data on oral status, dental procedures, STP, and disease course were collected prospectively.

**Results:** Twenty-nine patients were analysed (27 with C1-INH-HAE and 2 with C1-INH-AAE). Of them, 58.6% reported hurdles in accessing dental care. At the first visit, 55.17% patients had moderate-to-severe oral disease. Sixty-three dental procedures were performed in 20 patients. Fifty procedures were preceded by STP with plasma derived C1-INH (pdC1-INH) in patients with/without long-term prophylaxis (LTP). One procedure in one C1-INH-HAE patient was preceded by short course of androgens (danazol). Post-procedural attack occurred in 2 patients. One C1-INH-HAE patient undergoing a tooth extraction without STP/LTP experienced a laryngeal attack. The other post-procedural attack occurred in a C1-INH-AAE patient with anti-C1-INH antibodies with STP. Angioedema course did not worsen in any patient after dental care, but improved in 4 of them.

**Conclusions:** Patients had encountered hurdles in accessing dental care, responsible also for the high rate of moderate-to-severe oral disease at the first visit. STP is protective towards attacks after dental procedures in C1-INH-HAE patients. In C1-INH-AAE patients with anti-C1-INH antibodies, STP with pdC1-INH may be nonprotective. Treating oral diseases tends to reduce the frequency of attacks.

**Acknowledgements:** Poster support and styling services were funded by CSL Behring, Italy.

**Consent to publish:** Informed consent to publish has been obtained from these patients.

**Ethics approval:** The study was approved by Comitato Etico Interaziendale Milano Area A in February 26, 2016 with protocol number 3431/2016.

**References**

1. Cicardi M, Aberer W, Banerji A, Bas M, Bernstein JA, Bork K, et al.; HAWK under the patronage of EAACI (European Academy of Allergy and Clinical Immunology). Classification, diagnosis, and approach to treatment for angioedema: consensus report from the Hereditary Angioedema International Working Group. Allergy. 2014; 69:602-16.
2. Farkas H, Zotter Z, Csuka D, Szabó E, Nébenfűhrer Z, Temesszentandrási G, et al. Short-term prophylaxis in hereditary angioedema due to deficiency of the C1-inhibitor–a long-term survey. Allergy. 2012; 67:1586-93.
3. Ceccarini A, Nori A, Fuscà F, Zavaglia V, Marinangeli L, Zoli A. Management of a patient with hereditary angioedema in dentistry: a case report. Dental Cadmos 2014; 82:293-9.

### P46 Mass spectrometry based screening for hereditary angioedema disease

#### Claudia Cozma^1^, Marius Iurascu^1^, Marcus Maurer^2^, Markus Magerl^2^, Toni M. Förster^1^, Volha Skrahina^1,*^, Arndt Rolfs^1,3^

##### ^1^Centogene AG, Rostock, Germany; ^2^Department of Dermatology and Allergy Charité - Universitätsmedizin Berlin, Berlin, Germany; ^3^Neurology Department, Medicine Faculty, University of Rostock, Germany

###### **Correspondence:** Volha Skrahina (Volha.Skrahina@centogene.com)

*Allergy, Asthma & Clinical Immunology* 2019, **15(Suppl 4):**P46

**Background:** Hereditary angioedema (HAE) is an autosomal dominant disease resulting from mutations in the *SERPING1* gene, leading to the deficient (type 1) or nonfunctional (type 2) C1-inhibitor protein. Clinical manifestation in all HAE types include acute attacks of edemas affecting the upper airway, face, extremities, genitals, and gastrointestinal system. In 93% of patients with HAE-caused abdominal pain this is the only manifestation of the disease. Centogene’s epidemiological data show a HAE frequency of 1:17,525 in Turkey and 1:36.600 in Germany. Centogene conducts several HAE related studies to update the prevalence of the disease and develop specific biomarker.

**Analytical methods:** The standard diagnosis procedure of HAE in our laboratory is based on Dry Blood Spot (DBS) filtercard technology that simplifies both the sample collection and the logistics. The diagnostic workflow is a two-tier approach: firstly, the complement proteins are quantified by tandem mass spectrometry (MS/MS) and, secondly, the results are confirmed by genetic analysis. The quantification of the complement proteins is performed in situ by quantifying unique complement peptides for each protein (Complement C4 and Complement 1-Inhibitor peptides) using LC/MRM–MS (liquid chromatography multiple reaction monitoring mass spectrometry).

**Epidemiological studies:** Centogene currently runs the “Epidemiological Study on Hereditary Angioedema” (EHA Study) that aims to test subjects with unclear abdominal pain for HAE and to search for new biomarkers in the blood of positive patients. In a total of 4 years, 5000 participants from 7 countries worldwide will be included. To further strengthen the biomarker development, the “Biometabolic HAE” Study (BioHAE) will include diagnosed HAE patients and check for potential new biomarkers via LC/MRM-MS. The “Hereditary Angioedema Kininogen Assay” Study (HAEKA) plans to characterize cleaved high molecular weight kininogen (cHMWK) as a promising biomarker and to further investigate the effect of a novel medication (lanadelumab) in this setting. Participants will donate blood in timely intervals during an edema attack to understand the cHMWK kinetics in the HAE course.

**Conclusion:** The 2 tier approach (LC/MRM-MS followed by confirmation using NGS) was proven to be a rapid and precise way of identifying type 1 and type 2 HAE patients. Several studies by Centogene will use these techniques to search and characterize novel HAE specific biomarkers.

The studies were approved by University Rostock‘s Ethics Board, approval numbers A2017-0007, A2018-0057, and A2019-0046.

